# Nucleophilic Addition Reactions to 10-Acetonitrilium Derivative of *nido*-Carborane and Intramolecular NH⋯HB Interactions in *N*-Alkyl Amidines 10-RNHC(Me)=NH-7,8-C_2_B_9_H_11_

**DOI:** 10.3390/molecules30040828

**Published:** 2025-02-11

**Authors:** Kirill R. Pakholkov, Ekaterina V. Bogdanova, Marina Yu. Stogniy, Kyrill Yu. Suponitsky, Sergey A. Anufriev, Igor B. Sivaev, Vladimir I. Bregadze

**Affiliations:** A.N. Nesmeyanov Institute of Organoelement Compounds, Russian Academy of Sciences, 28 Vavilov Str., 119991 Moscow, Russia; kirillpakholkov2003@gmail.com (K.R.P.); bogdanovakatte@mail.ru (E.V.B.); kirshik@yahoo.com (K.Y.S.); trueman476@mail.ru (S.A.A.); sivaev@ineos.ac.ru (I.B.S.); bre@ineos.ac.ru (V.I.B.)

**Keywords:** *nido*-carborane, acetonitrilium derivative, amide, iminol, imidates, amidines, synthesis, NMR spectroscopy, single crystal X-ray diffraction, NH⋯HB interactions

## Abstract

The addition reactions of water, alcohols, and primary and secondary amines to the 10-acetonitrilium derivative of *nido*-carborane were studied. Hydrolysis of 10-MeC≡N-7,8-C_2_B_9_H_11_ results in iminol 10-MeC(OH)=HN-7,8-C_2_B_9_H_11_, which, on treatment with a base, gives amide [10-MeC(=O)HN-7,8-C_2_B_9_H_11_]^−^. The reactions of 10-MeC≡N-7,8-C_2_B_9_H_11_ with alcohols lead to imidates 10-ROC(Me)=HN-7,8-C_2_B_9_H_11_ (R = Me, Et) as mixtures of *E*- and *Z*-isomers. In the solid state, 10-MeOC(Me)=HN-7,8-C_2_B_9_H_11_ adopts *E*-configuration. The reactions of 10-MeC≡N-7,8-C_2_B_9_H_11_ with primary amines result in amidines 10-RNHC(Me)=HN-7,8-C_2_B_9_H_11_ (R = Me, Et) as mixtures of *E*- and *Z*-isomers. In the solid state 10-EtNHC(Me)=HN-7,8-C_2_B_9_H_11_ was found to have the *Z*-configuration, which is stabilized by intramolecular N-H⋯H-B interactions between the NH group originating from the primary amine and the BH group of the carborane cage. These interactions are rather strong (3.7 kcal/mol) and are likely to persist in solution. The reactions of 10-MeC≡N-7,8-C_2_B_9_H_11_ with secondary acyclic (Me_2_NH, Et_2_NH) and cyclic (piperidine, morpholine) amines result in amidines 10-R_2_NC(Me)=HN-7,8-C_2_B_9_H_11_ (R = Me, Et; R_2_ = N(CH_2_)_5_, N(CH_2_CH_2_)_2_O) as single isomers, which, according to single crystal X-ray diffraction data, have the *E*-configuration.

## 1. Introduction

Derivatives of polyhedral boron hydrides (boranes, carboranes and metallacarboranes) have found application in a wide variety of fields, from the development of new materials [1,2,3,4,5,6,7,8,9,10] to medicine [11,12,13,14,15,16,17,18,19,20]. This requires the continuous development of new methods for the synthesis of various functional derivatives of polyhedral boron hydrides. As for anionic polyhedral boron hydrides, the most widely used method for their functionalization is based on the nucleophilic opening of their cyclic oxonium derivatives [21,22]. It is especially actively used for the synthesis of boron-containing biologically active compounds [22,23,24,25,26,27,28,29,30]. Another approach that has been in active development recently is based on nucleophilic addition to the activated triple bond of nitrilium derivatives of polyhedral boron hydrides [31]. This approach has been most widely applied for the preparation of derivatives of *closo*-decaborate [B_10_H_10_]^2−^ and *closo*-dodecaborate [B_12_H_12_]^2−^ anions [32,33,34,35,36].

Unlike the *closo*-decaborate and *closo*-dodecaborate anions, derivatives of the 7,8-dicarba-*nido*-undecaborate anion [7,8-C_2_B_9_H_12_]^−^ (*nido*-carborane) are of interest not only in themselves, but also as ligands in the synthesis of various metallacarboranes [37,38,39,40]. Previously, we have studied the addition reactions of various nucleophiles to propionitrilium [10-EtC≡N-7,8-C_2_B_9_H_11_] [41,42,43] and 3-cyanoethoxypropionitrilium [10-NCCH_2_CH_2_O-CH_2_CH_2_C≡N-7,8-C_2_B_9_H_11_] [44] derivatives of the 7,8-dicarba-*nido*-undecaborate anion, whereas the reactions of its simplest acetonitrilium derivative have received little attention. In this contribution, we report the synthesis of iminol, imidates and amidines based on the acetonitrilium derivative of *nido*-carborane [10-MeC≡N-7,8-C_2_B_9_H_11_].

## 2. Results and Discussion

The acetonitrilium derivative of *nido*-carborane [10-MeC≡N-7,8-C_2_B_9_H_11_] (**1**) was prepared the first time by the reaction of the potassium salt K[7,8-C_2_B_9_H_12_] with acetonitrile in the presence of anhydrous FeCl_3_ in refluxing benzene. The reaction resulted in a mixture of 9- and 10-isomers, from which the more stable 10-isomer was isolated in 30% yield [45]. Later, [10-MeC≡N-7,8-C_2_B_9_H_11_] was prepared in 81% yield by the reaction of tetramethylammonium (Me_4_N)[7,8-C_2_B_9_H_12_] with anhydrous AlCl_3_ in acetonitrile in the presence of acetone [46]. Recently, it was found that the 10-acetonitrilium derivative of *nido*-carborane can be prepared in 86% yield by the reaction of the potassium salt K[7,8-C_2_B_9_H_12_] with acetonitrile in the presence of HgCl_2_ in refluxing benzene [41]. In this work, we used the last method to prepare the acetonitrilium derivative of *nido*-carborane.

**Scheme 1:**
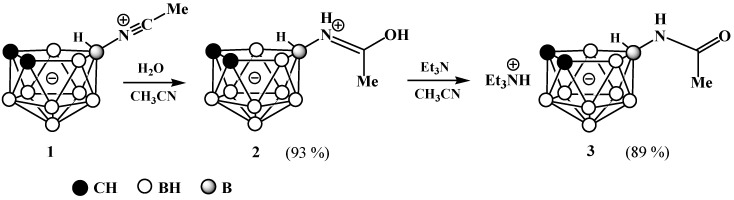
Hydrolysis of the acetonitrilium derivative 10-MeC≡N-7,8-C_2_B_9_H_11_ (**1**).

Hydrolysis of **1** in boiling acetonitrile results in the corresponding *N*-protonated iminol 10-MeC(OH)=NH-7,8-C_2_B_9_H_11_ (**2**), which, on treatment with triethylamine, gives amide (Et_3_NH)[10-MeC(=O)NH-7,8-C_2_B_9_H_11_] (**3**) (Scheme 1). It is worth noting that, according to the ^1^H-^1^H NOESY NMR spectroscopy data (Figure S7), in solution the iminol **2** adopts mainly the *E*-configuration.

**Scheme 2:**
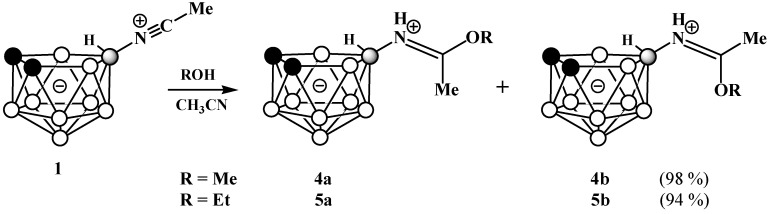
Synthesis of imidates 10-MeOC(Me)=NH-7,8-C_2_B_9_H_11_ (**4**) and 10-EtOC(Me)=HN-7,8-C_2_B_9_H_11_] (**5**).

Reactions of **1** with methanol and ethanol under reflux lead to the corresponding imidates 10-ROC(Me)=NH-7,8-C_2_B_9_H_11_ (R = Me (**4**) and Et (**5**)) in the form of mixtures of *E*- and *Z*-isomers, which, unlike derivatives based on the propionitrile derivative [41], cannot be separated by column chromatography on silica (Scheme 2). This can probably be explained by a decrease in the isomerization energy of the azomethine fragment when the ethyl group is replaced by a smaller methyl group. In an acetone solution, the ratio of *E*- and *Z*-isomers is about 3:1. The assignment of *E*- and *Z*-isomers of the synthesized imidates was performed by analogy with the imidates derived from the propionitrile derivative of *nido*-carborane [41]. In general, in the case of *Z*-isomers, the ^1^H NMR spectral patterns are noticeably broader than in the case of *E*-isomers and the signal of the OR groups in the spectra of *E*-isomers is low-field shifted by ~0.15 ppm, whereas the signals of the Me groups are high-field shifted by ~0.15 ppm relative to those for *Z*-isomers. In particular, the singlets of the MeO and Me groups of the *E*-isomer in the spectrum of 10-MeOC(Me)=NH-7,8-C_2_B_9_H_11_ are located at 4.34 and 2.62 ppm, respectively, while the corresponding signals of the *Z*-isomer are at 4.17 and 2.76 ppm (Figure S16). Similarly, in the spectrum of 10-EtOC(Me)=NH-7,8-C_2_B_9_H_11_, the signals of the methylene group of the EtO fragment and the amidine methyl group of the *E*-isomer are located at 4.66 and 2.58 ppm, respectively, while the corresponding signals of the *Z*-isomer appear at 4.49 and 2.75 ppm (Figure S22). It is worth noting that the differences in the chemical shifts of the signals in the ^11^B NMR spectra of the *E*- and *Z*-isomers of the imidates are insignificant, which leads to an overlapping of the signals with some distortion of their symmetry.

**Figure 1:**
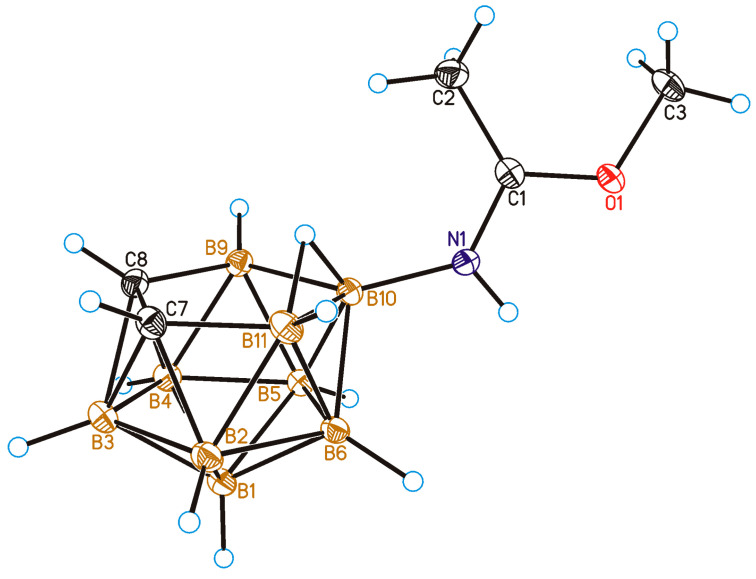
General view of compound 10-MeOC(Me)=NH-7,8-C_2_B_9_H_11_ (**4**) showing atomic numbering. Thermal ellipsoids are drawn at 50% probability level.

The structure of methyl imidate **4** was determined by single crystal X-ray diffraction. In the solid state, 10-MeOC(Me)=NH-7,8-C_2_B_9_H_11_ adopts the *E*-configuration (Figure 1, Table 1). It should be noted that the *E*-configuration in the solid state is characteristic of the iminols based on 7,8-dicarba-*nido*-undecaborate [41,44], *closo*-decaborate [47,48] and *closo*-dodecaborate [36] anions.

**Scheme 3:**
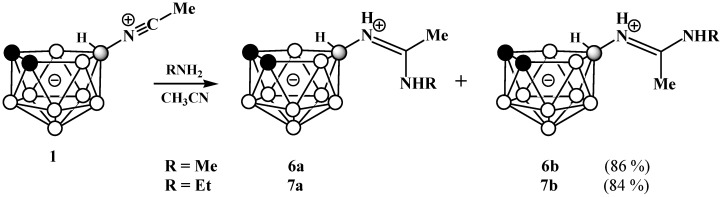
Synthesis of amidines 10-MeNHC(Me)=NH-7,8-C_2_B_9_H_11_ (**6**) and 10-EtNHC(Me)=HN-7,8-C_2_B_9_H_11_] (**7**).

Reactions of **1** with methylamine and ethylamine give the corresponding amidines 10-RNHC(Me)=NH-7,8-C_2_B_9_H_11_ (R = Me (**6**) and Et (**7**)) as mixtures of *E*- and *Z*-isomers, which cannot be separated by column chromatography on silica (Scheme 3).

In an acetone solution, the ratio of *E*- and *Z*-isomers is about 2:1. The behavior of the signals of the Me and RNH groups in the ^1^H NMR spectra of the *E*- and *Z*-isomers of amidines shows the same patterns as in the spectra of the imidates, but the difference in chemical shifts in this case is somewhat larger and amounts to ~0.2 ppm. At the same time, significant differences are observed in the ^11^B NMR spectra of the amidine isomers. A part of the signals is split into two unequal components, the integral ratio of which is almost equal to the ratio of the *E*- and *Z*-isomers determined from the ^1^H NMR spectra. The strongest splitting is observed for the signals corresponding to the boron atoms of the open pentagonal face of *nido*-carborane B(10) and B(9,11), as well as the antipodal boron atoms B(3) and B(2,4), while the signals of the B(1) and B(5,6) atoms remain unchanged. Such changes in the ^11^B NMR spectrum pattern may indicate a significant interaction between the substituent and the BH groups of the pentagonal face of *nido*-carborane, observed for one of the isomers. This is a rather rare case; however, similar intramolecular interactions were observed earlier in the case of 10-dialkylsulfonium derivatives of *nido*-carborane [49].

**Figure 2:**
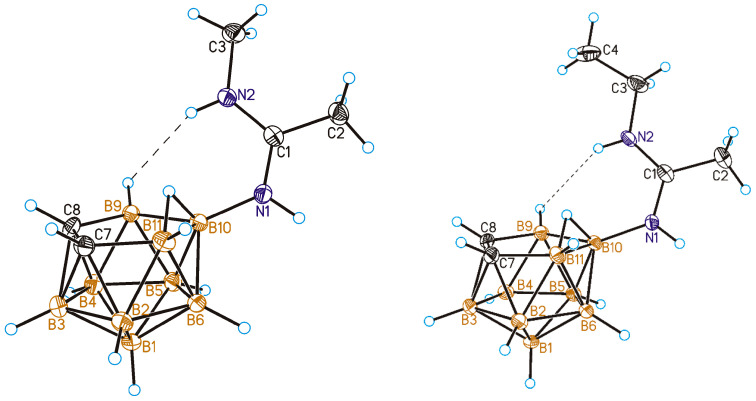
General view of compounds 10-MeNHC(Me)=NH-7,8-C_2_B_9_H_11_ (**6**) (**left**) and 10-EtNHC(Me)=NH-7,8-C_2_B_9_H_11_ (**7**) (**right**) showing atomic numbering. The short N(2)-H⋯H-B(9) contacts are shown by dashed lines. Thermal ellipsoids are drawn at 50% probability level.

Therefore, it was particularly interesting to determine the structure of the obtained *N*-monoalkylamidine derivatives of *nido*-carborane. The structures of *N*-methyl amidine **6** and *N*-ethyl amidine **7** were determined by single crystal X-ray diffraction (Figure 2).

In the solid state, both compounds adopt the *Z*-configuration. There are short intramolecular BH⋯HN contacts between the NH group originating from the primary amine and the BH group of the carborane cage (2.24(3) Å for **6**, and 2.34(2) Å for **7**), which confirms our suggestion. It is worth noting that similar intramolecular BH⋯HN contacts with the *Z*-configuration of the amidine fragment were found earlier in the solid-state structures of *N*-monosubstituted amidines based on *closo*-decaborate [32,34,50,51] and *closo*-dodecaborate [52,53] anions and cobalt and iron bis(dicarbollides) [54,55].

Both the imidate and amidine substituents in the obtained *nido*-carborane derivatives adopt a nearly planar geometry, which implies delocalization of the electron density over this fragment. Indeed, when the weak OR donor group is replaced by a stronger NR’R” donor group an elongation of the N(1)=C(1) double bond and a shortening of the bond B(10)-N(1) are observed (Table 1 and Table 2).

To clarify the role of intramolecular BH⋯HN contacts in the stabilizing the *Z*-configuration of the amidine fragment in the solid state as well as to obtain further confirmation of existence of intramolecular interactions in solution in the case of *N*-alkyl-substituted amidine derivatives and their absence in other cases, we have carried out quantum chemical calculations using PBE0 functional and triple-zeta basis sets, which were utilized in our recent studies on carboranes [56,57]. For each molecule, both the *E*- and *Z*-conformations were calculated. It appeared that for all five molecules, experimentally observed conformation in their crystals is also more energetically preferred in the isolated state. For compounds **4**, **8**–**11** (see below) no strong intramolecular contacts between substituent and carborane cage are observed. Differences in the energy of the *E*- and *Z*-conformations vary in the range of 2–5 kcal/mol. For compounds **6** and **7**, somewhat different results are obtained. In the experimentally observed and energetically preferred *Z*-conformation, relatively strong intramolecular N-H⋯H-B noncovalent contact (4.1 kcal/mol for **6** and 3.7 kcal/mol for **7**, Figure 2) is formed while no intramolecular contacts are found in the *E*-conformation. As a result, the difference between the *E*- and *Z*-conformations is more pronounced and corresponds to 6.9 kcal/mol for **6** and 7.3 kcal/mol for **7**. This is more than sufficient to form the *Z*-conformer exclusively in the solid state, whereas in solution, competition for hydrogen bonding with the solvent will lead to an equilibrium mixture of the *E*- and *Z*-conformers.

**Scheme 4:**
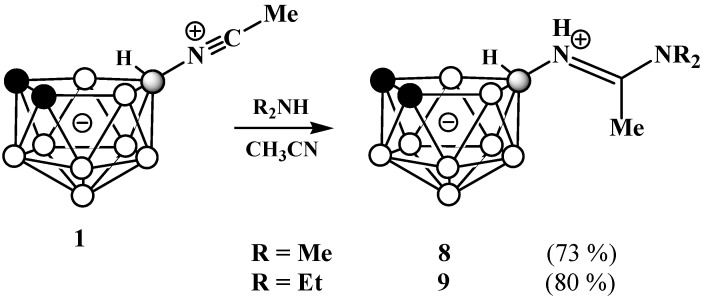
Synthesis of amidines 10-Me_2_NC(Me)=NH-7,8-C_2_B_9_H_11_ (**8**) and 10-Et_2_NC(Me)=HN-7,8-C_2_B_9_H_11_] (**9**).

In contrast to primary amines, the reactions with dimethylamine and diethylamine lead to the corresponding amidines 10-R_2_NC(Me)=NH-7,8-C_2_B_9_H_11_ (R = Me (**8**) and Et (**9**)) as single isomers (Scheme 4).

**Scheme 5:**
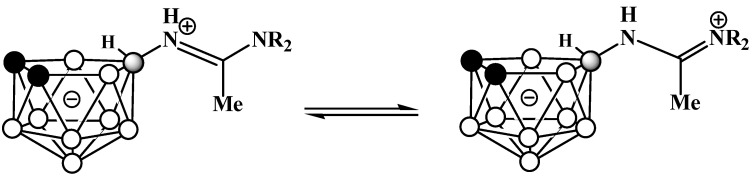
Tautomeric equilibrium in amidines 10-Me_2_NC(Me)=NH-7,8-C_2_B_9_H_11_ and 10-Et_2_NC(Me)=HN-7,8-C_2_B_9_H_11_].

In the ^1^H NMR and ^13^C NMR spectra of amidines **8** and **9**, signals of non-equivalent methyl and ethyl groups, respectively, are observed, which indicates the presence of tautomeric equilibrium (Scheme 5).

**Figure 3:**
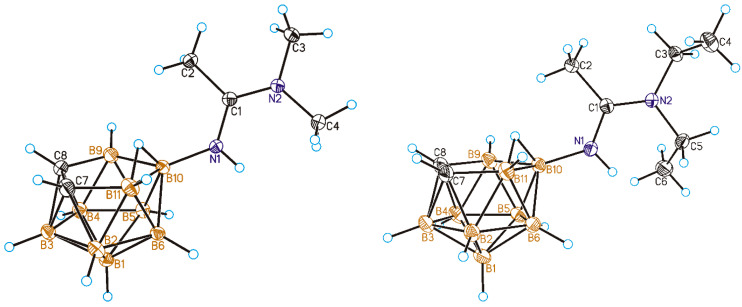
General view of compounds 10-Me_2_NC(Me)=NH-7,8-C_2_B_9_H_11_ (**8**) (**left**) and 10-Et_2_NC(Me)=NH-7,8-C_2_B_9_H_11_ (**9**) (**right**) showing atomic numbering. Thermal ellipsoids are drawn at 50% probability level.

The structures of amidines **8** and **9** were determined by single crystal X-ray diffraction. Both of them have the *E*-conformation (Figure 3, Table 2), which is characteristic of other *N,N*-disubstituted amidines based on 7,8-dicarba-*nido*-undecaborate [42], *closo*-decaborate [58] and *closo*-dodecaborate [36] anions and cobalt bis(dicarbollide) [54].

**Scheme 6:**
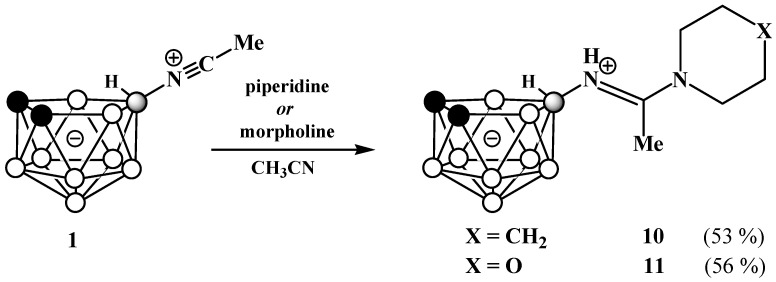
Synthesis of cyclic amidines 10-(CH_2_)_5_NC(Me)=NH-7,8-C_2_B_9_H_11_ (**10**) and 10-O(CH_2_CH_2_)_2_NC(Me)=NH-7,8-C_2_B_9_H_11_ (**11**).

**Figure 4:**
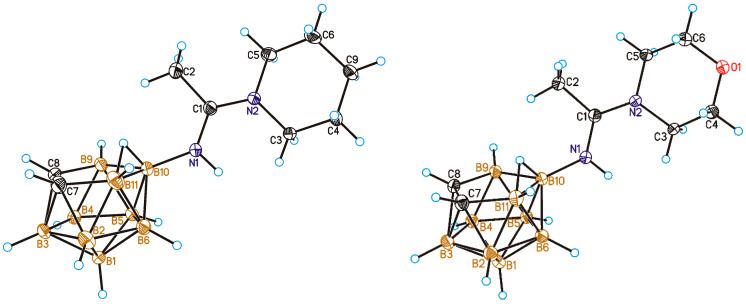
General view of compounds 10-(CH_2_)_5_NC(Me)=NH-7,8-C_2_B_9_H_11_ (**10**) (**left**) and 10-O(CH_2_CH_2_)_2_NC(Me)=NH-7,8-C_2_B_9_H_11_ (**11**) (**right**) showing atomic numbering. Thermal ellipsoids are drawn at 50% probability level.

In a similar way, reactions of the 10-acetonitrilium derivative of *nido*-carborane **1** with piperidine and morpholine lead to the corresponding cyclic amidines 10-(CH_2_)_5_NC(Me)=NH-7,8-C_2_B_9_H_11_ (**10**) and 10-O(CH_2_CH_2_)_2_NC(Me)=NH-7,8-C_2_B_9_H_11_ (**11**) (Scheme 6). The structures of amidines **10** and **11** were determined by single crystal X-ray diffraction (Figure 4).

It was interesting to compare the bond lengths in central fragments of amidine derivatives of various polyhedral boron hydrides. For this purpose, the structure data for amidine derivatives of *nido*-carborane, *closo*-dodecaborate and *closo*-decaborate anions with methyl group and morpholine fragment [B]-NH=C(Me)N(CH_2_CH_2_)_2_O were analyzed. It was found that, whereas in the *nido*-carborane derivative the N(1)=C(1) bond is only slightly shorter than the C(1)-N(2) bond, on moving to the *closo*-dodecaborate and then to the *closo*-decaborate derivatives a noticeable shortening of the first and lengthening of the second bond is observed (Table 3).

The observed regular change in the bond lengths in the amidine fragment is in good agreement with the increase in the electron-donor ability of polyhedral boron hydrides in the series [B_10_H_10_]^2−^ > [B_12_H_12_]_2−_ > [7,8-C_2_B_9_H_12_]^−^, previously discovered based on the ^1^H NMR spectral data of their derivatives [59].

## 3. Materials and Methods

### 3.1. General Methods

The acetonitrilium derivative of *nido*-carborane [10-MeC≡N-7,8-C_2_B_9_H_11_] (**1**) was synthesized as described in the literature [41]. Acetonitrile was dried using standard procedure [60]. Methanol, ethanol, methylamine, ethylamine, dimethylamine, diethylamine, triethylamine, piperidine and morpholine were commercial analytical grade reagents and used without further treatment. All reactions were carried out in air. The reaction progress was monitored by thin layer chromatography (Merck F254 silica gel on aluminum plates) and visualized using 0.5% PdCl_2_ in 1% HCl in aq. MeOH (1:10). The NMR spectra at 400.1 MHz (^1^H), 128.4 MHz (^11^B) and 100.0 MHz (^13^C) were recorded with a Varian Inova-400 spectrometer. The signals in the ^1^H and ^13^C NMR spectra were referenced to Me_4_Si, whereas the signals in the ^11^B NMR spectra were referenced to BF_3_·Et_2_O. All spectra were processed in MestRenova version 6.0.2–5475. When processing ^11^B and ^11^B{^1^H} NMR spectra, the baseline alignment was applied to improve the quality of integration. Infrared spectra were recorded on FSM-2201 (INFRASPEC, Saint Petersburg, Russia) instrument. High resolution mass spectra (HRMS) were measured on a Bruker micrOTOF II instrument using electrospray ionization (ESI). The measurements were made in a positive ion mode (interface capillary voltage—4500 V) or in a negative ion mode (3200 V); mass range was calculated from *m*/*z* 50 to *m*/*z* 3000; external or internal calibration was performed with ESI Tuning Mix, Agilent. A syringe injection was used for solutions in acetonitrile (flow rate 3 mL/min^−1^). Nitrogen was applied as a dry gas; the interface temperature was set at 180 °C.

### 3.2. Synthesis of 10-MeC(OH)=NH-7,8-C_2_B_9_H_11_ *(**2**)*

Water (2 mL) was added to a solution of the acetonitrilium derivative of *nido*-carborane **1** (0.20 g, 1.15 mmol) in acetonitrile (5 mL) and the solution was heated under reflux for 3 h. The reaction mixture was cooled to room temperature and evaporated to dryness in vacuo to obtain 0.21 g (93%) of a white crystalline product. ^1^H NMR (acetone-*d*_6_, ppm): δ 10.99 (1H, br s, OH), 8.62 (1H, br s, N*H*=C), 2.40 (3H, s, C*H*_3_), 1.82 (2H, br s, C*H*_carb_), 3.1–−0.1 (8H, br m, B*H*), −0.45 (1H, br q (1:1:1:1), *J* = 55 Hz, B*H*B). ^13^C NMR (acetone-*d*_6_, ppm): δ 176.7 (NH=*C*), 41.8 (*C*H_carb_), 20.2 (*C*H_3_). ^11^B NMR (acetone-*d*_6_, ppm): δ −10.3 (2B, d, *J* = 132 Hz, B(9,11)), −16.1 (2B, d, *J* = 133 Hz, B(5,6)), −20.3 (1B, d, *J* = 144 Hz, B(3)), −22.6 (2B, d, *J* = 151 Hz, B(2,4)), −25.3 (1B, d, *J* = 51 Hz, B(10)-N), −39.2 (1B, d, *J* = 140 Hz, B(1)). IR (film, cm^−1^): 3345 (ν(O-H)), 3183 (ν(N-H)), 2944 (ν(C-H)), 2536 (ν(B-H)), 1652 (ν(C=N)). HRMS-ESI (*m*/*z*): obsd. 191.2030 [M-H]^+^, calcd. for C_4_H_16_B_9_NO 191.2025 [M-H]^+^.

### 3.3. Synthesis of (Et_3_NH)[10-MeC(=O)NH-7,8-C_2_B_9_H_11_] *(**3**)*

Triethylamine (2 mL) was added to a solution of 2 (0.15 g, 0.78 mmol) in acetonitrile (5 mL) and the solution was heated under reflux for 15 min. The reaction mixture was cooled to room temperature and evaporated to dryness in vacuo to obtain 0.20 g (89%) of a yellow crystalline product. ^1^H NMR (acetone-*d*_6_, ppm): δ 6.02 (1H, br s, N*H*=C), 3.34 (6H, q, *J* = 7.3 Hz, (CH_3_C*H*_2_)_3_NH^+^), 1.89 (3H, s, C*H*_3_), 1.70 (2H, br s, C*H*_carb_), 1.39 (1.41 (9H, t, *J* = 7.3 Hz, (C*H*_3_CH_2_)_3_NH^+^), 2.9–−0.1 (8H, br m, B*H*), −0.48 (1H, br q (1:1:1:1), *J* = 61 Hz, B*H*B). ^13^C NMR (acetone-*d*_6_, ppm): δ 173.9 (NH=*C*), 46.6 ((CH_3_*C*H_2_)_3_NH^+^), 40.3 (*C*H_carb_), 23.9 (*C*H_3_), 8.5 ((*C*H_3_CH_2_)_3_NH^+^). ^11^B NMR (acetone-*d*_6_, ppm): δ −10.2 (2B, d, *J* = 133 Hz, B(9,11)), −16.1 (2B, d, *J* = 132 Hz, B(5,6)), −21.3 (1B, d, *J* = 177 Hz, B(3)), −23.0 (2B, d, *J* = 143 Hz, B(2,4)), −23.5 (1B, s, B(10)-N), −39.5 (1B, d, *J* = 140 Hz, B(1)). IR (film, cm^−1^): 3413 (ν(N–H)), 2999 (ν(C-H)), 2526 (ν(B–H)), 1618 (ν(C=O)). HRMS-ESI (*m*/*z*): obsd. 190.2057 [M+H]^+^, calcd. for C_4_H_15_B_9_NO: 190.2059 [M+H]^+^.

### 3.4. Synthesis of 10-MeOC(Me)=NH-7,8-C_2_B_9_H_11_ *(**4**)*

A solution of the acetonitrilium derivative of *nido*-carborane **1** (0.15 g, 0.86 mmol) in anhydrous methanol (3 mL) was heated under reflux for 2 h. The reaction mixture was cooled to room temperature and evaporated to dryness in vacuo. The residue was subjected to column chromatography on silica with dichloromethane as the eluent to give a white crystalline product, which was a mixture of **4a** and **4b** isomers in a ratio of 3.5:1. Yield 0.17 g (98%). ^11^B NMR (acetone-*d*_6_, ppm): δ −10.3 (2B, d, *J* = 138 Hz, *B*(9,11), **4a**+**4b**), −16.1 (2B, d, *J* = 132 Hz, *B*(5,6), **4a**+**4b**), −20.1 (1B, d, *J* = 162 Hz, *B*(3), **4a**+**4b**), −21.2–−24.4 (2.22B, d, *J* = 153 Hz, *B*(2,4), **4a**+**4b**, *B*(10)-N, **4b**), −25.5 (0.78B, d, *J* = 43 Hz, B(10)-N, **4a**), −39.1 (1B, d, *J* = 140 Hz, *B*(1), **4a**+**4b**). IR (film, cm^−1^): 3354 (ν(N-H)), 2934 (ν(C-H)), 2551 (ν(B-H)), 1637 (ν(C=N)). HRMS-ESI (*m*/*z*): obsd. 224.2608 [M+NH_4_]^+^, calcd. for C_5_H_18_B_9_NO 224.2604 [M+NH_4_]^+^. **4a**: ^1^H NMR (acetone-*d*_6_, ppm): δ 9.04 (1H, br s, N*H*=C), 4.34 (3H, s, OC*H*_3_), 2.62 (3H, s, C*H*_3_), 1.86 (2H, br s, C*H*_carb_), 3.0–−0.1 (8H, br m, B*H*), −0.45 (1H, br q (1:1:1:1), *J* = 59 Hz, B*H*B). ^13^C NMR (acetone-*d*_6_, ppm): δ 178.7 (NH=*C*), 59.0 (O*C*H_3_), 41.8 (*C*H_carb_), 17.32 (*C*H_3_). **4b**: ^1^H NMR (acetone-*d*_6_, ppm): δ 8.50 (1H, br s, N*H*=C), 4.17 (3H, s, OC*H*_3_), 2.76 (3H, s, C*H*_3_), 1.94 (2H, s, C*H*_carb_), 3.0–−0.1 (8H, br m, B*H*), −0.81 (1H, br q (1:1:1:1), *J* = 51 Hz, B*H*B). ^13^C NMR (acetone-*d*_6_, ppm): δ 178.7 (NH=*C*), 57.7 (O*C*H_3_), 41.8 (*C*H_carb_), 17.26 (*C*H_3_).

### 3.5. Synthesis of 10-EtOC(Me)=NH-7,8-C_2_B_9_H_11_ *(**5**)*

The procedure was similar to that described for the synthesis of compound **4** using the acetonitrilium derivative of *nido*-carborane **1** (0.15 g, 0.86 mmol) and anhydrous ethanol (3 mL) to give a white crystalline product, which was a mixture of isomers **5a** and **5b** in a 2.9: 1 ratio. Yield 0.18 g (94%). ^11^B NMR (acetone-*d*_6_, ppm): δ −10.3 (2B, d, *J* = 140 Hz, *B*(9,11), **5a**+**5b**), −16.1 (2B, d, *J* = 133 Hz, *B*(5,6), **5a**+**5b**), −20.1 (1B, d, *J* = 162 Hz, *B*(3), **5a**+**5b**), −21.3–−24.3 (2.26B, d, *J* = 150 Hz, *B*(5,6), **5a**+**5b**, *B*(10)-N, **5b**), −25.4 (0.74B, d, *J* = 45 Hz, B(10)-N, **5a**), −39.2 (1B, d, *J* = 140 Hz, *B*(1), **5a**+**5b**). IR (film, cm^−1^): 3329 (ν(N-H)), 2928 (ν(C-H)), 2540 (ν(B-H)), 1637 (ν(C=N)). HRMS-ESI (*m*/*z*): obsd. 259.2056 [M+K]^+^, calcd. for C_6_H_21_B_9_NO: 259.2057 [M + K]^+^. **5a**: ^1^H NMR (acetone-*d*_6_, ppm): δ 8.93 (1H, br s, N*H*=C), 4.66 (2H, q, *J* = 7.1 Hz, CH_3_C*H*_2_O), 2.58 (3H, s, C*H*_3_), 1.93 (2H, br s, C*H*_carb_), 1.51 (3H, t, *J* = 7.0 Hz, C*H*_3_CH_2_O) 2.9–−0.1 (8H, br m, B*H*), −0.40 (1H, br q (1:1:1:1), *J* = 51 Hz, B*H*B). ^13^C NMR (acetone-*d*_6_, ppm): δ 177.7 (NH=*C*), 69.0 (CH_3_*C*H_2_O), 41.7 (*C*H_carb_), 17.6 (*C*H_3_CH_2_O), 13.9 (*C*H_3_). **5b**: ^1^H NMR (acetone-*d*_6_, ppm): δ 8.40 (1H, br s, N*H*), 4.49 (2H, q, *J* = 7.0 Hz, CH_3_C*H*_2_O), 2.75 (3H, s, C*H*_3_), 1.93 (2H, br s, C*H*_carb_), 1.38 (3H, t, *J* = 7.0 Hz, C*H*_3_CH_2_O) 2.9–−0.1 (8H, br m, B*H*), −0.9 (1H, br q (1:1:1:1), *J* = 56 Hz, B*H*B). ^13^C NMR (acetone-*d*_6_, ppm): δ 178.9 (NH=*C*), 67.6 (CH_3_*C*H_2_O), 41.7 (*C*H_carb_), 17.5 (*C*H_3_CH_2_O), 13.2 (*C*H_3_).

### 3.6. Synthesis of 10-MeNHC(Me)=NH-7,8-C_2_B_9_H_11_ *(**6**)*

Methylamine (2 mL) was added to a −30 °C solution of the acetonitrilium derivative of *nido*-carborane **1** (0.20 g, 1.15 mmol) in acetonitrile (5 mL), the reaction mixture was allowed to warm to room temperature and stirred for 15 min. The reaction mixture was evaporated to dryness in vacuo and the residue was subjected to column chromatography on silica with dichloromethane as the eluent to give a white crystalline product, which was a mixture of **6a** and **6b** isomers in a ratio of 1.6:1. Yield 0.20 g (86%). ^11^B NMR (acetone-*d*_6_, ppm): δ −10.6 (0.77B, d, *J* = 139 Hz, *B*(9,11), **6b**), −12.3 (1.23B, d, *J* = 139 Hz, *B*(9,11), **6a**), −15.2 (2B, d, *J* = 136 Hz, *B*(5,6), **6a**+**6b**), −20.0–−23.5 (3.38 B, m, *B*(2,3,4), **6a**+**6b** + *B*(10)-N, **6b**,), −24.2 (0.62B, s, B(10)-N, **6a**), −39.1 (1B, d, *J* = 141 Hz, *B*(1), **6a**+**6b**). IR (film, cm^−1^): 3397 (ν(N-H)), 3344 (ν(N-H)), 2934 (ν(C-H)), 2862 (ν(C-H)), 2532 (ν(B-H)), 1637 (ν(C=N)). HRMS-ESI (*m*/*z*): obsd. 223.2776 [M+NH_4_]^+^, calcd. for C_5_H_19_B_9_N_2_: 223.2764 [M+NH_4_]^+^. **6a**: ^1^H NMR (acetone-*d*_6_, ppm): δ 8.17 (1H, br s, N*H*CH_3_), 7.23 (1H, br s, N*H*=C), 3.17 (3H, s, NHC*H*_3_), 2.31 (3H, s, C*H*_3_), 1.87 (2H, br s, C*H*_carb_), 2.9–−0.1 (8H, br m, *BH*), −1.32 (1H, br q (1:1:1:1), *J* = 52 Hz, B*H*B). ^13^C NMR (acetone-*d*_6_, ppm): δ 168.7 (NH=*C*), 42.3 (*C*H_carb_), 29.8 (NH*C*H_3_), 17.6 (*C*H_3_). **6b**: ^1^H NMR (acetone-*d*_6_, ppm): δ 8.38 (1H, br s, N*H*CH_3_), 6.22 (1H, br s, N*H*=C), 2.94 (3H, s, NHC*H*_3_), 2.57 (3H, s, C*H*_3_), 1.87 (2H, br s, C*H*_carb_), 2.9–−0.1 (8H, br m, *BH*), −0.99 (1H, br q (1:1:1:1), *J* = 52 Hz, B*H*B). ^13^C NMR (acetone-*d*_6_, ppm): δ 153.1 (NH=*C*), 42.3 (*C*H_carb_), 27.6 (NH*C*H_3_), 19.1 (*C*H_3_).

### 3.7. Synthesis of 10-EtNHC(Me)=NH-7,8-C_2_B_9_H_11_ *(**7**)*

The procedure was similar to that described for the synthesis of compound **6** using the acetonitrilium derivative of *nido*-carborane (0.20 g, 1.15 mmol) and ethylamine (2 mL) to give a white crystalline product, which was a mixture of **7a** and **7b** isomers in a ratio of 2.1:1. Yield 0.21 g (84%). ^11^B NMR (acetone-*d*_6_, ppm): δ −10.7 (0.65B, d, *J* = 138 Hz, *B*(9,11), **7b**), −12.3 (1.35B, d, *J* = 141 Hz, *B*(9,11), **7a**), −15.3 (2B, d, *J* = 135 Hz, *B*(5,6), **7a**+**7b**), −20.0–−23.5 (3.33B, m, *B*(2,3,4), **7a**+**7b** + *B*(10)-N, **7b**), −24.2 (0.68B, s, B(10)-N, **7a**), −39.0 (1B, d, *J* = 141 Hz, *B*(1), **7a**+**7b**). IR (film, cm^−1^): 3394 (ν(N-H)), 2928 (ν(C-H)), 2548 (ν(B-H)), 1642 (ν(C=N)). HRMS-ESI (*m*/*z*): obsd. 237.2925 [M+NH_4_]^+^, calcd. for C_6_H_21_B_9_N_2_: 237.2921 [M+NH_4_]^+^. **7a**: ^1^H NMR (acetone-*d*_6_, ppm): δ 8.14 (1H, br s, N*H*CH_2_CH_3_), 7.23 (1H, br s, N*H*=C), 3.57 (2H, quint, *J* = 6.4 Hz, NHC*H_2_*CH_3_), 2.33 (3H, s, C*H_3_*), 1.90 (2H, br s, C*H*_carb_), 1.32 (3H, t, *J* = 7.2 Hz, NHCH_2_C*H*_3_) 2.9–−0.1 (8H, br m, B*H*), −1.30 (1H, br q (1:1:1:1), *J* = 50 Hz, B*H*B). ^13^C NMR (acetone-*d*_6_, ppm): δ 167.1 (NH=*C*), 42.5 (C*H*_carb_), 38.7 (NH*C*H_2_CH_3_), 17.4 (*C*H_3_), 14.2 (NHCH_2_*C*H_3_). **7b**: ^1^H NMR (acetone-*d*_6_, ppm): δ 8.24 (1H, br s, N*H*CH_2_CH_3_), 6.22 (1H, br s, N*H*=C), 3.35 (2H, quint, *J* = 6.4 Hz, NHC*H*_2_CH_3_), 2.57 (3H, s, C*H*_3_), 1.86 (2H, br s, C*H*_carb_), 1.21 (3H, t, *J* = 7.1 Hz, NHCH_2_C*H*_3_) 2.9–−0.1 (8H, br m, B*H*), −0.97 (1H, br q (1:1:1:1), *J* = 50 Hz, B*H*B). ^13^C NMR (acetone-*d*_6_, ppm): δ 153.3 (NH=*C*), 42.5 (*C*H_carb_), 36.3 (NH*C*H_2_CH_3_), 19.2 (*C*H_3_), 12.3 (NHCH_2_*C*H_3_).

### 3.8. Synthesis of 10-Me_2_NC(Me)=NH-7,8-C_2_B_9_H_11_ *(**8**)*

The procedure was similar to that described for the synthesis of compound **6** using the acetonitrilium derivative of *nido*-carborane (0.20 g, 1.15 mmol) and dimethylamine (2 mL) to give white solid **8**. Yield 0.18 g (73%). ^1^H NMR (acetone-*d*_6_, ppm): δ 5.89 (1H, br s, N*H*=C), 3.33 (3H, s, NC*H*_3_), 3.14 (3H, s, NC*H*_3_), 2.71 (3H, s, CH_3_), 1.87 (2H, br s, C*H*_carb_), 2.8–−0.1 (8H, br m, B*H*), −1.03 (1H, br q (1:1:1:1), *J* = 60 Hz, B*H*B). ^13^C NMR (acetone-*d*_6_, ppm): δ 167.3 (N=*C*), 41.8 (*C*H_carb_), 40.6 (N*C*H_3_), 37.6 (N*C*H_3_),16.3 (*C*H_3_). ^11^B NMR (acetone-*d*_6_, ppm): δ −10.8 (2B, d, *J* = 139 Hz, B(9,11)), −15.1 (2B, d, *J* = 136 Hz, (B5,6)), −21.1 (1B, d, *J* = 146 Hz, (B3)), −22.0 (3B, d, *J* = 124 Hz, (B(2,4) + *B*(10)-N), −39.0 (1B, d, *J* = 142 Hz, B(1)). IR (film, cm^−1^): 3401 (ν(N-H)), 2962 (ν(C-H)), 2837 (ν(C-H)), 2549 (ν(B-H)), 1652 (ν(C=N)). HRMS-ESI (*m*/*z*); obsd. 217.2542 [M+H]^+^, calcd. for C_6_H_21_B_9_N_2_: 217.2532 [M+H]^+^.

### 3.9. Synthesis of 10-Et_2_NC(Me)=NH-7,8-C_2_B_9_H_11_ *(**9**)*

Diethylamine (2 mL) was added to a solution of the acetonitrilium derivative of *nido*-carborane **1** (0.20 g, 1.15 mmol) in acetonitrile (5 mL) and the reaction mixture was stirred for 15 min. The reaction mixture was evaporated to dryness in vacuo and the residue was subjected to column chromatography on silica with dichloromethane as the eluent to give white product **9**. Yield 0.30 g (80%). ^1^H NMR (acetone-*d*_6_, ppm): δ 5.84 (1H, br s, N*H*=C), 3.63 (2H, q, *J* = 7.2 Hz, NC*H*_2_CH_3_), 3.56 (2H, q, *J* = 7.2 Hz, NC*H*_2_CH_3_), 2.72 (3H, s, C*H*_3_), 1.88 (2H, br s, C*H*_carb_), 1.28 (3H, t, *J* = 7.2 Hz, NCH_2_C*H*_3_), 1.15 (3H, t, *J* = 7.2 Hz, NCH_2_C*H*_3_), 2.8–−0.1 (8H, br m, B*H*), −1.00 (1H, br q (1:1:1:1), *J* = 59 Hz, B*H*B). ^13^C NMR (acetone-*d*_6_, ppm): δ 165.7 (N=*C*), 46.5 (N*C*H_2_CH_3_), 42.6 (N*C*H_2_CH_3_), 41.9 (*C*H_carb_), 16.2 (*C*H_3_) 12.8 (N*C*H_2_CH_3_), 10.9 (NCH_2_*C*H_3_). ^11^B NMR (acetone-*d*_6_, ppm): δ −10.8 (2B, d, *J* = 138 Hz, *B*(9,11)), −15.1 (2B, d, *J* = 136 Hz, *B*(5,6)), −21.0 (1B, d, *J* = 142 Hz, *B*(3)), −22.0 (3B, d, *J* = 128 Hz, *B*(2,4) *+ B*(10)-N), −39.1 (1B, d, *J* = 142 Hz, *B*(1)). IR (film, cm^−1^): 3416 (ν(N-H)), 2990 (ν(C-H)), 2529 (ν(B-H)), 1605 (ν(C=N)). HRMS-ESI (*m*/*z*): obsd. 245.2839 [M+H]^+^, calcd. for C_8_H_25_B_9_N_2_: 245.2845 [M+H]^+^.

### 3.10. Synthesis of 10-(CH_2_)_5_NC(Me)=NH-7,8-C_2_B_9_H_11_ *(**10**)*

The procedure was similar to that described for the synthesis of compound **9** using the acetonitrilium derivative of *nido*-carborane (0.29 g, 1.67 mmol) and piperidine (2 mL) to give white solid **10**. Yield 0.23 g (53%). ^1^H NMR (acetone-*d*_6_, ppm): δ 6.16 (1H, br s, N*H*=C), 3.70 (2H, m, NC*H*_2_CH_2_CH_2_), 3.57 (2H, m, NC*H*_2_CH_2_CH_2_), 2.74 (3H, s, C*H*_3_), 1.87 (2H, br s, C*H*_carb_), 1.71 (4H, m, NCH_2_C*H*_2_CH_2_ + NCH_2_CH_2_C*H*_2_), 1.64 (2H, m, NCH_2_C*H_2_*CH_2_), 2.9–−0.1 (8H, br m, B*H*), −1.02 (1H, br q (1:1:1:1), *J* = 58 Hz, B*H*B). ^13^C NMR (acetone-*d*_6_, ppm): δ 165.3 (N=*C*), 49.5 (N*C*H_2_CH_2_CH_2_), 45.7 (N*C*H_2_CH_2_CH_2_), 41.9 (*C*H_carb_), 25.7 (NCH_2_*C*H_2_CH_2_), 25.0 (NCH_2_*C*H_2_CH_2_), 23.3 (NCH_2_CH_2_*C*H_2_), 16.5 (*C*H_3_). ^11^B NMR (acetone-*d*_6_, ppm): δ −10.9 (2B, d, *J* = 137 Hz, *B*(9,11)), −15.2 (2B, d, *J* = 136 Hz, *B*(5,6)), −21.1 (1B, d, *J* = 136 Hz, *B*(3)), −21.9 (3B, d, *J* = 134 Hz, *B*(2,4) + *B*(10)-N), −39.1 (1B, d, *J* = 141 Hz, *B*(1)). IR (film, cm^−1^): 3416 (ν(N-H)), 2959 (ν(C-H)), 2514 (ν(B-H)), 1608 (ν(C=N)). HRMS-ESI (*m*/*z*): obsd. 257.2846 [M+H]^+^, calcd. for C_9_H_25_B_9_N_2_: 257.2845 [M+H]^+^.

### 3.11. Synthesis of 10-O(CH_2_CH_2_)_2_NC(Me)=NH-7,8-C_2_B_9_H_11_ *(**11**)*

The procedure was similar to that described for the synthesis of compound **9** using the acetonitrilium derivative of *nido*-carborane (0.27 g, 1.56 mmol) and morpholine (2 mL) to give white solid **11**. Yield 0.23 g (56%). ^1^H NMR (acetone-*d*_6_, ppm): δ 6.33 (1H, br s, N*H*=C), 3.78 (4H, m, OC*H*_2_), 3.73 (2H, m, NC*H*_2_), 3.64 (2H, m, NC*H*_2_), 2.77 (3H, s, C*H*_3_), 1.88 (2H, br s, C*H*_carb_), 3.0–−0.1 (8H, br m, B*H*), −1.00 (1H, br q (1:1:1:1), *J* = 59 Hz, B*H*B). ^13^C NMR (acetone-*d*_6_, ppm): δ 166.6 (N=*C*), 65.8 (O*C*H_2_), 65.2 (O*C*H_2_), 48.2 (N*C*H_2_), 44.7 (N*C*H_2_), 42.0 (*C*H_carb_), 16.3 (*C*H_3_). ^11^B NMR (acetone-*d*_6_, ppm): δ −10.9 (2B, d, *J* = 142 Hz, *B*(9,11)), −15.3 (2B, d, *J* = 136 Hz, *B*(5,6)), −20.9 (1B, d, *J* = 152 Hz, *B*(3)), −22.0 (3B, d, *J* = 130 Hz, *B*(2,4) + *B*(10)-N), −39.0 (1B, d, *J* = 142 Hz, *B*(1)). IR (film, cm^−1^): 3374 (ν(N-H)), 2934 (ν(C-H)), 2517 (ν(B-H)), 1608 (ν(C=N)). HRMS-ESI (*m*/*z*): obsd. 259.2646 [M+H]^+^, calcd. for C_8_H_23_B_9_N_2_O: 259.2638 [M+H]^+^.

### 3.12. Single Crystal X-Ray Diffraction Study

Single crystal X-ray diffraction experiments for compounds **4** and **6**–**11** were carried out using a SMART APEX2 CCD diffractometer (λ(Mo-Kα) = 0.71073 Å, graphite monochromator, ω-scans) at 100 K. Collected data were processed by the SAINT and SADABS programs incorporated into the APEX2 program package [61]. The structures were solved by the direct methods and refined by the full-matrix least-squares procedure against *F*^2^ in anisotropic approximation. The refinement was carried out with the SHELXTL program [62]. The details of data collection and crystal structure refinement are summarized in Tables S1 and S2 along with CCDC numbers which contain the supplementary crystallographic data for this paper. These data can be obtained free of charge via www.ccdc.cam.ac.uk/data_request/cif.

### 3.13. Quantum Chemical Calculations

All quantum chemical calculations were carried out with the Gaussian 09 program [63]. The PBE0 functional with the triple zeta basis set was found to be reliable for the optimization of molecular geometries of different families of compounds and calculation of noncovalent intra- and intermolecular interactions [49,56,64,65] and was adopted throughout this study.

All calculated molecular conformers were fully optimized and converged to the true energy minima. Theoretical electron density was treated within the AIM approach [66] using the AIMAll program package [67]. For the estimation of energy of noncovalent atom–atom contact, we used the E = 1/2V(r) formula [68,69], in which V(r) is the potential energy density at the bond critical point between interacting atoms. It has frequently been shown that this approach demonstrates realistic energetic characteristics for noncovalent interactions [49,56,64,65,70].

## 4. Conclusions

The addition reactions of various nucleophiles to the 10-acetonitrilium derivative of *nido*-carborane were studied. Hydrolysis of 10-MeC≡N-7,8-C_2_B_9_H_11_ leads to the *N*-protonated iminol 10-MeC(OH)=HN-7,8-C_2_B_9_H_11_, which, on treatment with a base, gives amide [10-MeC(=O)HN-7,8-C_2_B_9_H_11_]^−^. The reactions of 10-MeC≡N-7,8-C_2_B_9_H_11_ with alcohols result in imidates 10-ROC(Me)=HN-7,8-C_2_B_9_H_11_ (R = Me, Et) as mixtures of *E*- and *Z*-isomers. In the solid state, 10-MeOC(Me)=HN-7,8-C_2_B_9_H_11_ was found to adopt the *E*-conformation. The reactions of 10-MeC≡N-7,8-C_2_B_9_H_11_ with primary amines give amidines 10-RNHC(Me)=HN-7,8-C_2_B_9_H_11_ (R = Me, Et) as mixtures of *E*- and *Z*-isomers. The single crystal X-ray diffraction study revealed that in the solid state 10-EtNHC(Me)=HN-7,8-C_2_B_9_H_11_ and 10-EtNHC(Me)=HN-7,8-C_2_B_9_H_11_ have the *Z*-conformation, which is stabilized by intramolecular N-H⋯H-B interactions between the NH group originating from the primary amine and the BH group of the carborane cage. These interactions are rather strong (3.7–4.1 kcal/mol) and are likely to persist in solution. The reactions of 10-MeC≡N-7,8-C_2_B_9_H_11_ with secondary acyclic (Me_2_NH, Et_2_NH) and cyclic (piperidine, morpholine) amines result in amidines 10-R_2_NC(Me)=HN-7,8-C_2_B_9_H_11_ (R = Me, Et; R_2_ = N(CH_2_)_5_, N(CH_2_CH_2_)_2_O) as single isomers, which according to single crystal X-ray diffraction data have the *E*-conformation.

## Figures and Tables

**Table 1:** Selected geometrical parameters of molecules **4**, **6**, and **7**. Comparison of experimental and calculated results for corresponding conformations of substituents.

Bond (Å) or Angle (deg)	Compound 4 (X = O(1))		Compound 6 (X = N(2))		Compound 7 (X = N(2))	
	X-Ray	Calcd.	X-Ray	Calcd.	X-Ray	Calcd.
B(10)-N(1)	1.528(2)	1.533	1.513(3)	1.523	1.514(2)	1.522
N(1)=C(1)	1.294(2)	1.286	1.315(3)	1.308	1.318(2)	1.309
C(1)-X	1.313(2)	1.318	1.324(3)	1.321	1.322(2)	1.321
B(11)-B(10)-N(1)-C(1)	−104.1(2)	−108.8	−102.1(3)	1142.4	−101.8(2)	−137.3
B(10)-N(1)-C(1)-X	179.5(2)	−179.4	−3.0(4)	3.2	−4.4(2)	2.9
B(10)-N(1)-C(1)-C(2)	−2.1(3)	1.1	175.9(2)	−176.7	175.7(2)	−177.3
N(1)-C(1)-X-C(3)	−177.4(2)	179.1	176.9(2)	−177.6	175.2(2)	−175.9

**Table 2:** Selected geometrical parameters of molecules **8**–**11**. Comparison of experimental and calculated results for corresponding conformations of substituents.

Bond (Å) or Angle (deg)	Compound 8		Compound 9		Compound 10			Compound 11	
	X-Ray	Calc.	X-Ray	Calc.	X-Ray Mol. A	X-Ray Mol. A’	Calc.	X-Ray	Calc.
B(10)-N(1)	1.518(2)	1.529	1.516(2)	1.528	1.518(2)	1.518(2)	1.530	1.520(2)	1.531
N(1)=C(1)	1.329(2)	1.306	1.324(2)	1.307	1.319(2)	1.319(2)	1.306	1.325(2)	1.303
C(1)-N(2)	1.324(2)	1.337	1.330(2)	1.337	1.331(2)	1.331(2)	1.342	1.330(2)	1.344
B(11)-B(10)-N(1)-C(1)	−102.7(2)	−109.2	−96.4(2)	−114.8	−93.8(2)	−97.3(2)	−109.5	−87.1(2)	−108.3
B(10)-N(1)-C(1)-N(2)	175.9(2)	−177.2	−173.6(2)	−176.9	177.5(2)	167.4(2)	175.9	168.9(2)	175.4
B(10)-N(1)-C(1)-C(2)	−4.3(2)	2.7	4.5(2)	3.0	−2.3(3)	−13.9(2)	−3.4	−10.2(2)	−4.0
N(1)-C(1)-N(2)-C(3)	176.4(2)	−175.6	−160.4(2)	179.7	5.2(2)	−7.6(2)	−0.7	−0.5(2)	2.2
N(1)-C(1)-N(2)-C(4)	−3.5(2)	2.8	-	-	-	-	-	-	-
N(1)-C(1)-N(2)-C(5)	-	-	10.7(2)	0.2	170.2(2)	-176.5(2)	165.3	171.3(2)	163.8

**Table 3:** Selected bond lengths in amidine derivatives of polyhedral boron hydrides [B]-NH=C(Me)N(CH_2_CH_2_)_2_O.

[B]	B-N(1)H, Å	N(1)H=C(1), Å	C(1)-N(2), Å	Ref.
*closo*-decaboran-2-yl	1.5366(19)	1.3079(18)	1.3455(17)	[58]
*closo*-dodecaboranyl	1.5309(15)	1.3177(13)	1.3400(13)	[36]
*nido*-carboran-10-yl	1.5202(16)	1.3254(16)	1.3295(15)	this work

## Data Availability

Crystallographic data for the structures of 10-MeOC(Me)=NH-7,8-C_2_B_9_H_11_ (**4**), 10-MeNHC(Me)=NH-7,8-C_2_B_9_H_11_ (**6**), 10-EtNHC(Me)=NH-7,8-C_2_B_9_H_11_ (**7**), 10-Me_2_NC(Me)=NH-7,8-C_2_B_9_H_11_ (**8**), 10-Et_2_NC(Me)=NH-7,8-C_2_B_9_H_11_ (**9**), 10-(CH_2_)_5_NC(Me)=NH-7,8-C_2_B_9_H_11_ (**10**), and 10-O(CH_2_CH_2_)_2_NC(Me)=NH-7,8-C_2_B_9_H_11_ (**11**) were deposited in the Cambridge Crystallographic Data Centre as supplementary publications CCDC 2413512 (for **4**), 2422305 (for **6**), 2413510 (for **7**), 2413511 (for **8**), 2413509 (for **9**), 2422304 (for **10**), and 2413508 (for **11**). The Supplementary Materials contain NMR spectra for compounds **2**–**11**.

## Supplementary Materials

The following supporting information can be downloaded at: https://www.mdpi.com/article/10.3390/molecules30040828/s1, Figures S1–S66: NMR spectra of compounds **2**–**11**; Table S1: Crystallographic data for compounds **4**, **6**, and **7**; Table S2: Crystallographic data for compounds **8**–**11**.
