# Supplementary material for: Nucleophilic Addition Reactions to 10-Acetonitrilium Derivative of nido-Carborane and Intramolecular NH⋯HB Interactions in N-Alkyl Amidines 10-RNHC(Me)=NH-7,8-C2B9H11
Source: Molecules. 2025 Feb 11;30(4):828. doi: 10.3390/molecules30040828 (PMC11858720; doi:10.3390/molecules30040828)
Supplement: Supplementary file 1 [file molecules-30-00828-s001.zip › molecules-3431457-supplementary.pdf]

Supplementary Materials

Nucleophilic Addition Reactions to 10-Acetonitrilium Derivative of *nido*-Carborane. Intramolecular NH···HB Interactions in *N*-Alkyl Amidines 10-RNHC(Me)=NH-7,8-$C_2B_9H_{11}$

Kirill R. Pakholkov, Ekaterina V. Bogdanova, Marina Yu. Stogniy, Kyrill Yu. Suponitsky, Sergey A. Anufriev, Igor B. Sivaev and Vladimir I. Bregadze

NMR spectra in acetone-*d*$_6$....................2

10-MeC(OH)=HN-7,8-$C_2B_9H_{11}$ (2)....................2

Et_3NH[10-MeC(=O)=HN-7,8-$C_2B_9H_{11}$] (3)....................6

10-MeC(OMe)=HN-7,8-$C_2B_9H_{11}$ (4)....................10

10-MeC(OEt)=HN-7,8-$C_2B_9H_{11}$ (5)....................13

10-MeC(NHMe)=HN-7,8-$C_2B_9H_{11}$ (6)....................17

10-EtC(NHEt)=HN-7,8-$C_2B_9H_{11}$ (7)....................21

10-MeC(NMe_2)=HN-7,8-$C_2B_9H_{11}$ (8)....................25

10-MeC(NEt_2)=HN-7,8-$C_2B_9H_{11}$ (9)....................29

10-MeC($N(CH_2)_5$)=HN-7,8-$C_2B_9H_{11}$ (10)....................33

10-MeC($N(CH_2)_2O(CH_2)_2$)=HN-7,8-$C_2B_9H_{11}$ (11)....................37

Crystallographic data for compounds 4, 6-11....................40

NMR spectra in acetone-d_6

10-MeC(OH)=HN-7,8-$C_2B_9H_{11}$ (2)

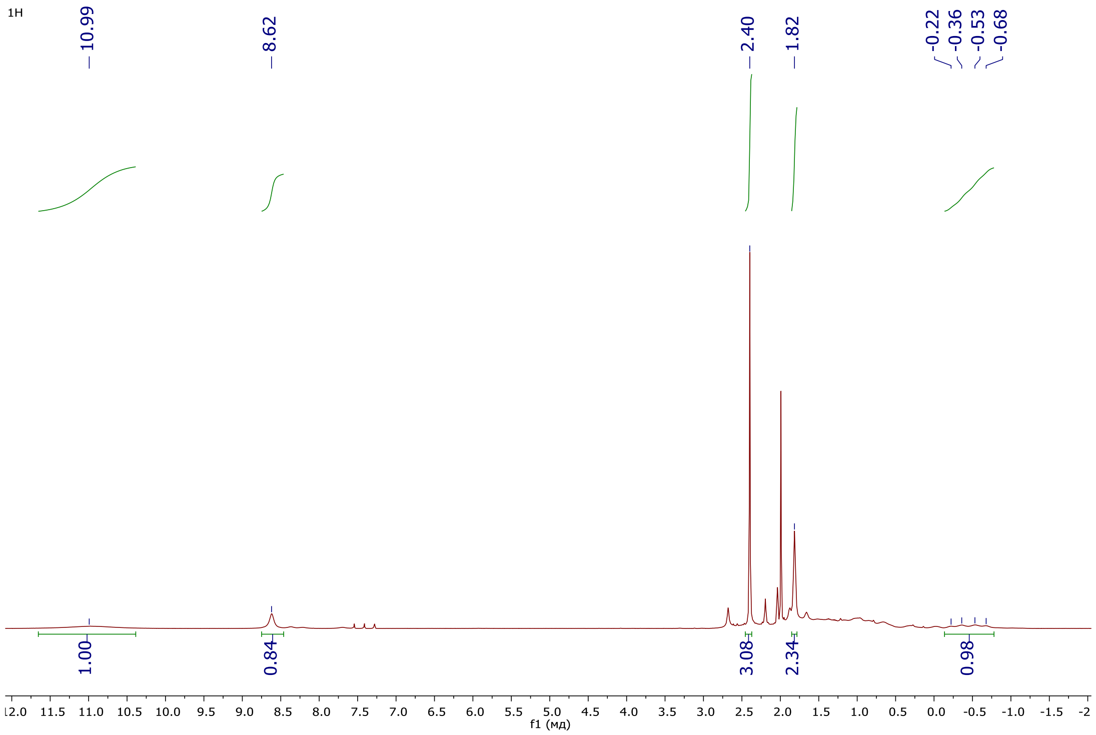

Fig. S1. ^{1}H NMR spectrum of compound **2**.

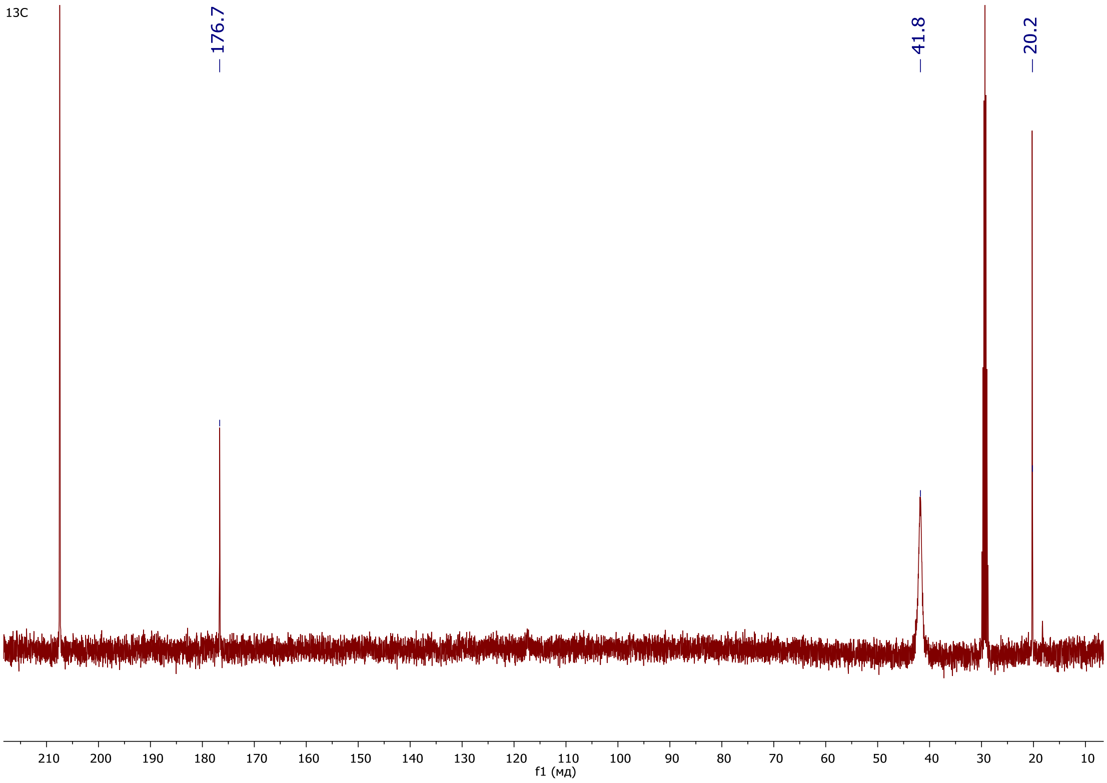

Fig. S2. ^{13}C NMR spectrum of compound **2**.

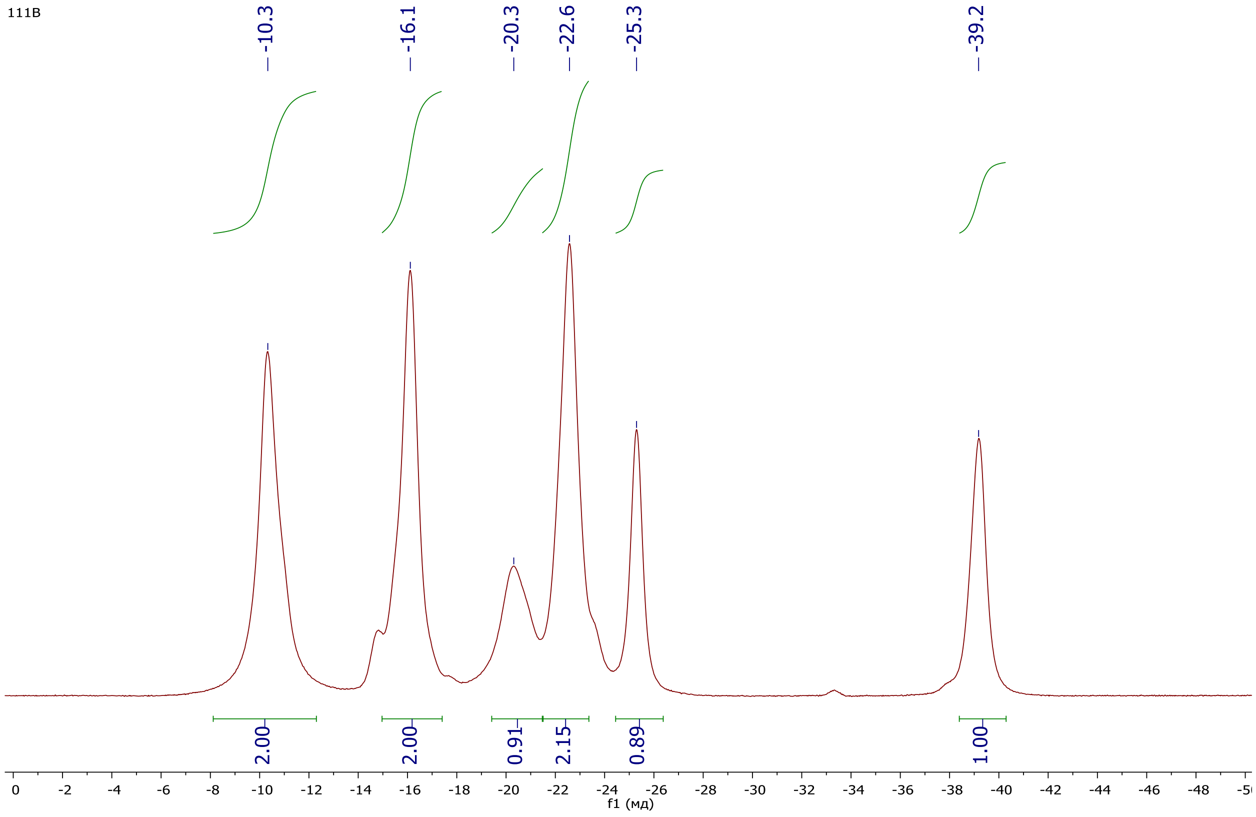

Fig. S3. $^{11}B\{^1H\}$ NMR spectrum of compound **2**.

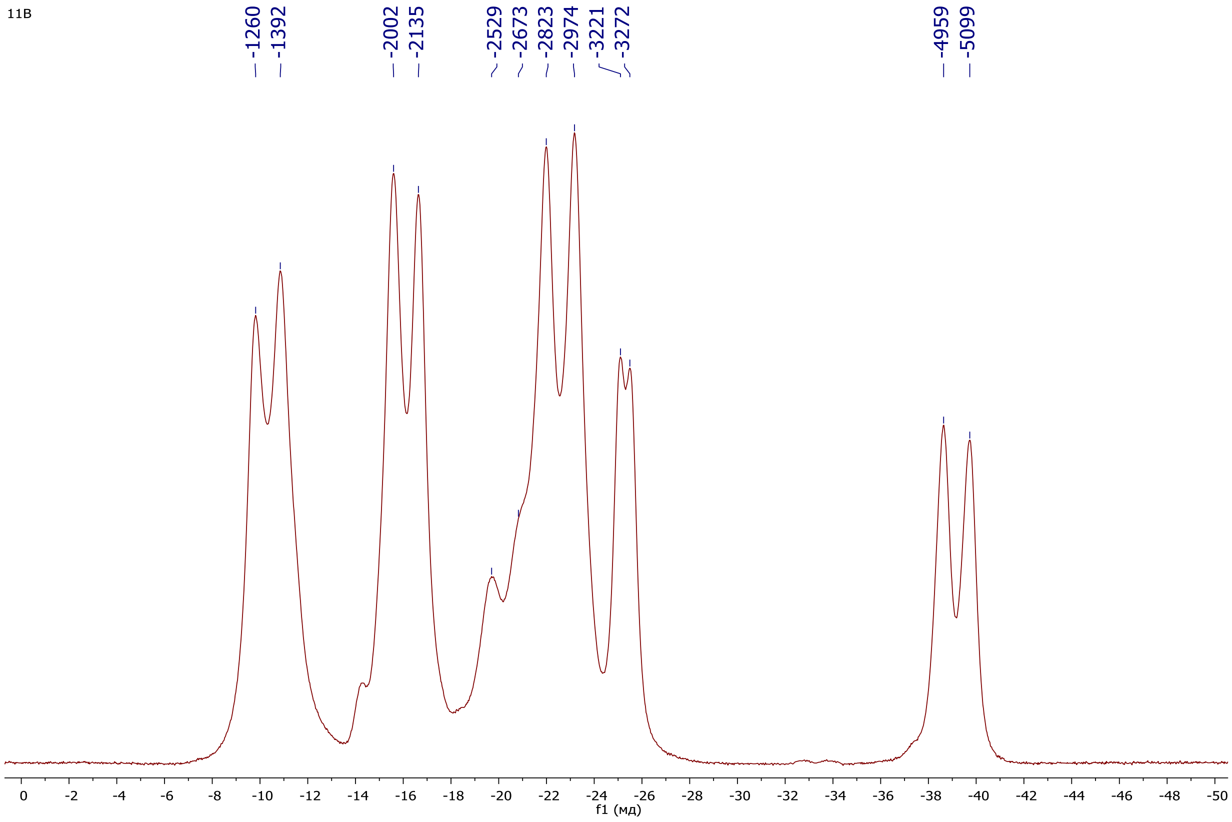

Fig. S4. ^{11}B NMR spectrum of compound **2**.

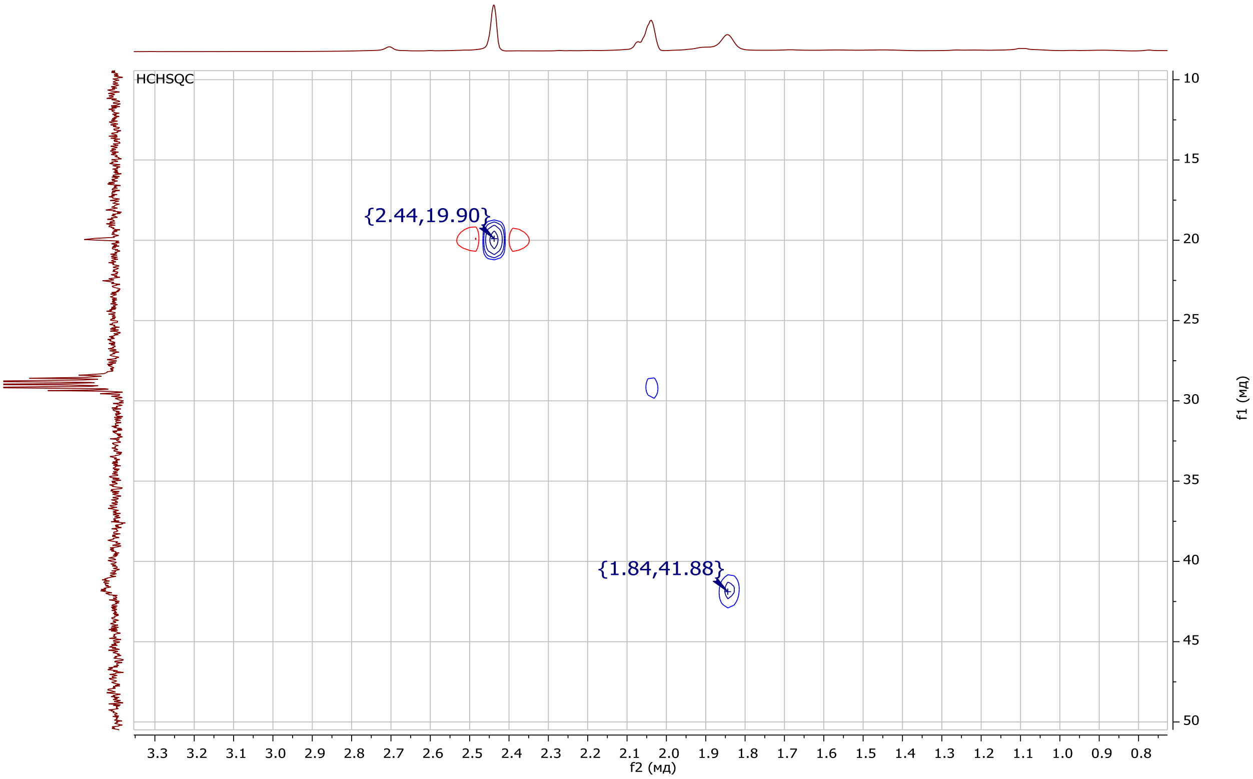

Fig. S5. ^{1}H-^{13}C NMR HMQC-spectrum of compound **2**.

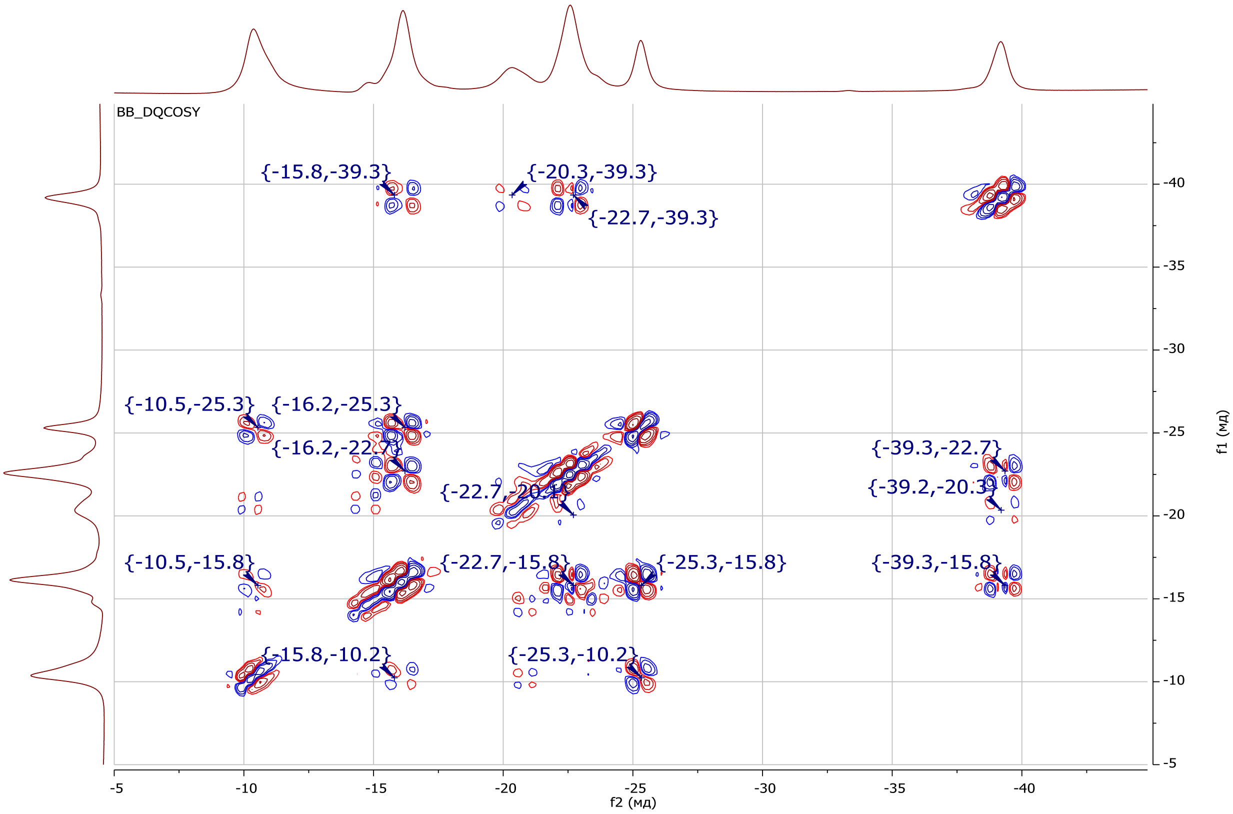

Fig. S6. ^{11}B-^{11}B NMR COSY-spectrum of compound **2**.

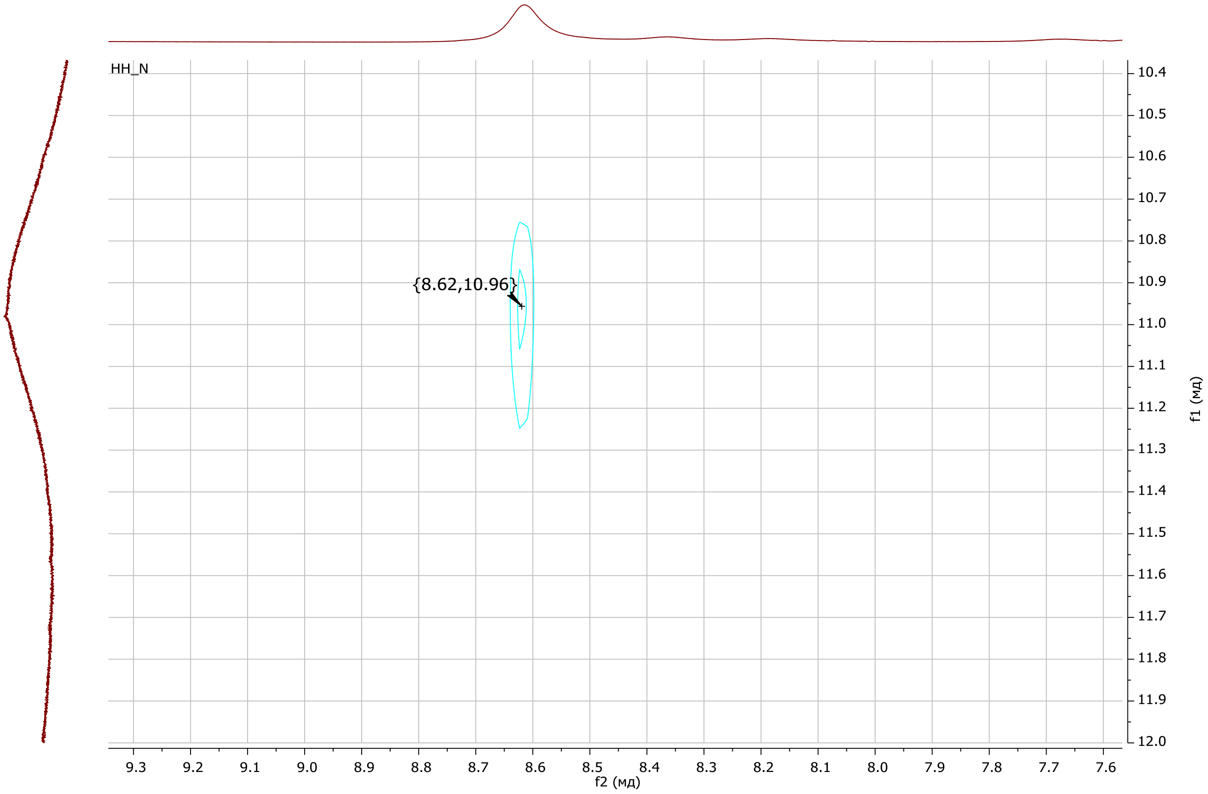

Fig. S7. ^{1}H-^{1}H NMR NOESY-spectrum of compound **2**.

$Et_3NH[10\text{-}MeC(=O)=HN\text{-}7,8\text{-}C_2B_9H_{11}]$ (3)

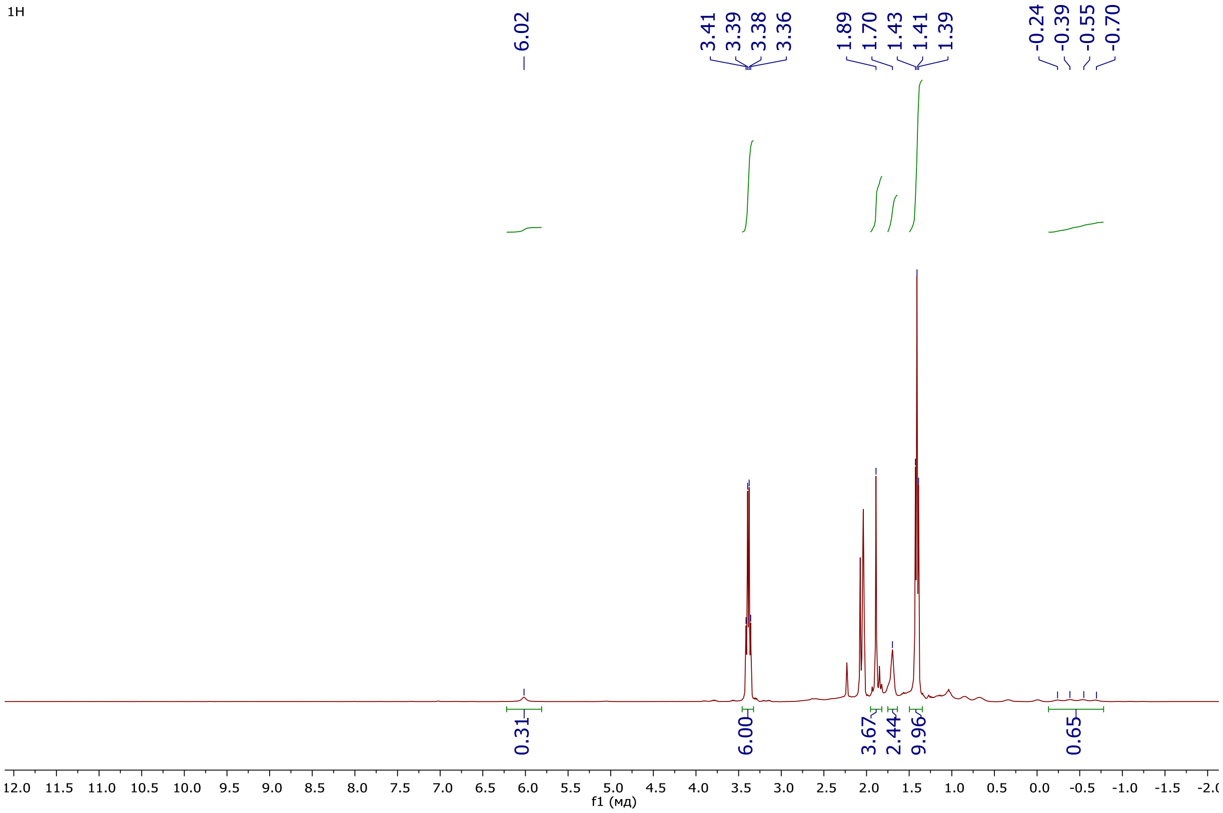

Fig. S8. ^{1}H NMR spectrum of compound **3**.

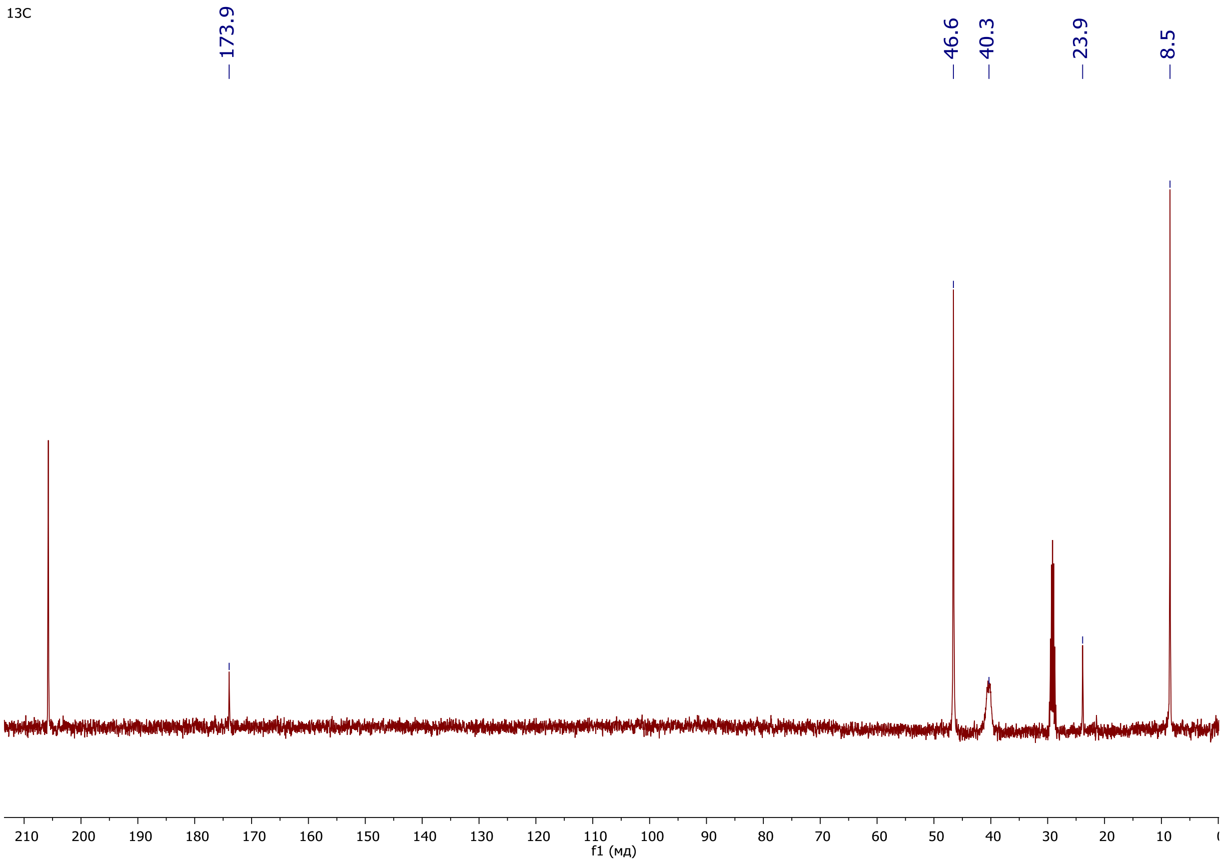

Fig. S9. ^{13}C NMR spectrum of compound **3**.

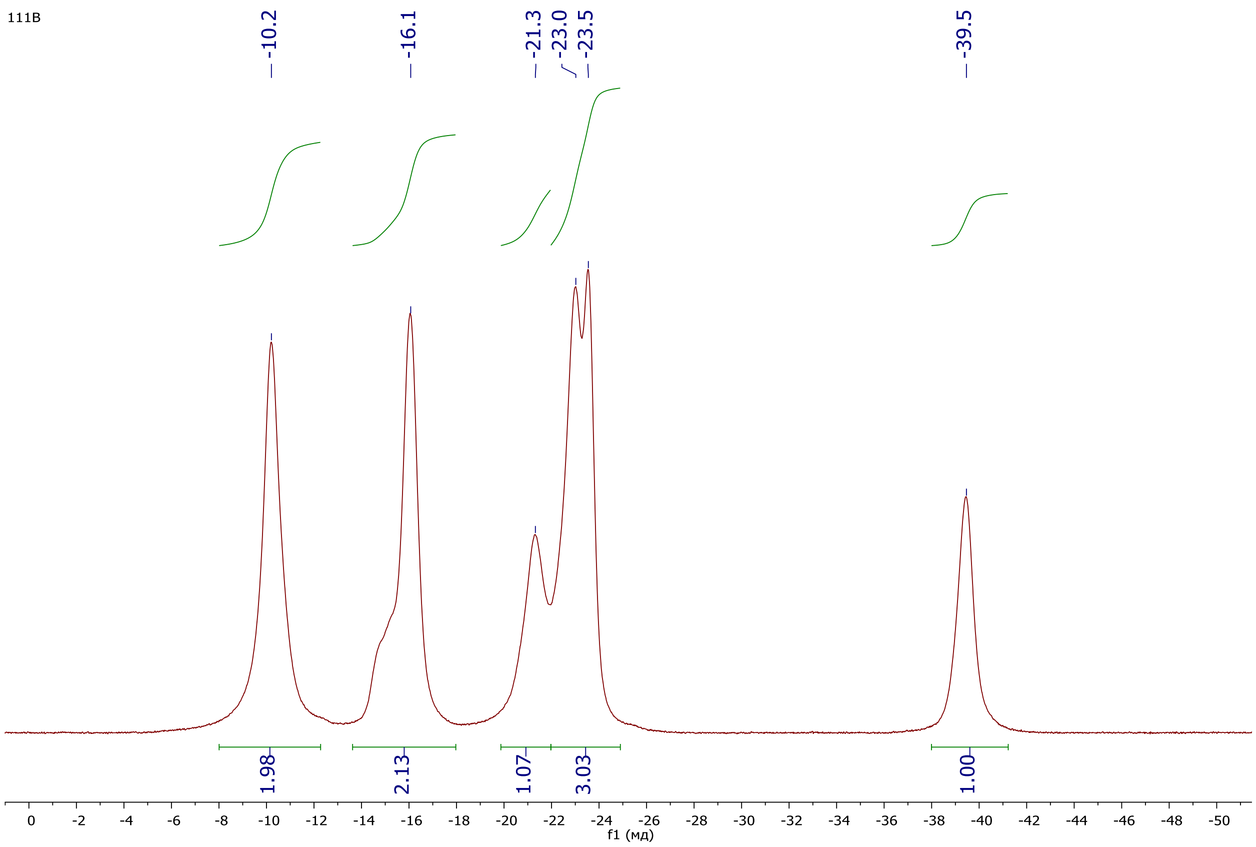

Fig. S10. $^{11}B\{^{1}H\}$ NMR spectrum of compound **3**.

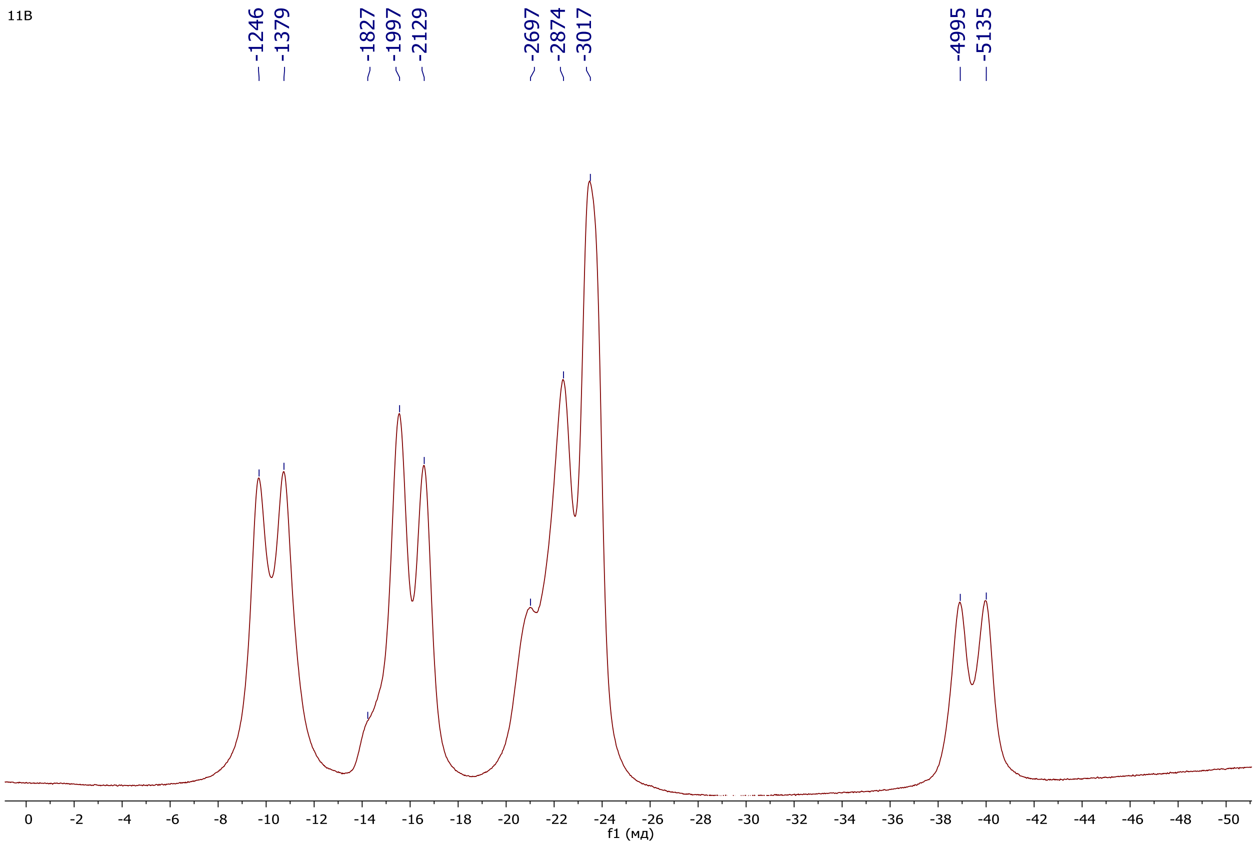

Fig. S11. ^{11}B NMR spectrum of compound **3**.

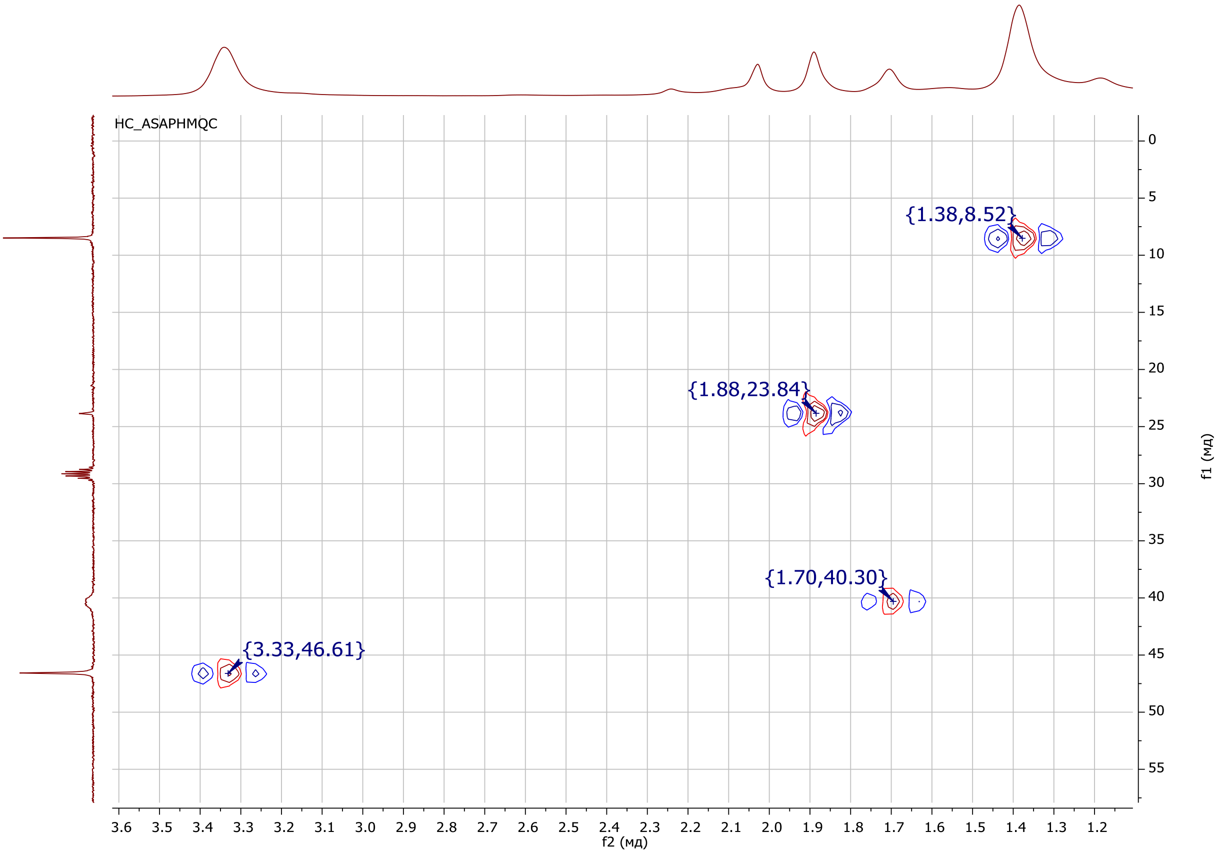

Fig. S12. ^{1}H-^{13}C NMR HMQC-spectrum of compound **3**.

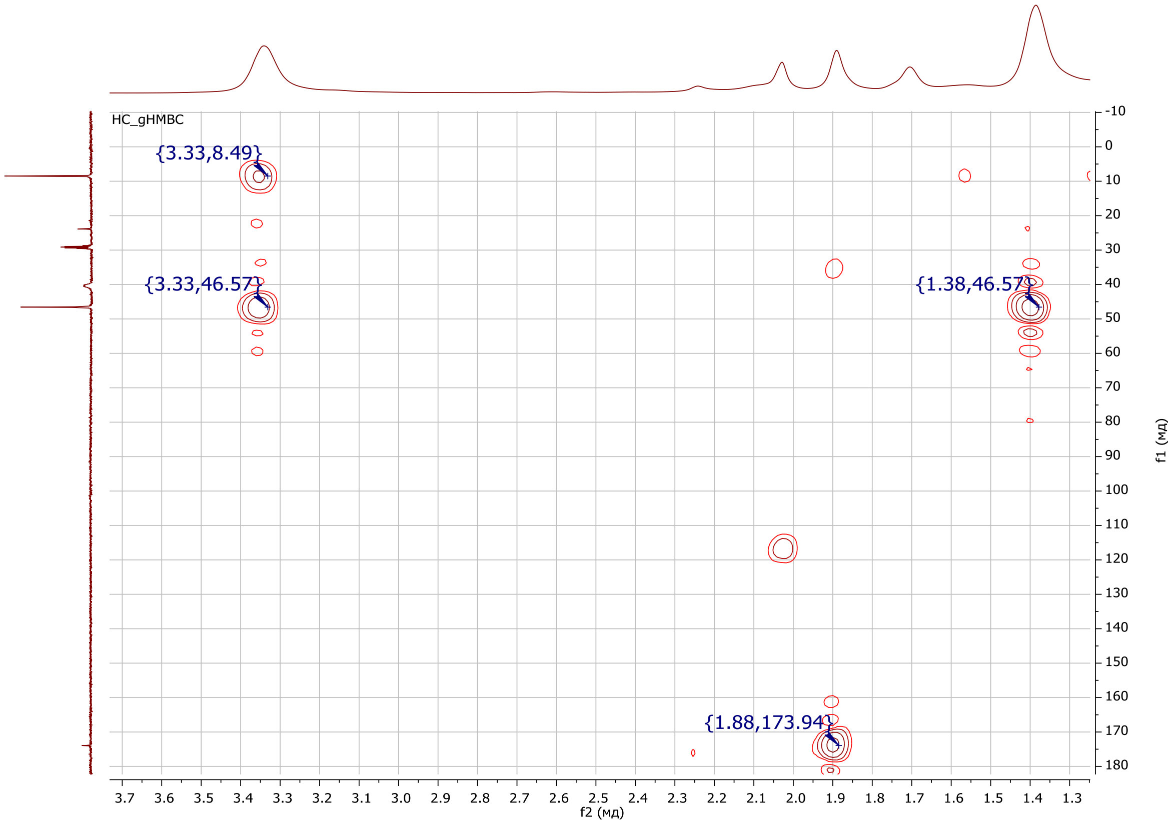

Fig. S13. ^{1}H-^{13}C NMR HMBC-spectrum of compound **3**.

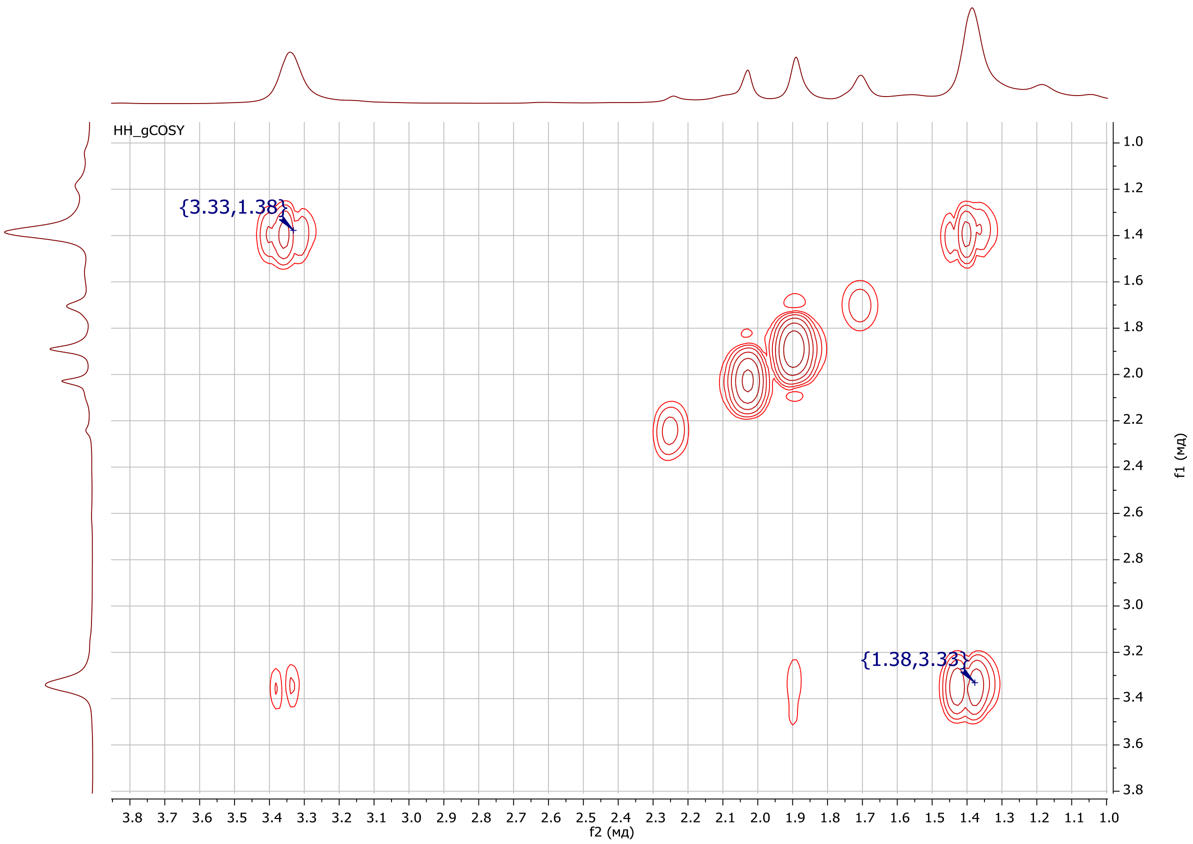

Fig. S14. ^{1}H-^{1}H NMR COSY-spectrum of compound **3**.

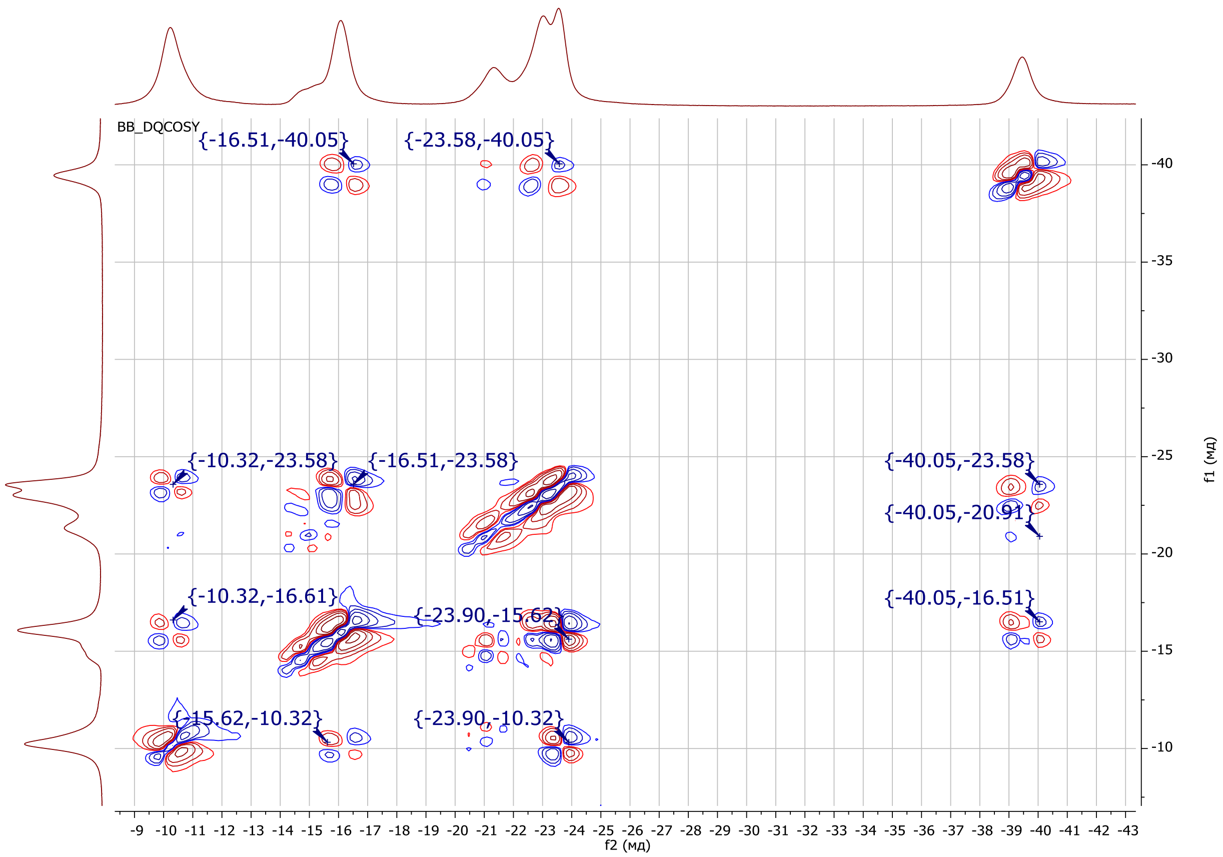

Fig. S15. ^{11}B-^{11}B NMR COSY-spectrum of compound **3**.

10-MeC(OMe)=HN-7,8-$C_2B_9H_{11}$ (4)

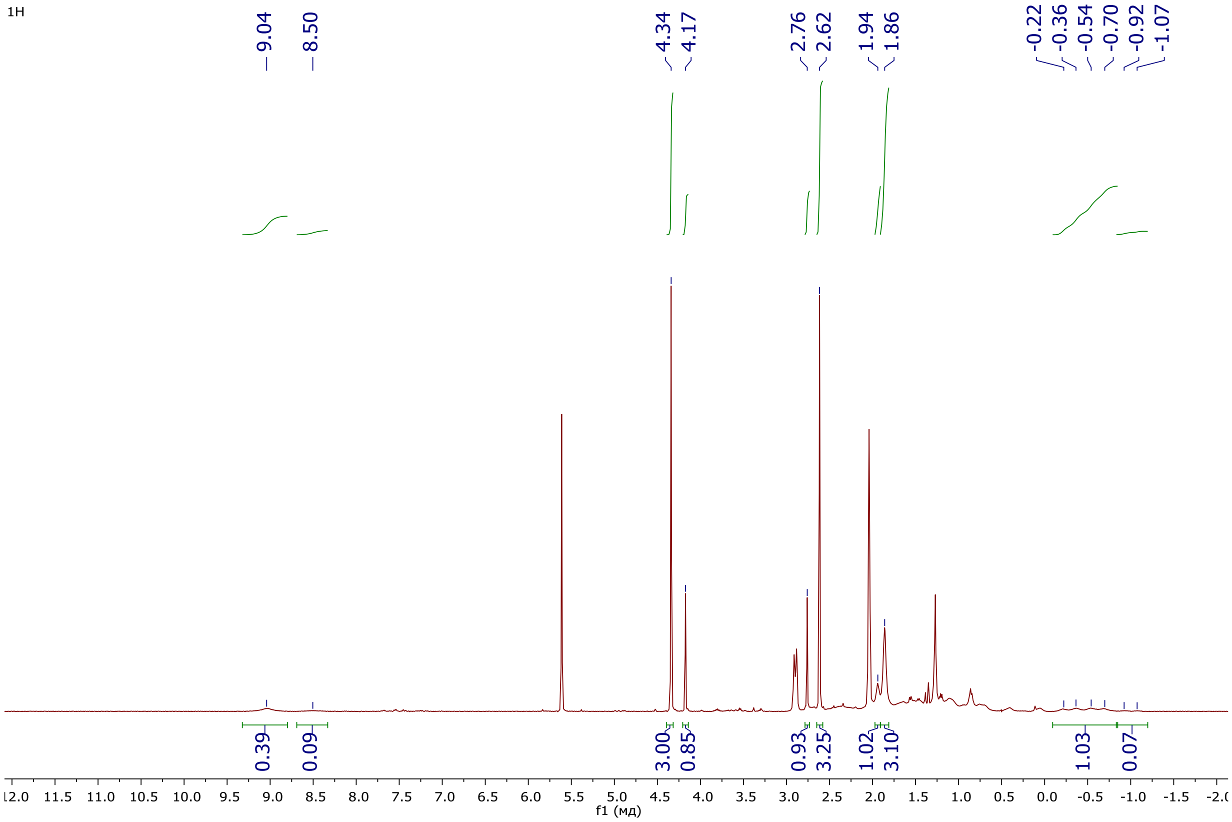

Fig. S16. ^{1}H NMR spectrum of compounds **4**.

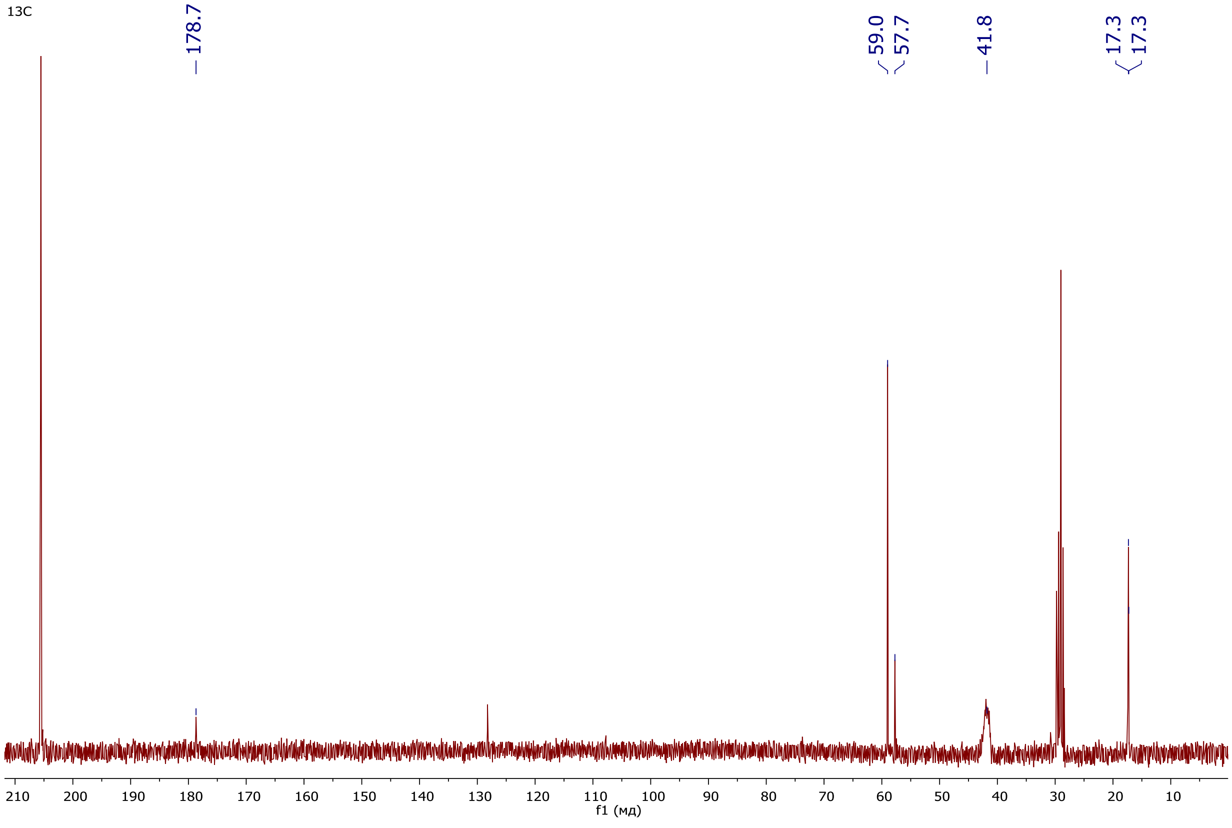

Fig. S17. ^{13}C NMR spectrum of compounds **4**.

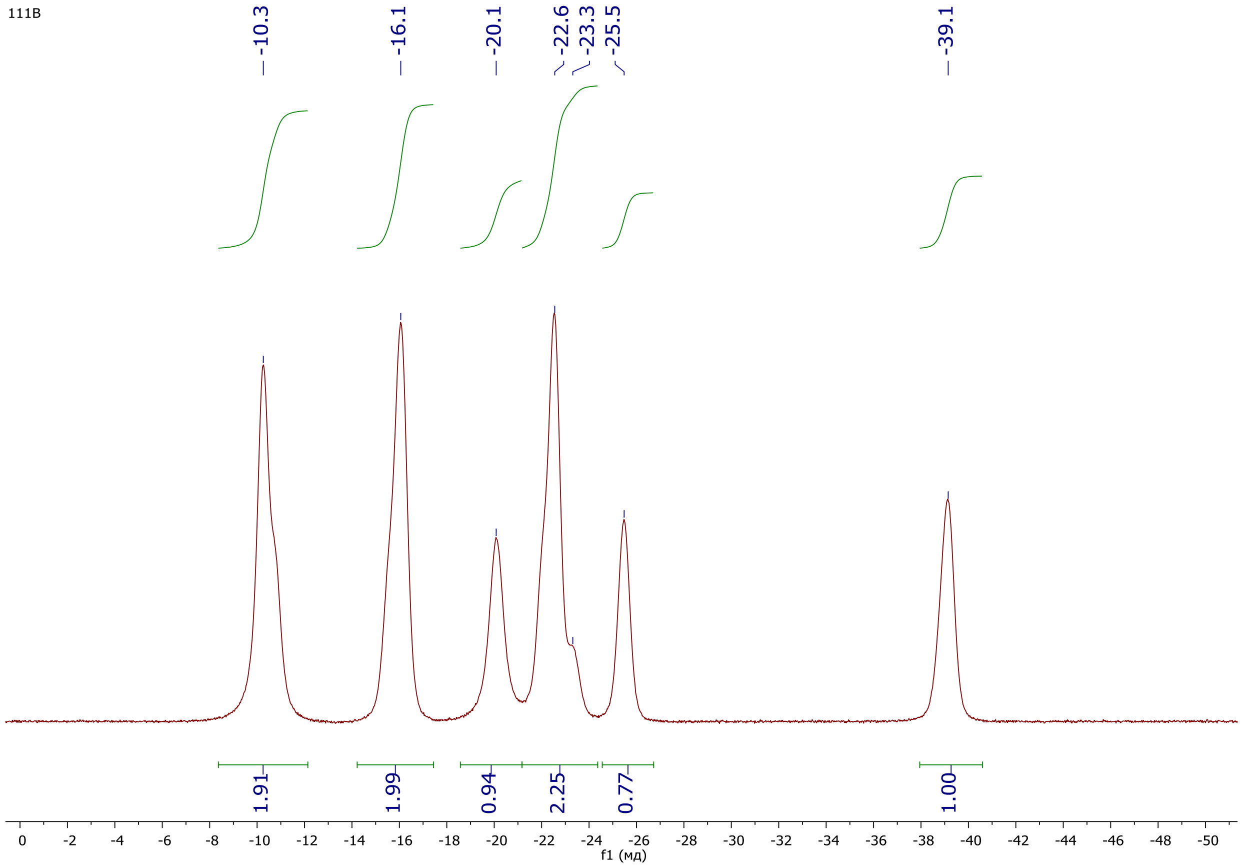

Fig. S18. $^{11}B\{^{1}H\}$ NMR spectrum of compounds **4**.

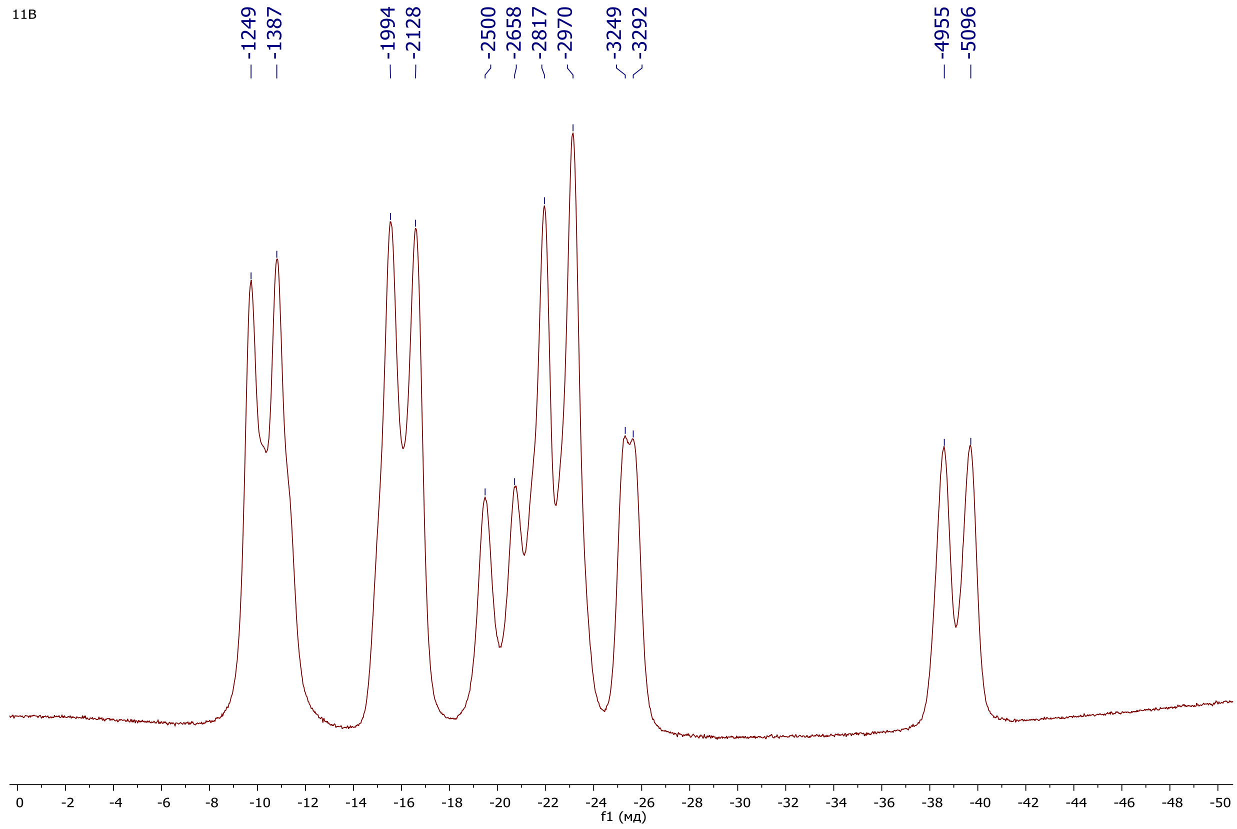

Fig. S19. ^{11}B NMR spectrum of compounds **4**.

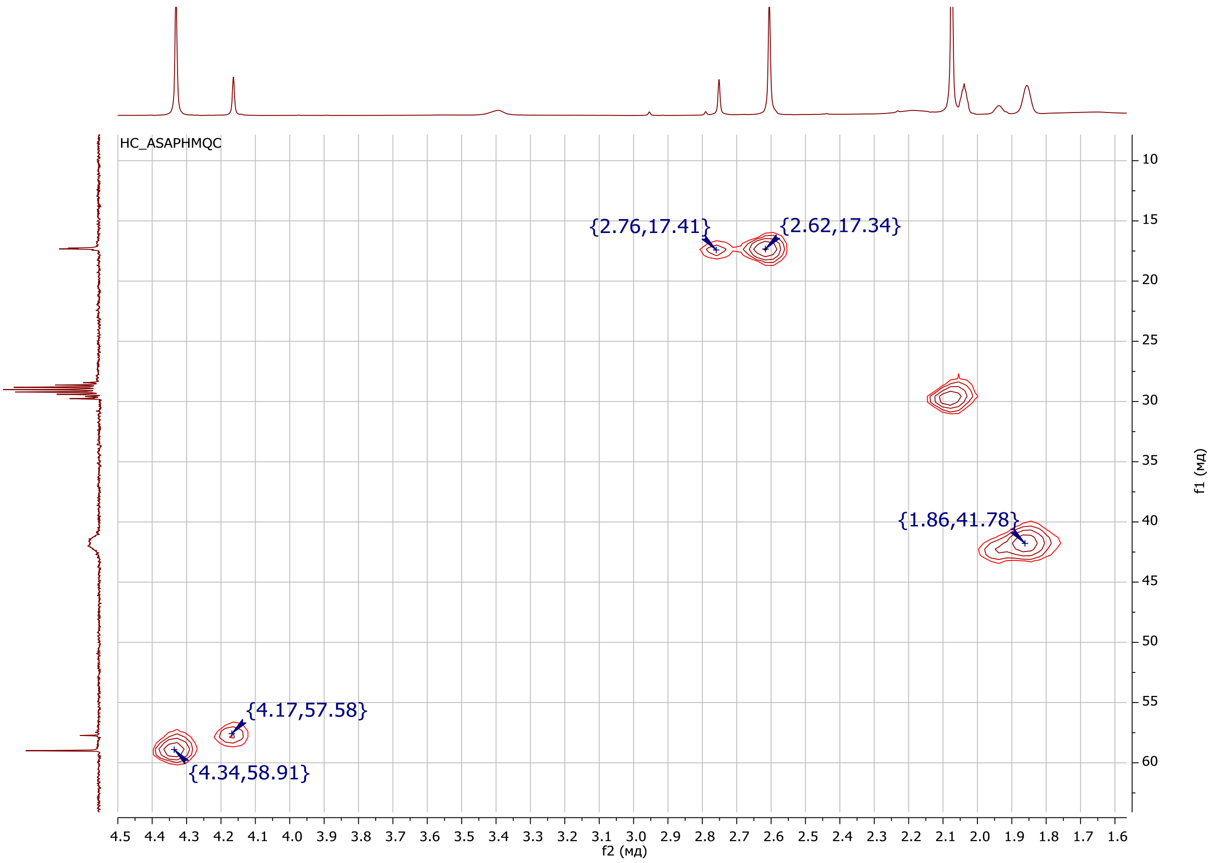

Fig. S20. ^{1}H-^{13}C NMR HMQC-spectrum of compounds **4**.

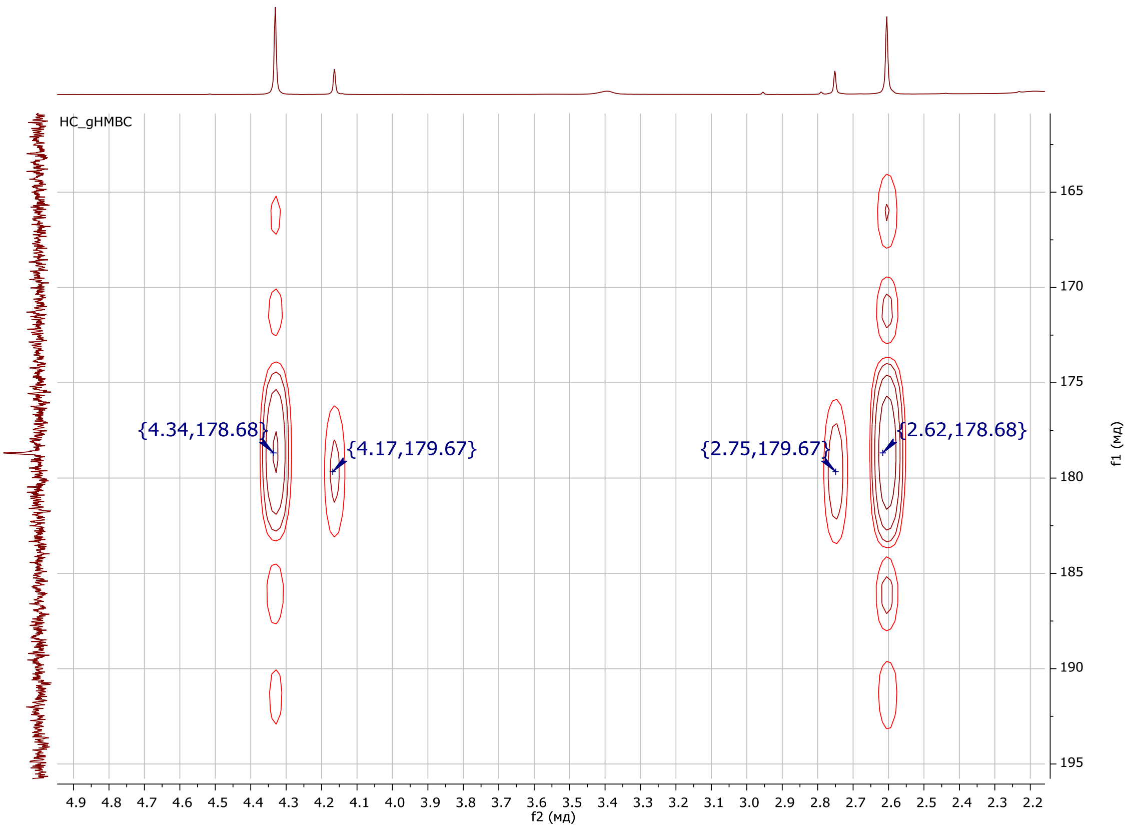

Fig. S21. ^{1}H-^{13}C NMR HMBC-spectrum of compounds **4**.

10-MeC(OEt)=HN-7,8-$C_2B_9H_{11}$ (5)

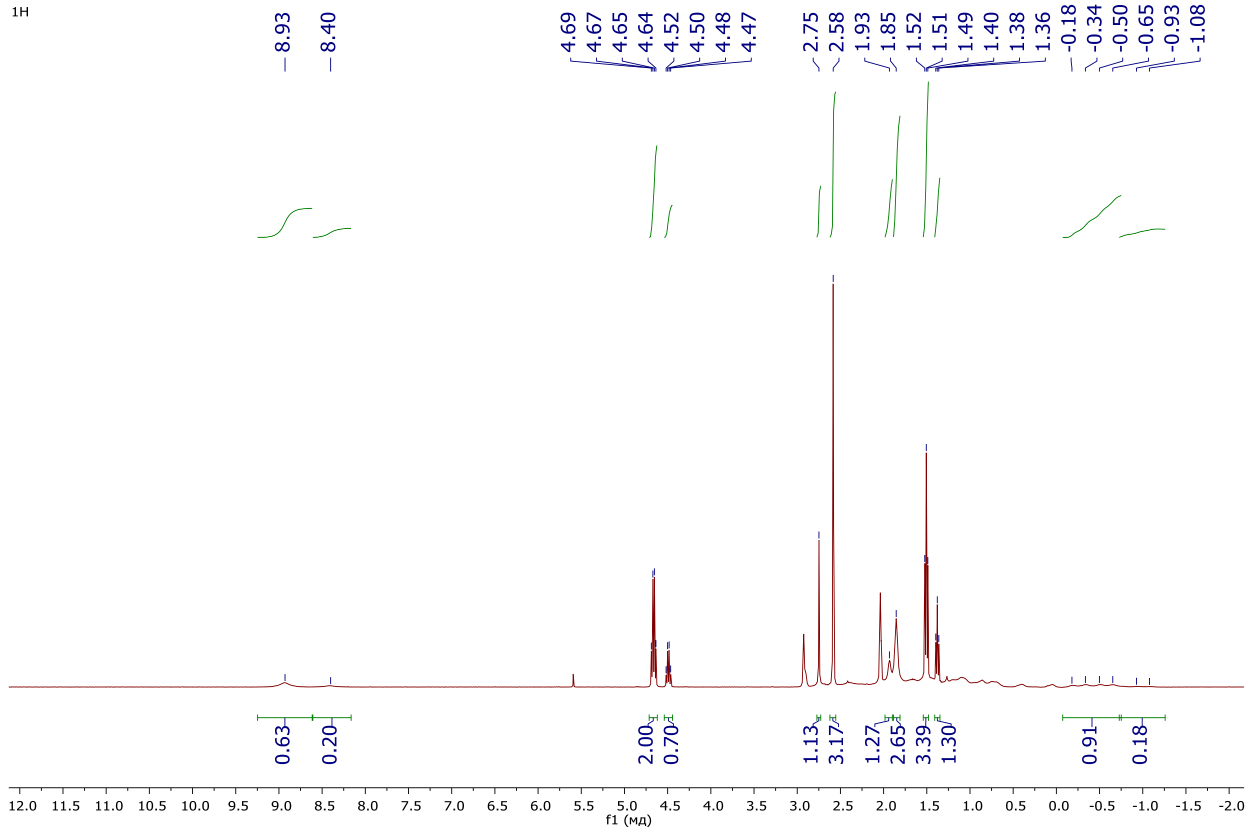

Fig. S22. ^{1}H NMR spectrum of compounds **5**.

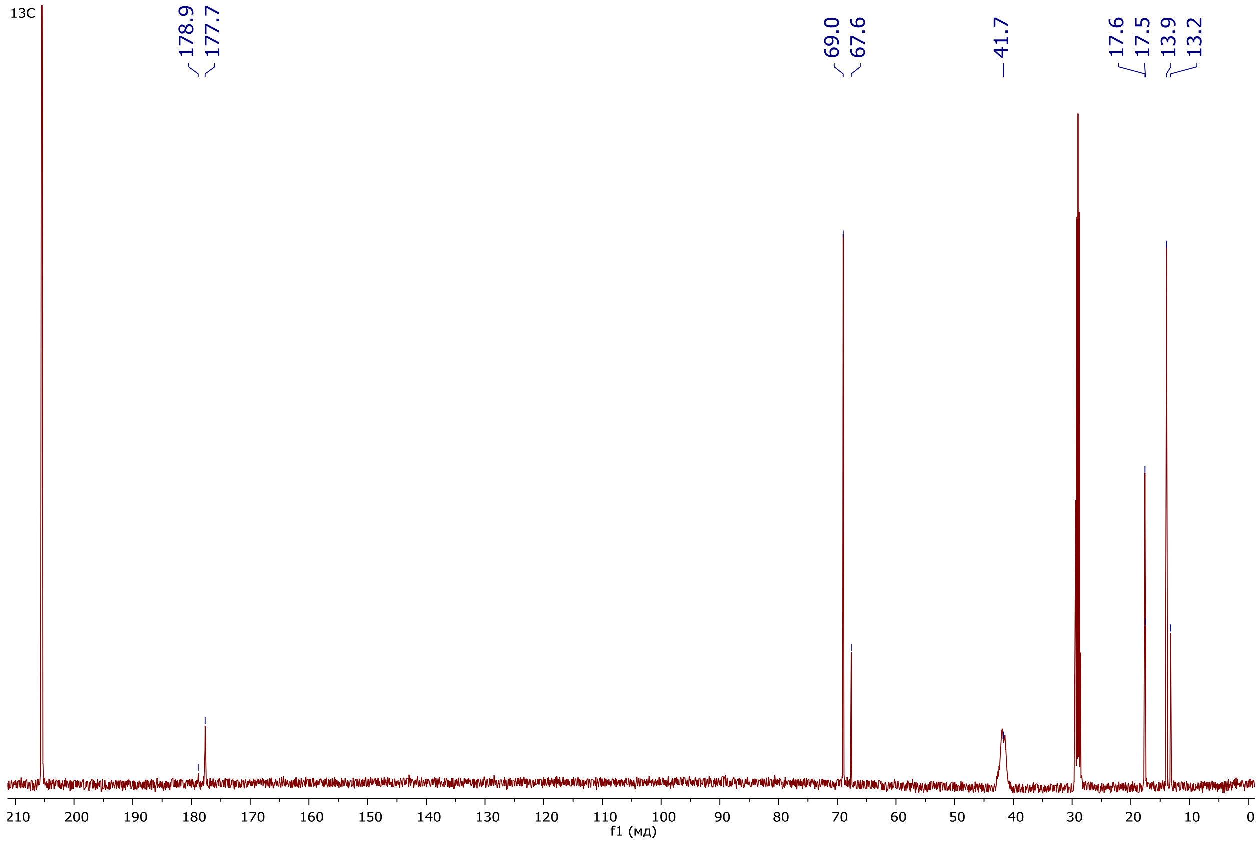

Fig. S23. ^{13}C NMR spectrum of compounds **5**.

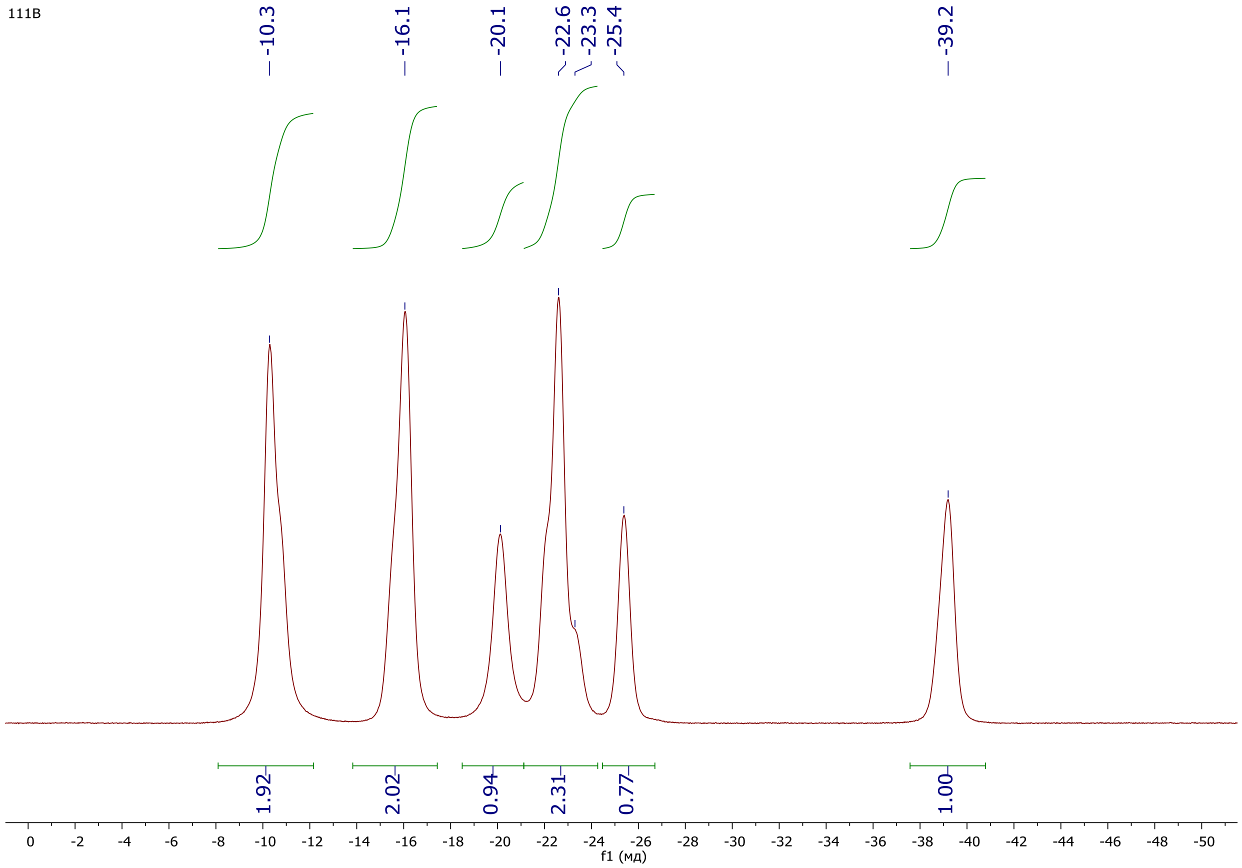

Fig. S24. $^{11}B\{^{1}H\}$ NMR spectrum of compounds **5**.

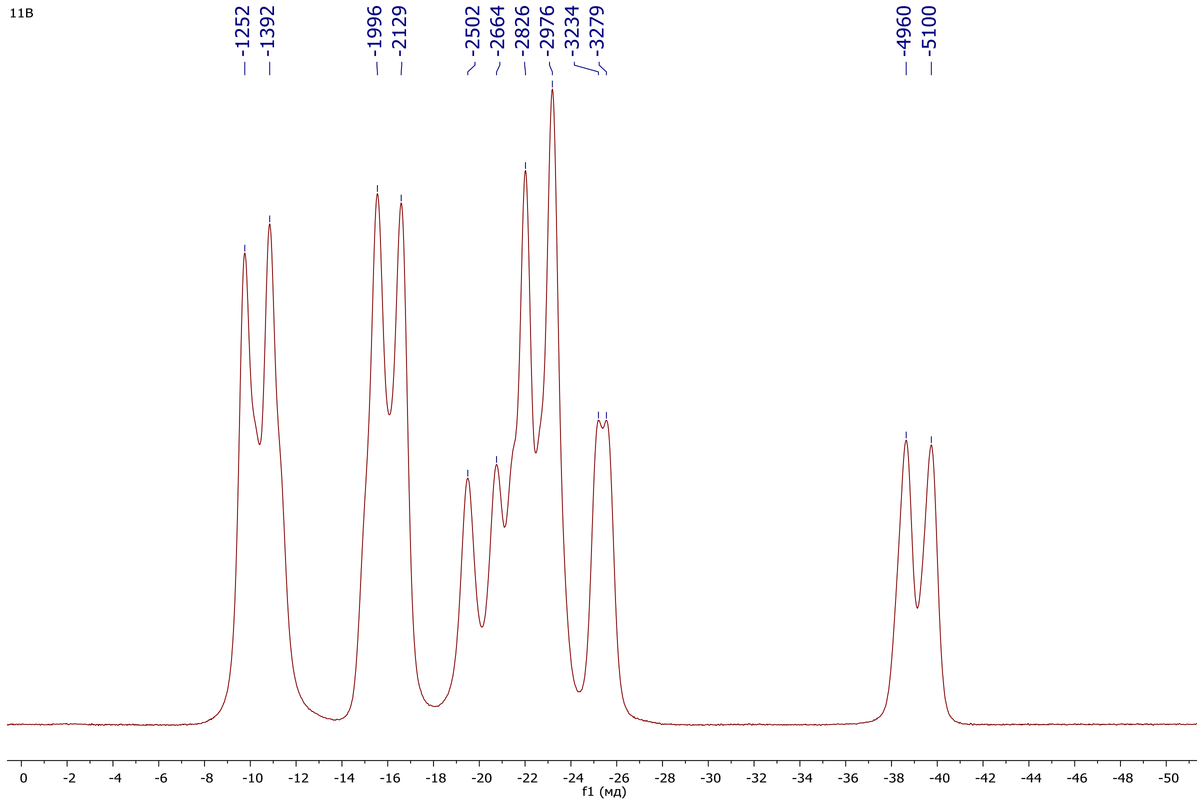

Fig. S25. ^{11}B NMR spectrum of compounds **5**.

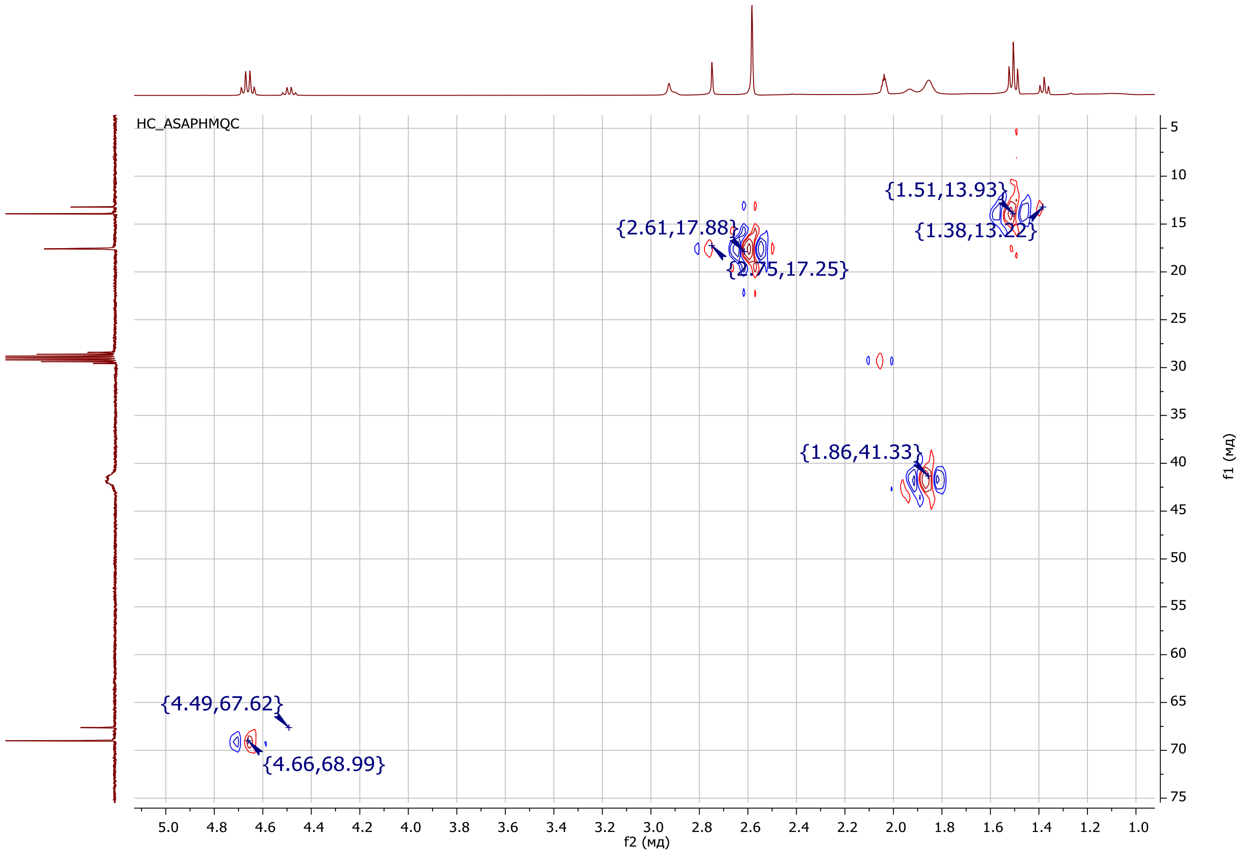

Fig. S26. ^{1}H-^{13}C NMR HMQC-spectrum of compounds **5**.

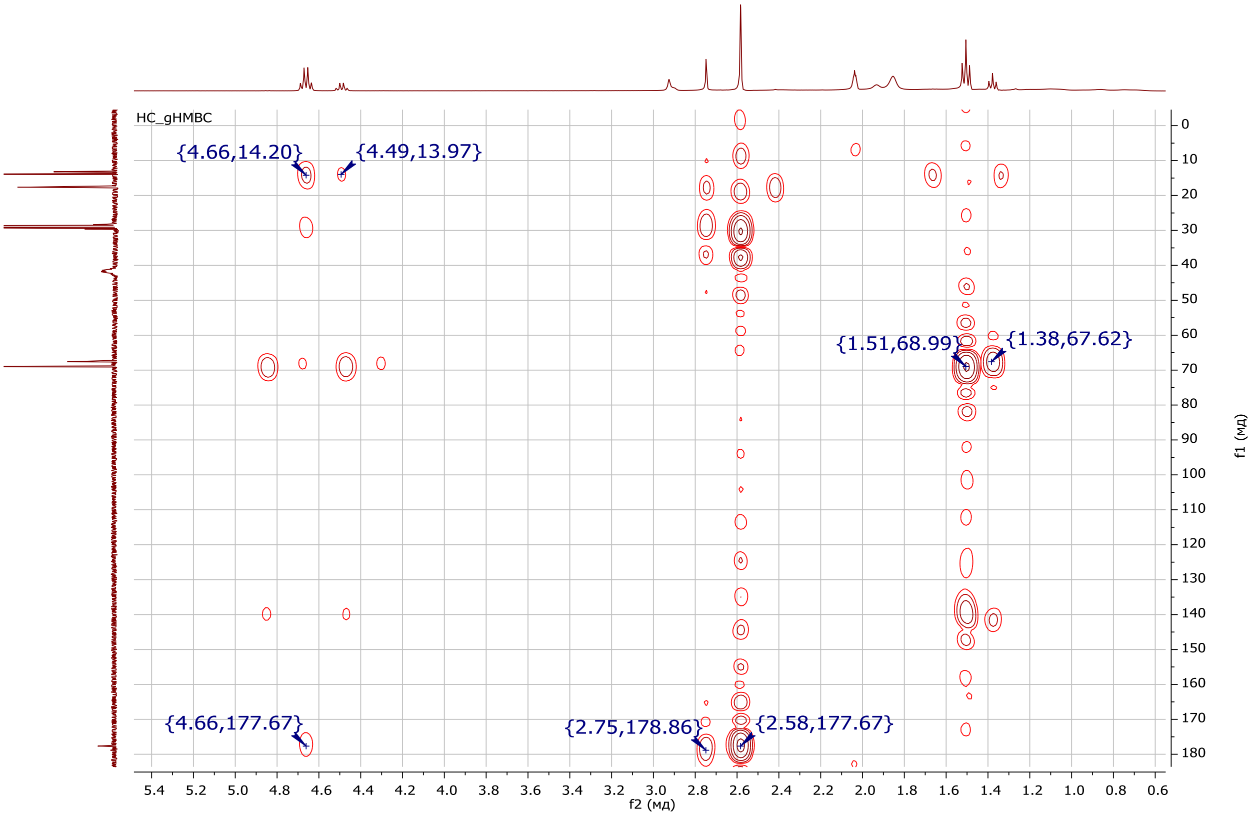

Fig. S27. ^{1}H-^{13}C NMR HMBC-spectrum of compounds **5**.

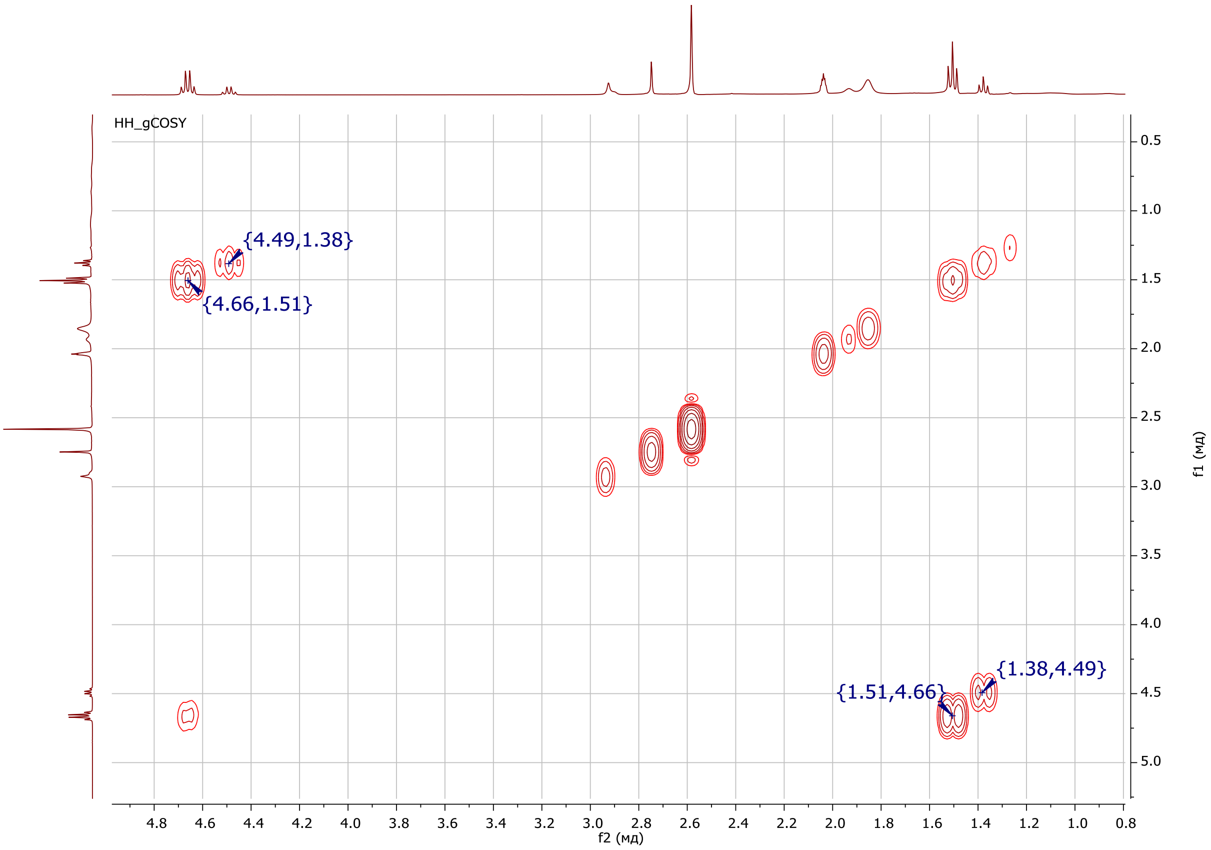

Fig. S28. ^{1}H-^{1}H NMR COSY-spectrum of compounds **5**.

10-MeC(NHMe)=HN-7,8-$C_2B_9H_{11}$ (6)

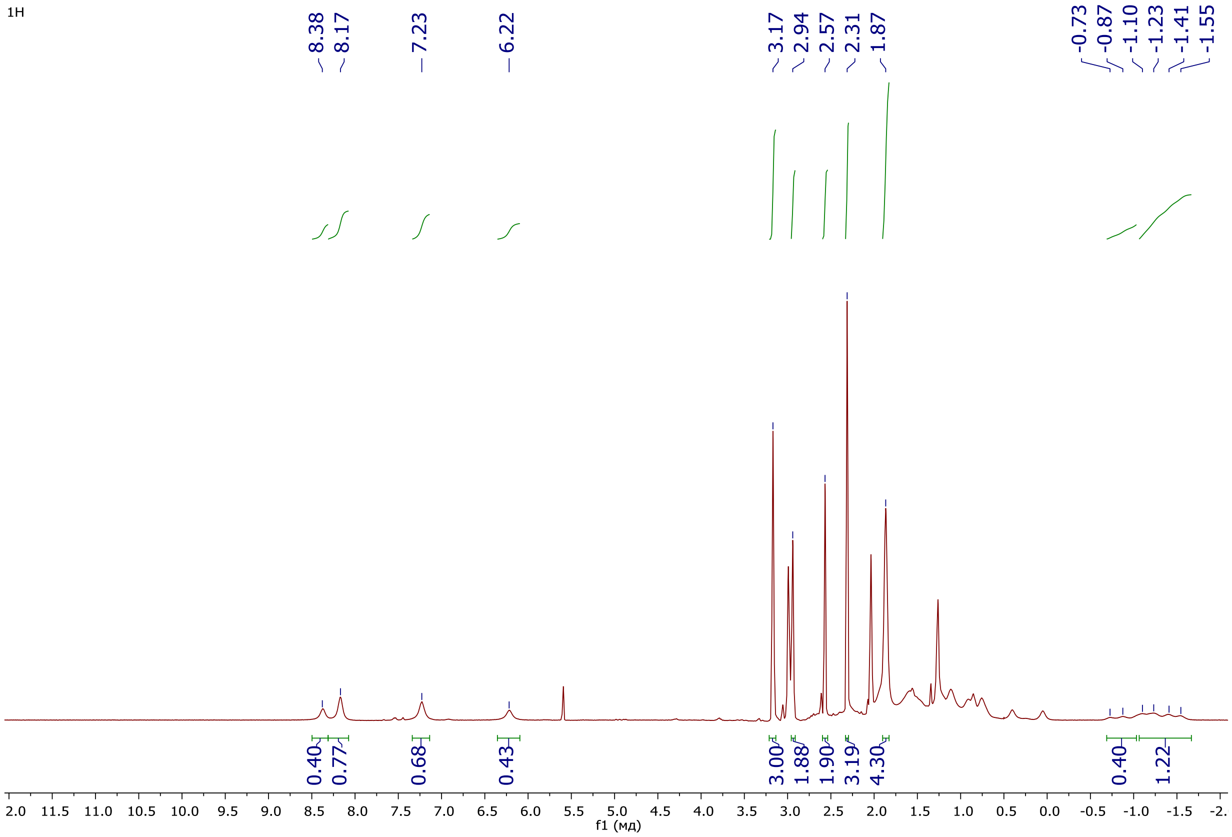

Fig. S29. ^{1}H NMR spectrum of compounds **6**.

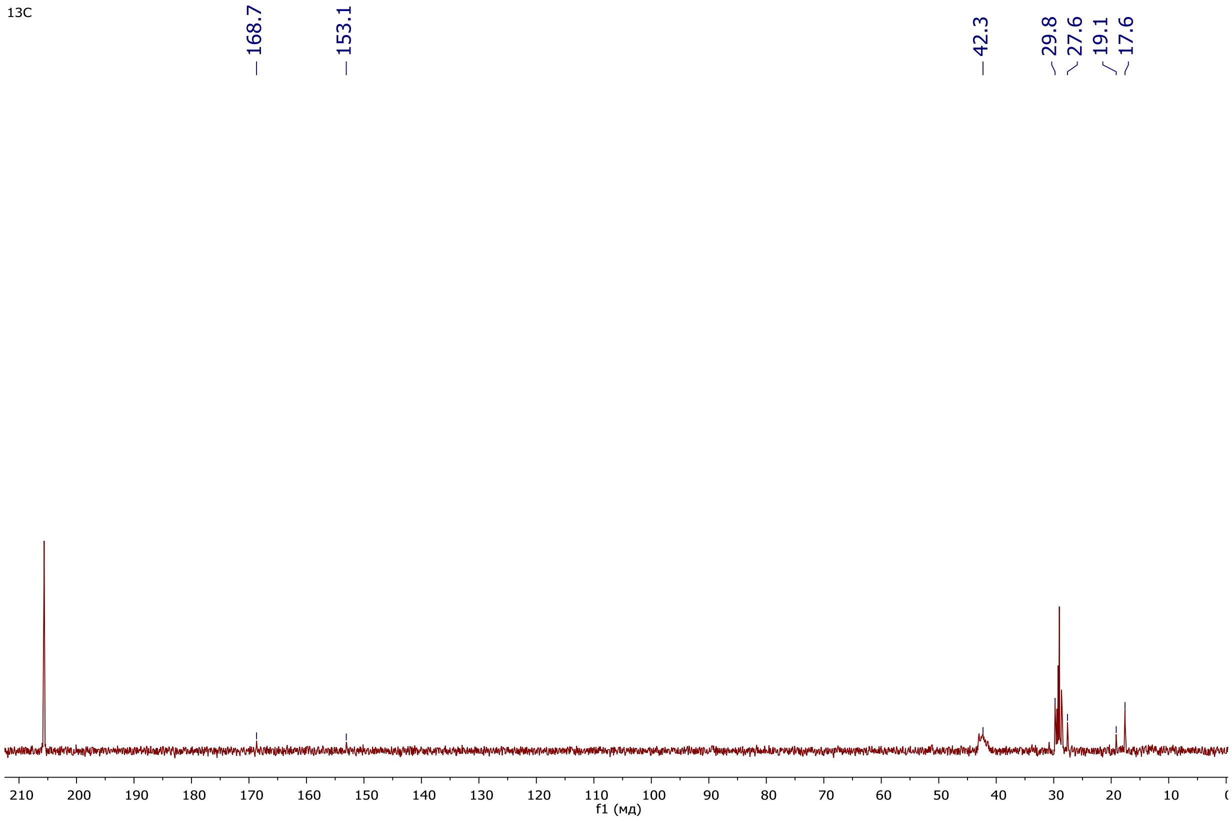

Fig. S30. ^{13}C NMR spectrum of compounds **6**.

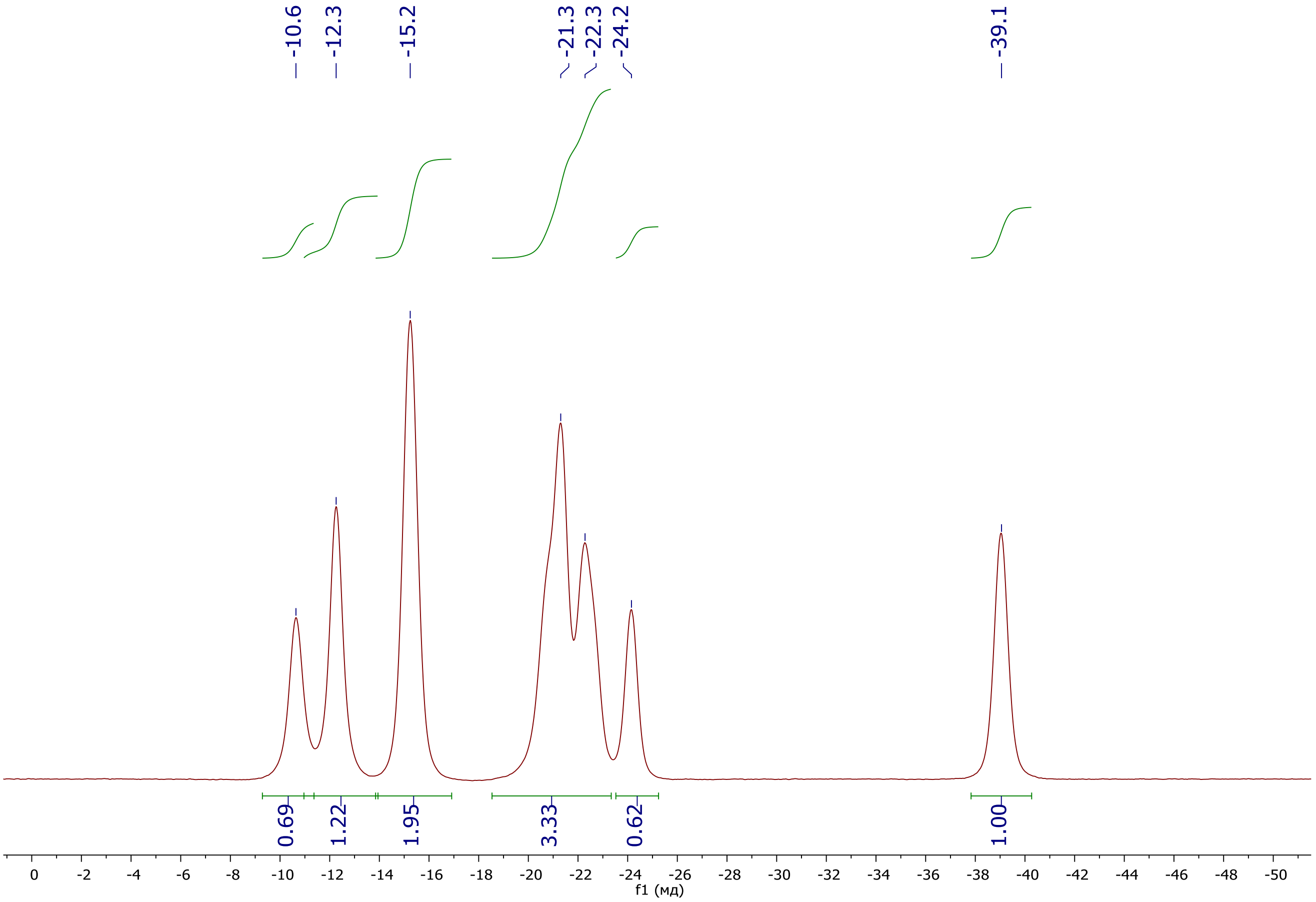

Fig. S31. $^{11}B\{^{1}H\}$ NMR spectrum of compounds **6**.

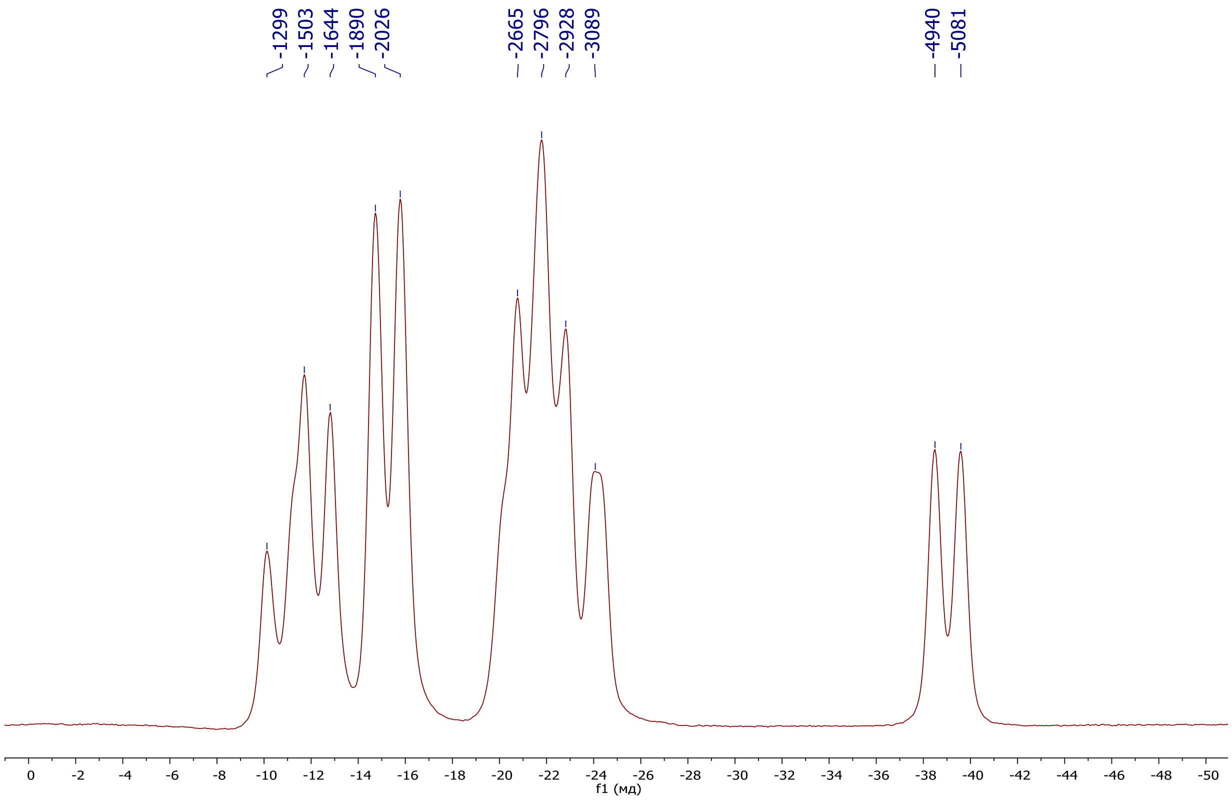

Fig. S32. ^{11}B NMR spectrum of compounds **6**.

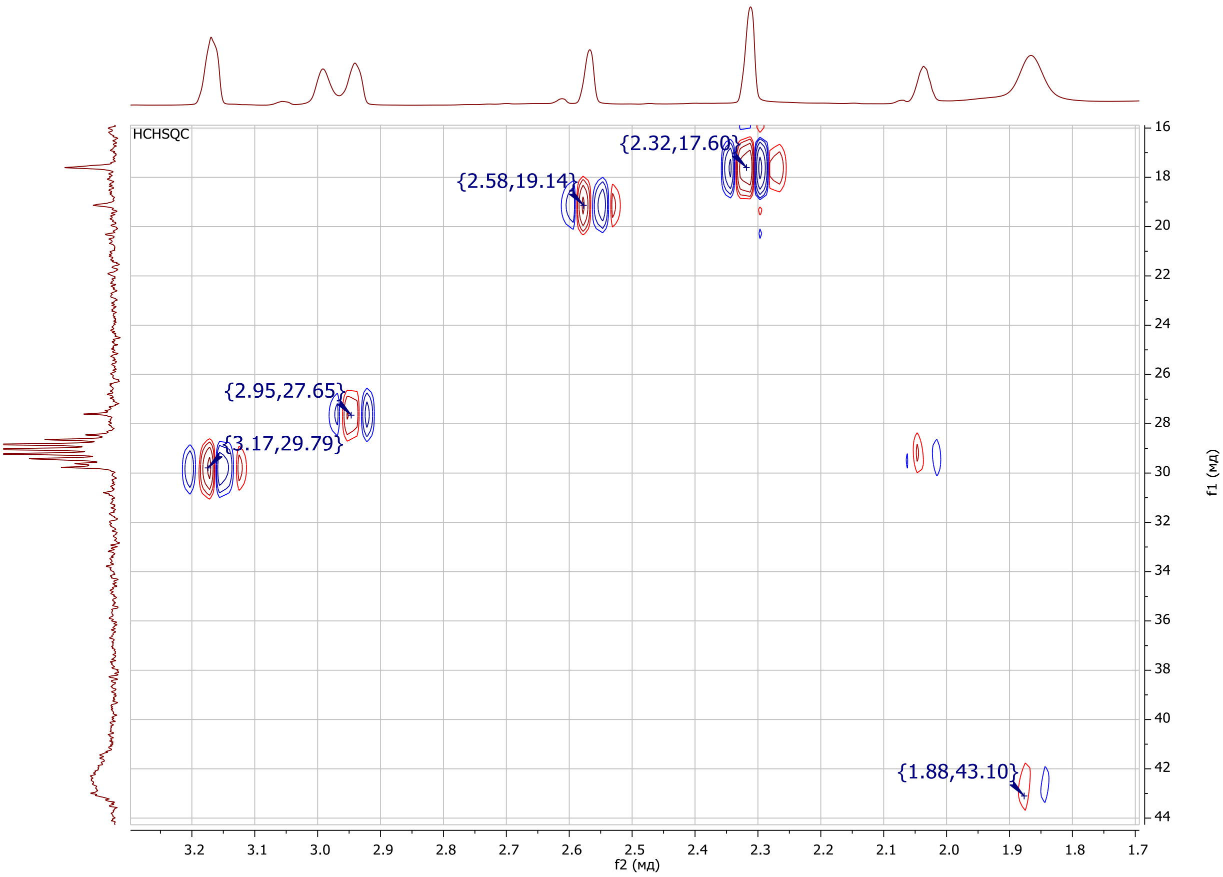

Fig. S33. ^{1}H-^{13}C NMR HSQC-spectrum of compounds **6**.

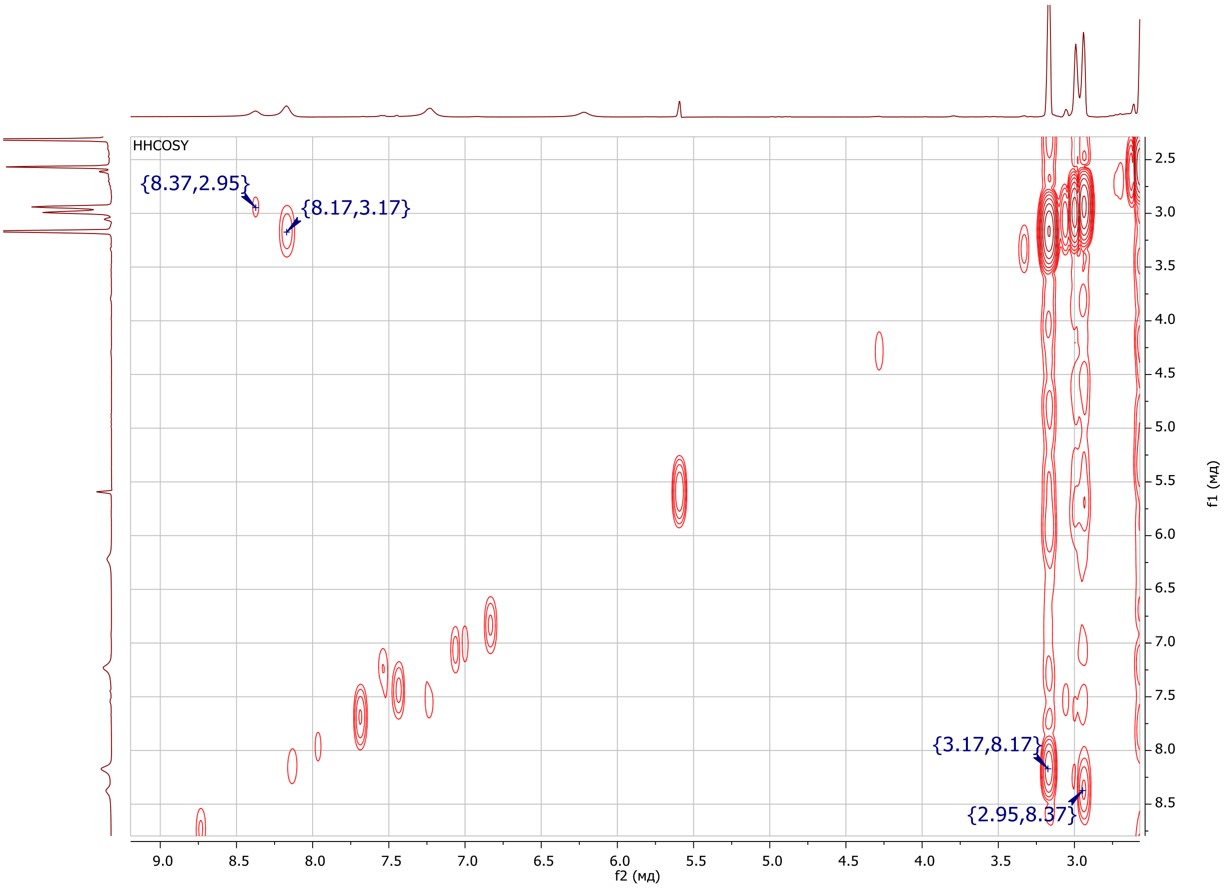

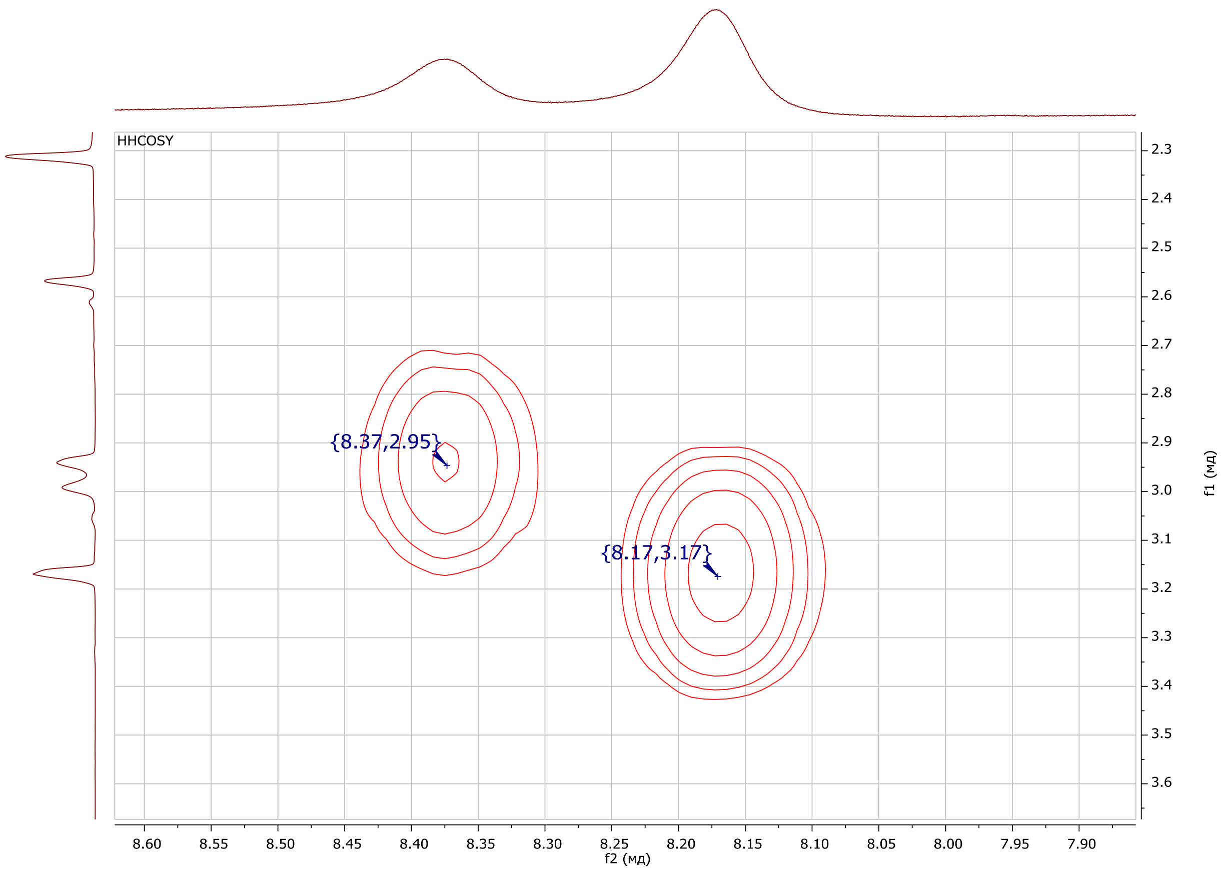

Fig. S34. ^{1}H-^{1}H NMR COSY-spectrum of compounds **6**.

10-EtC(NHEt)=HN-7,8-$C_2B_9H_{11}$ (7)

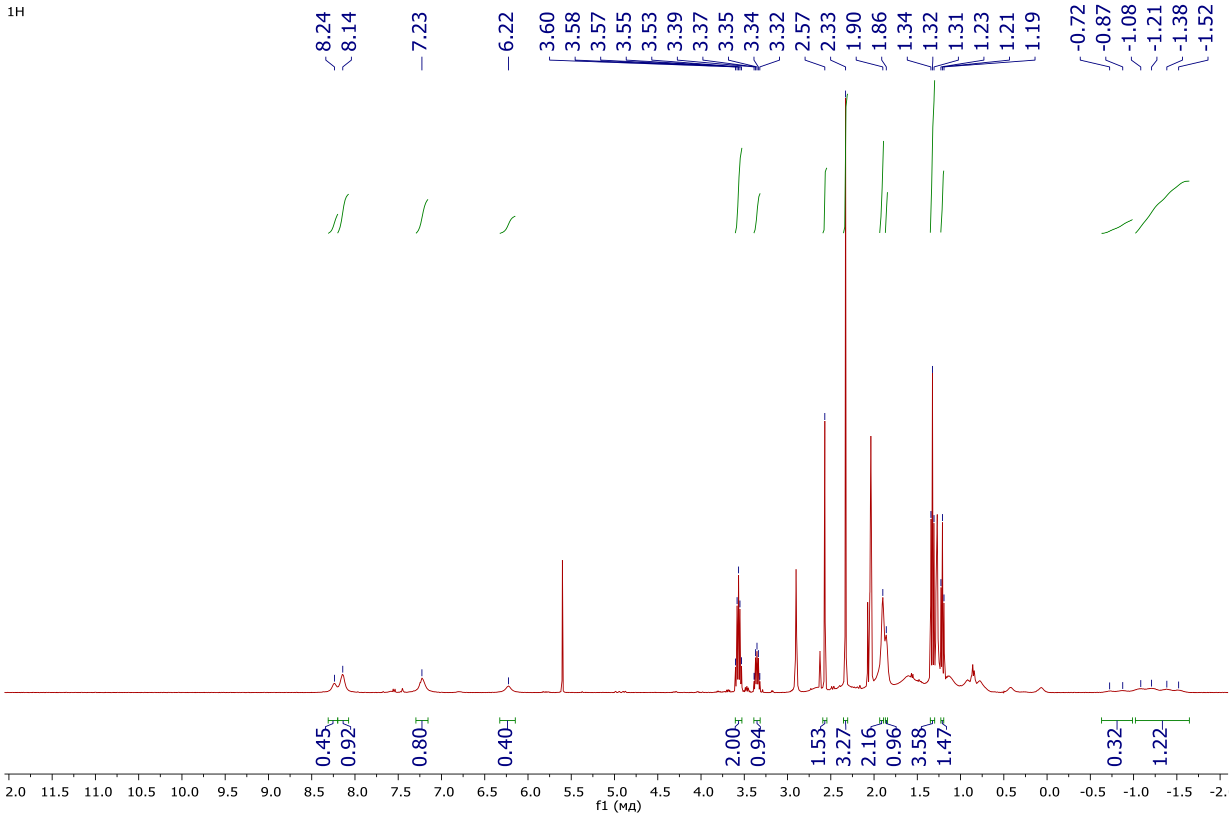

Fig. S35. ^{1}H NMR spectrum of compounds **7**.

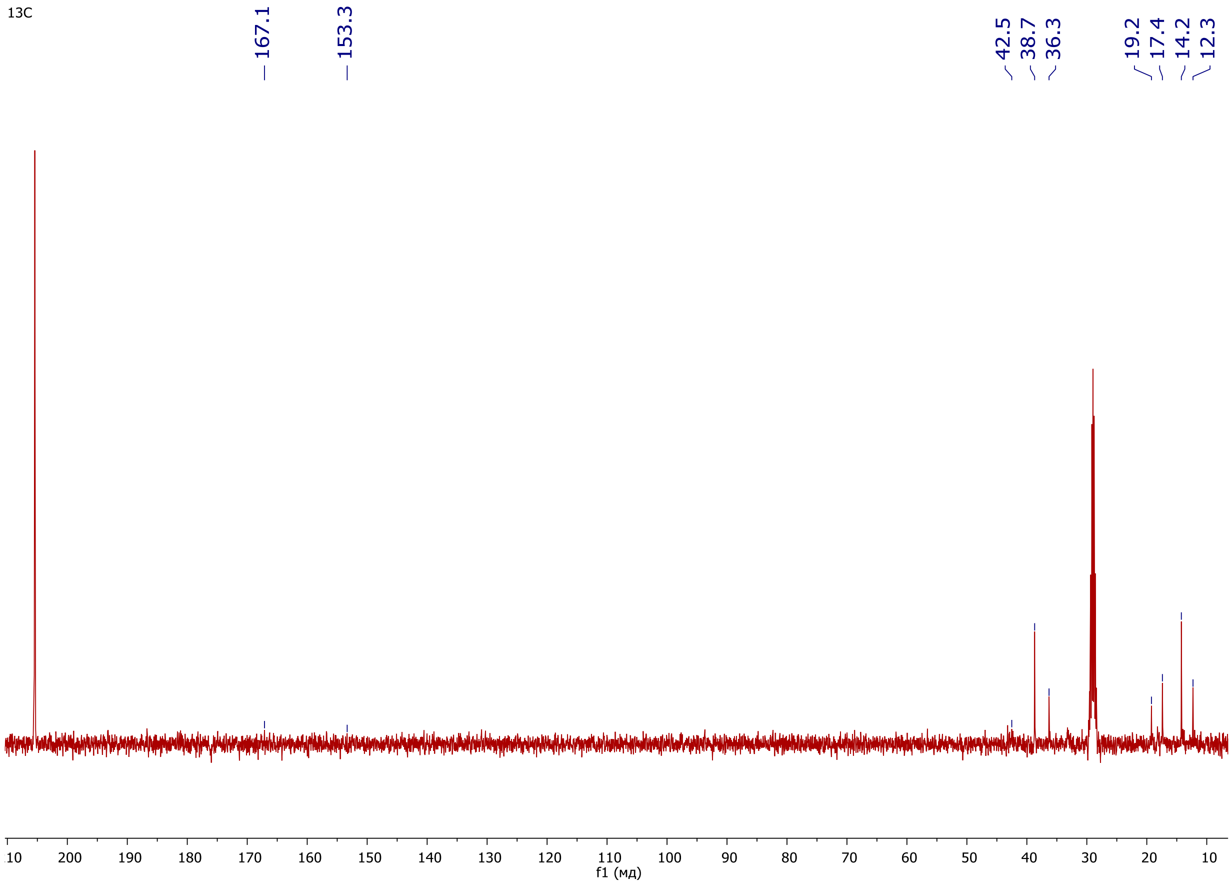

Fig. S36. ^{13}C NMR spectrum of compounds **7**.

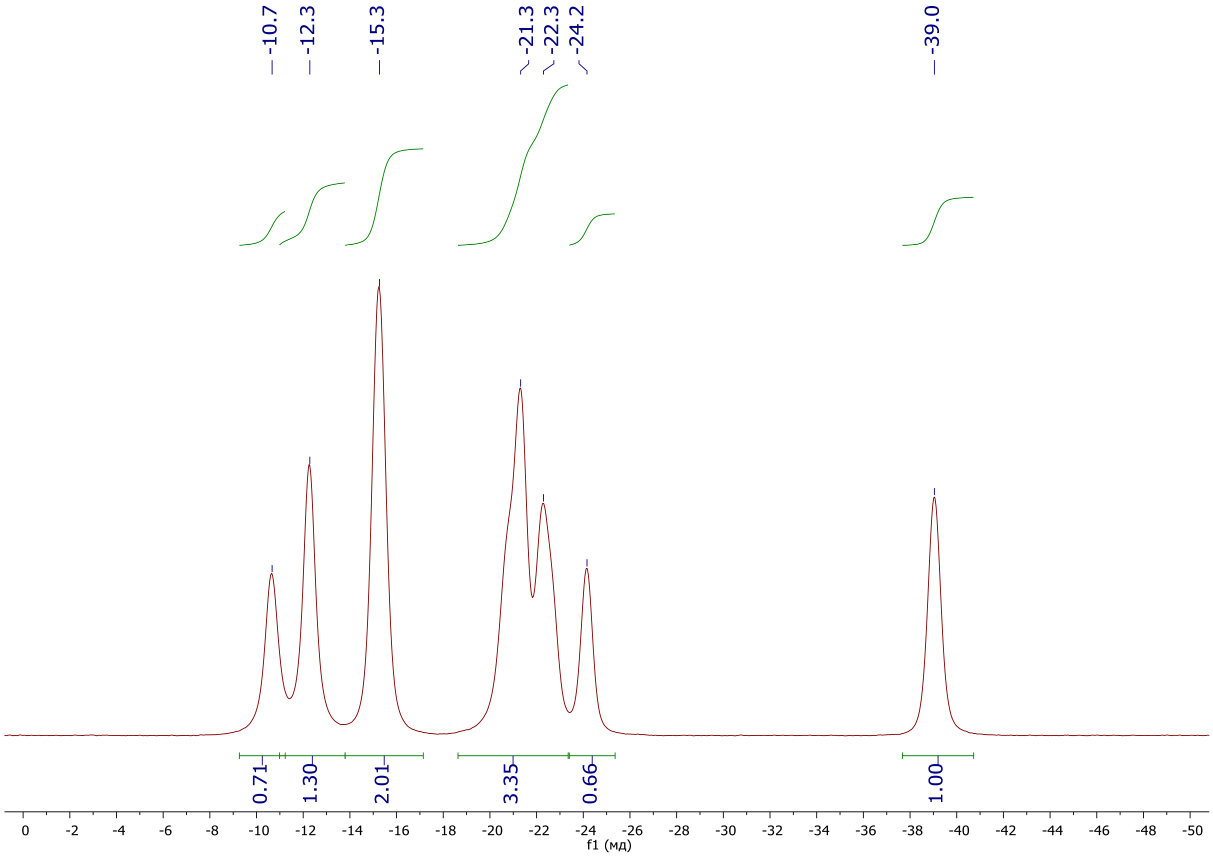

Fig. S37. $^{11}B\{^{1}H\}$ NMR spectrum of compounds **7**.

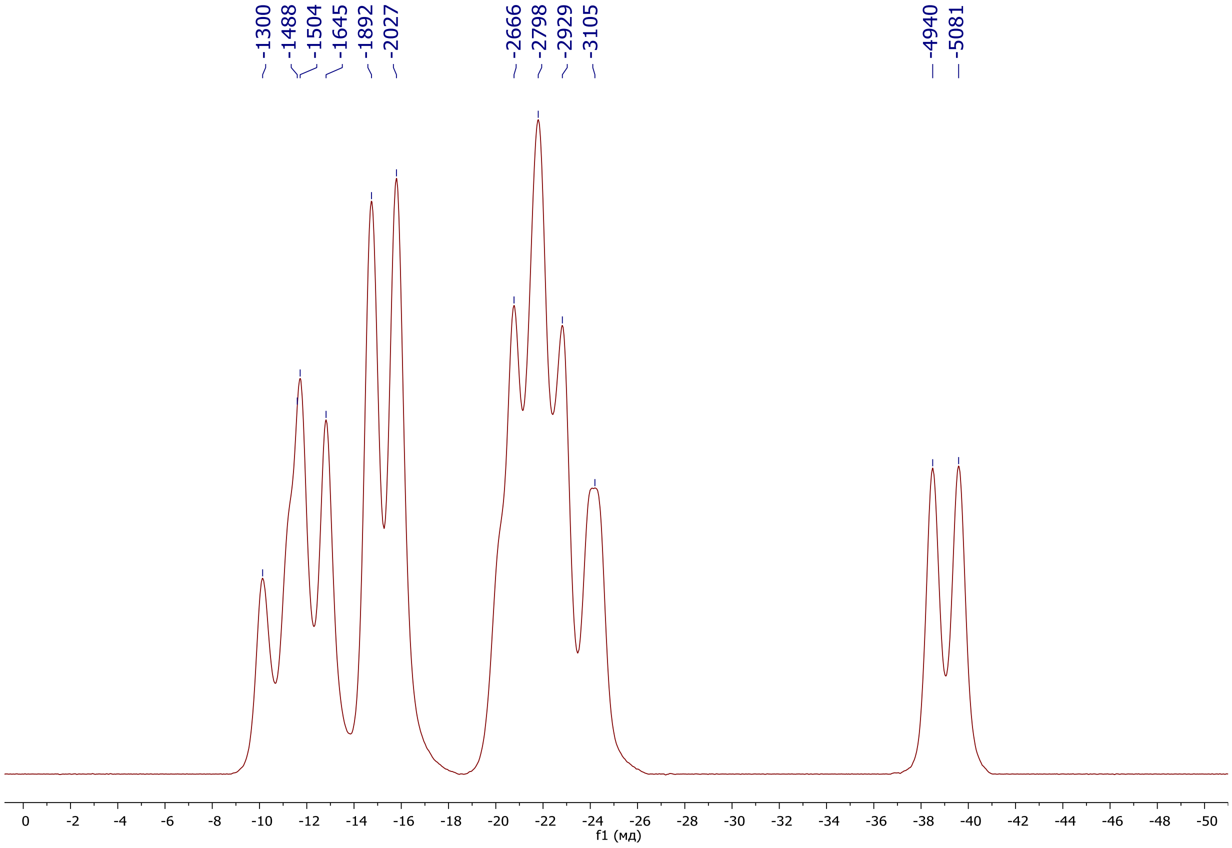

Fig. S38. ^{11}B NMR spectrum of compounds **7**.

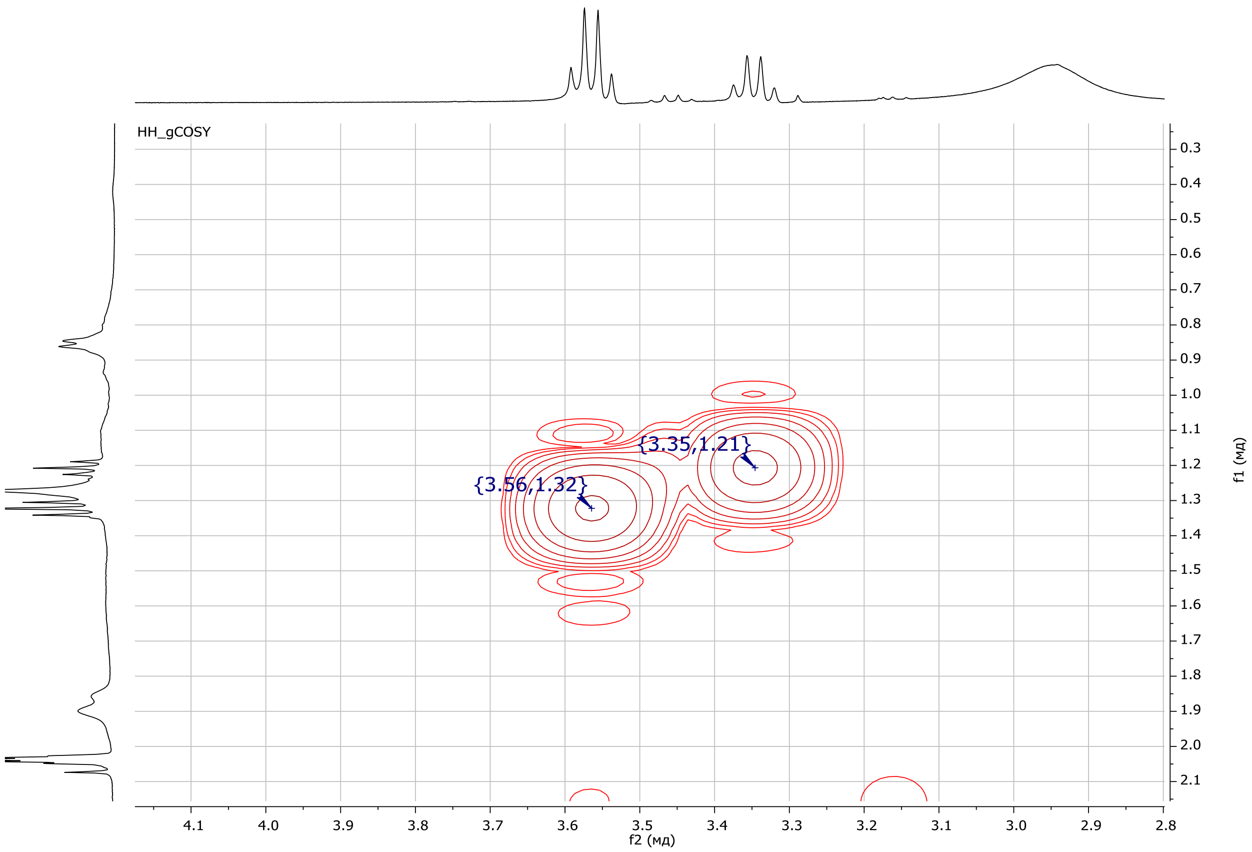

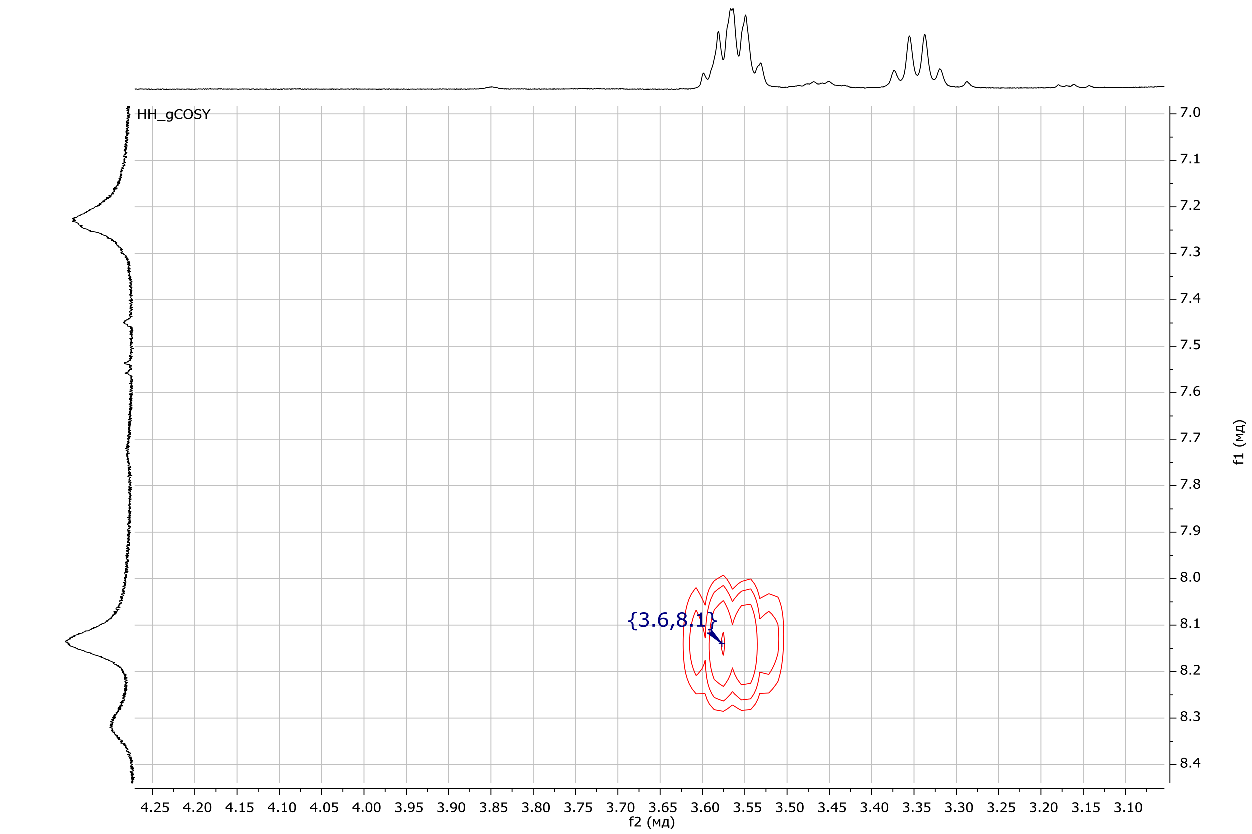

Fig. S39. ^{1}H-^{1}H NMR COSY-spectrum of compounds **7**.

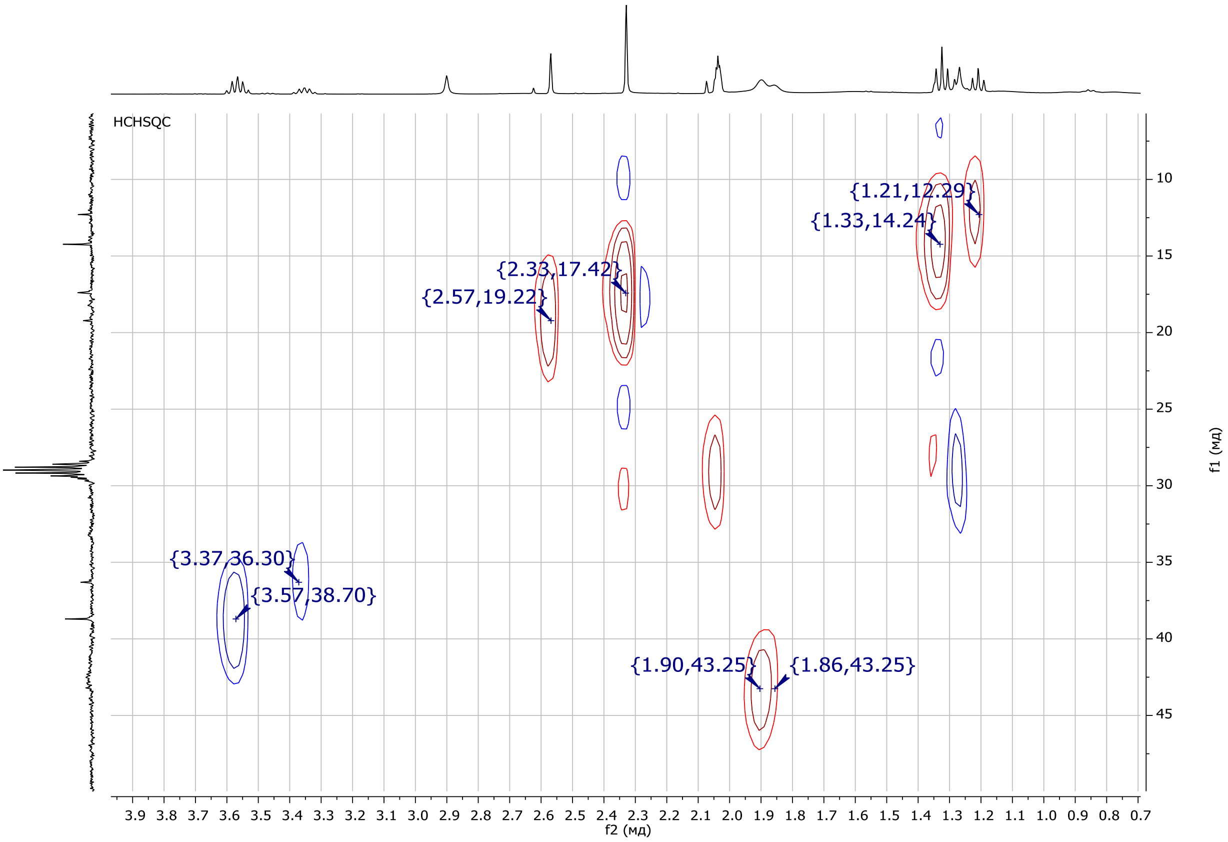

Fig. S40. ^{1}H-^{13}C NMR HSQC-spectrum of compounds **7**.

10-MeC(NMe$_2$)=HN-7,8-C$_2$B$_9$H$_{11}$ (8)

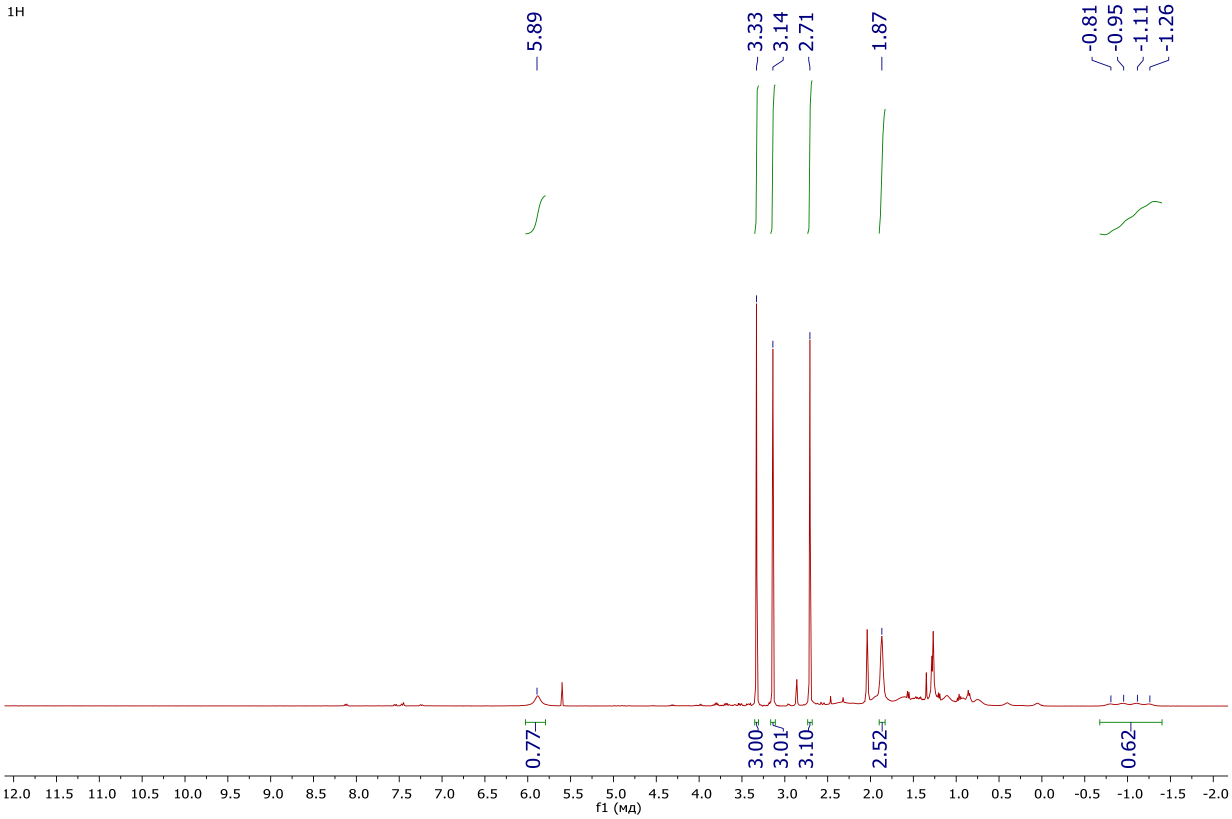

Fig. S41. ^{1}H NMR spectrum of compound **8**.

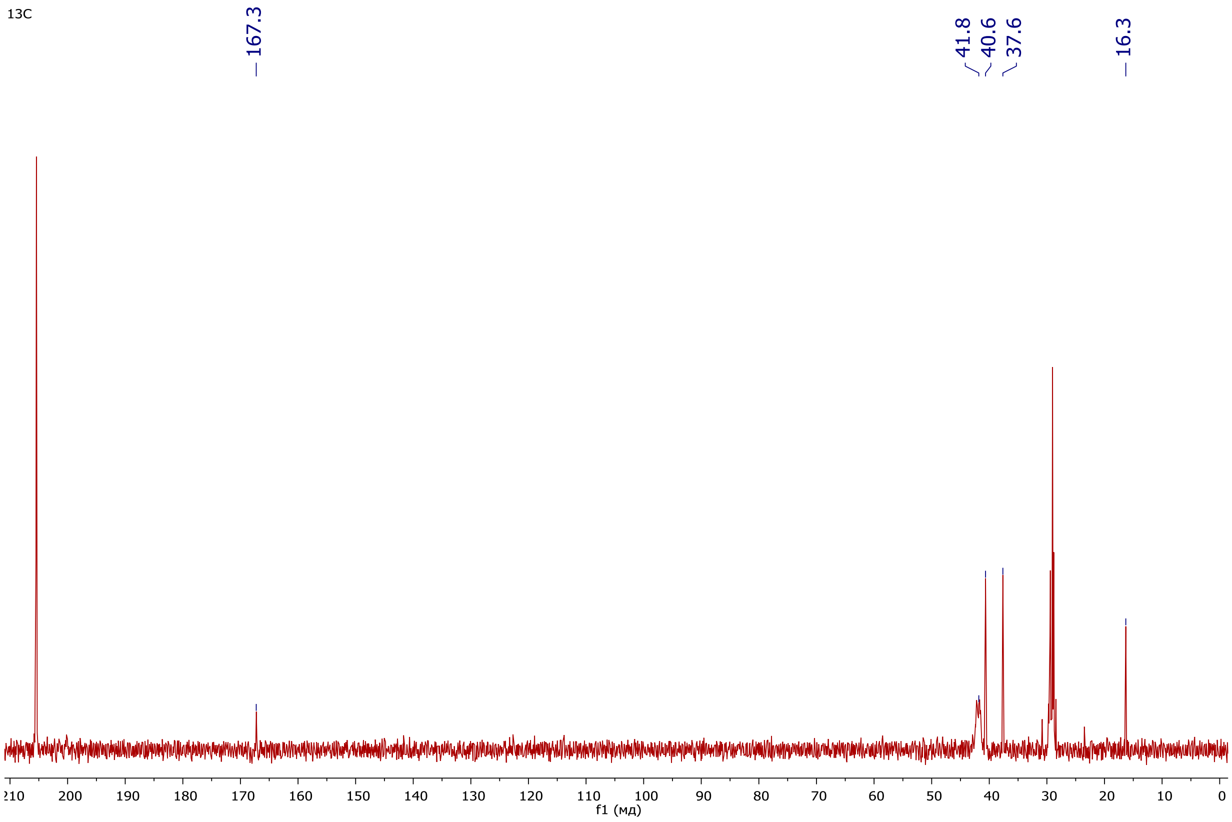

Fig. S42. ^{13}C NMR spectrum of compound **8**.

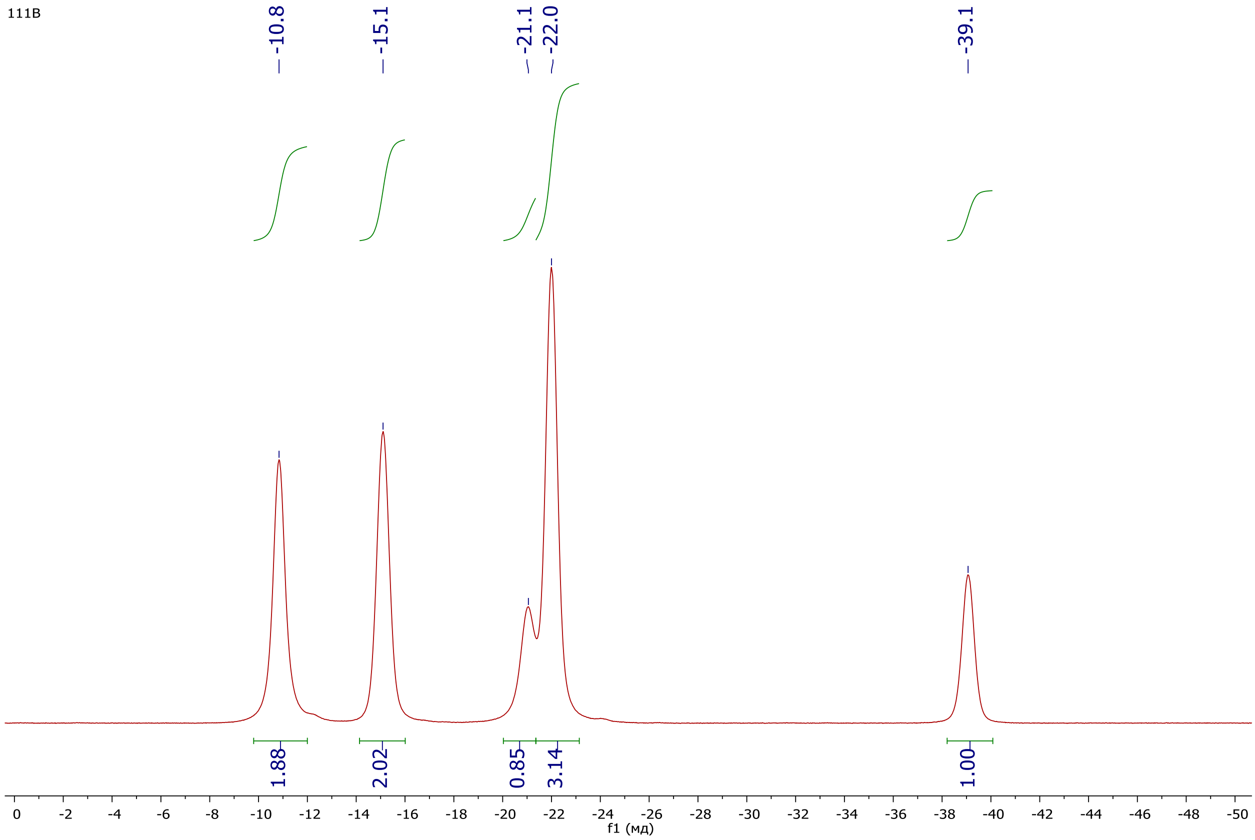

Fig. S43. $^{11}B\{^{1}H\}$ NMR spectrum of compound **8**.

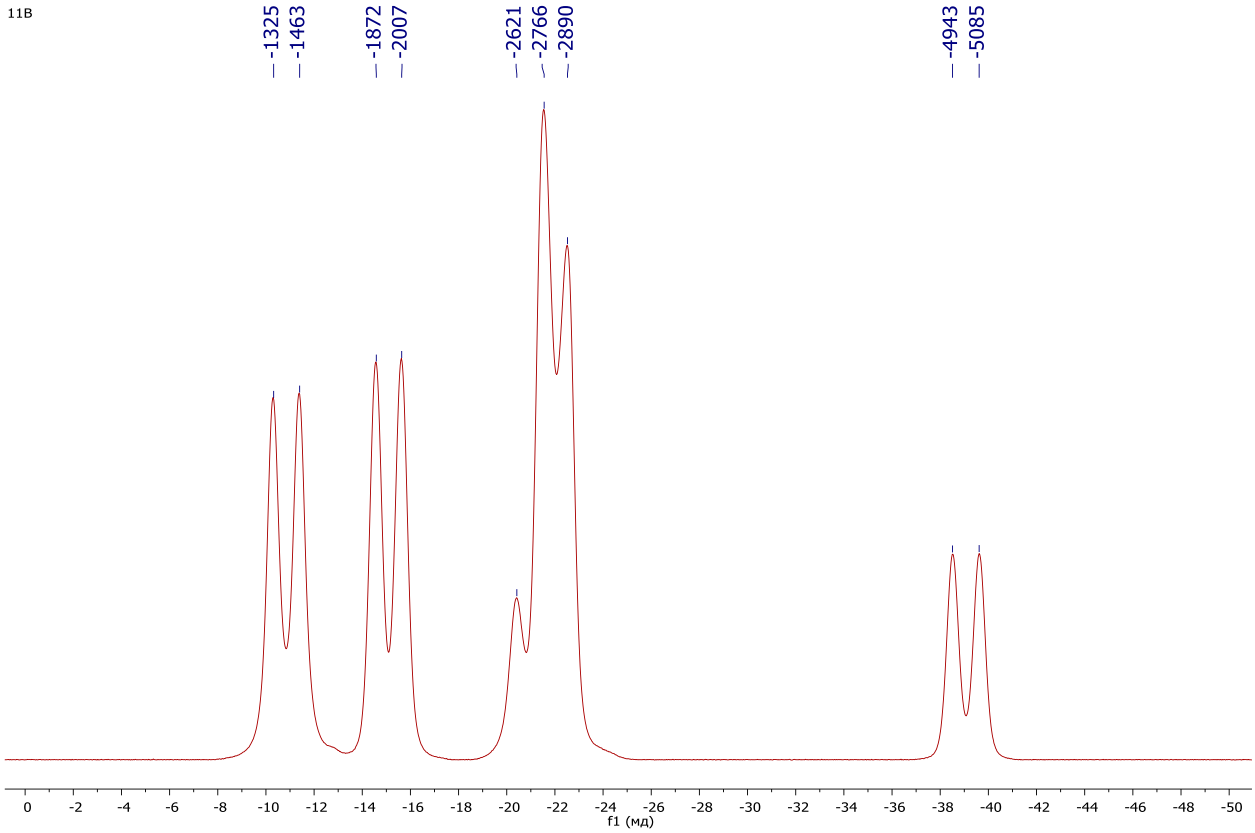

Fig. S44. ^{11}B NMR spectrum of compound **8**.

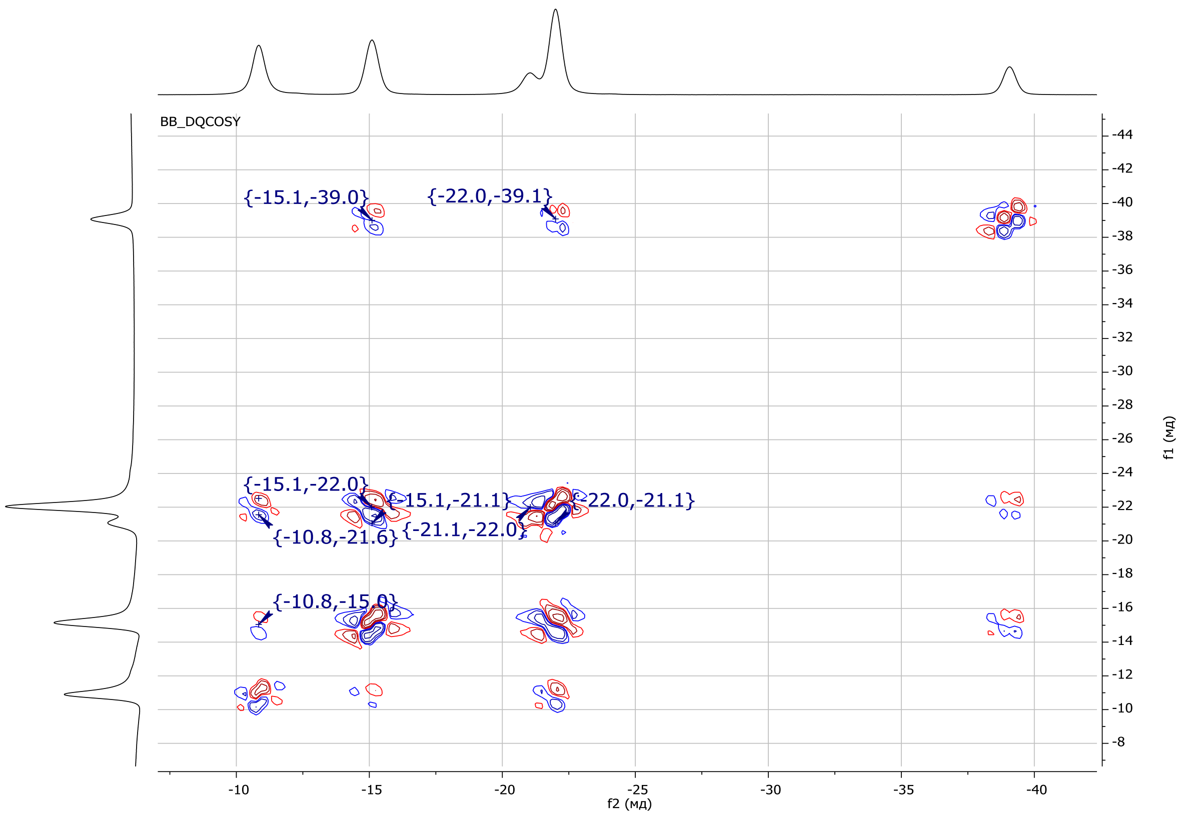

Fig. S45. ^{11}B-^{11}B COSY-spectrum of compound **8**.

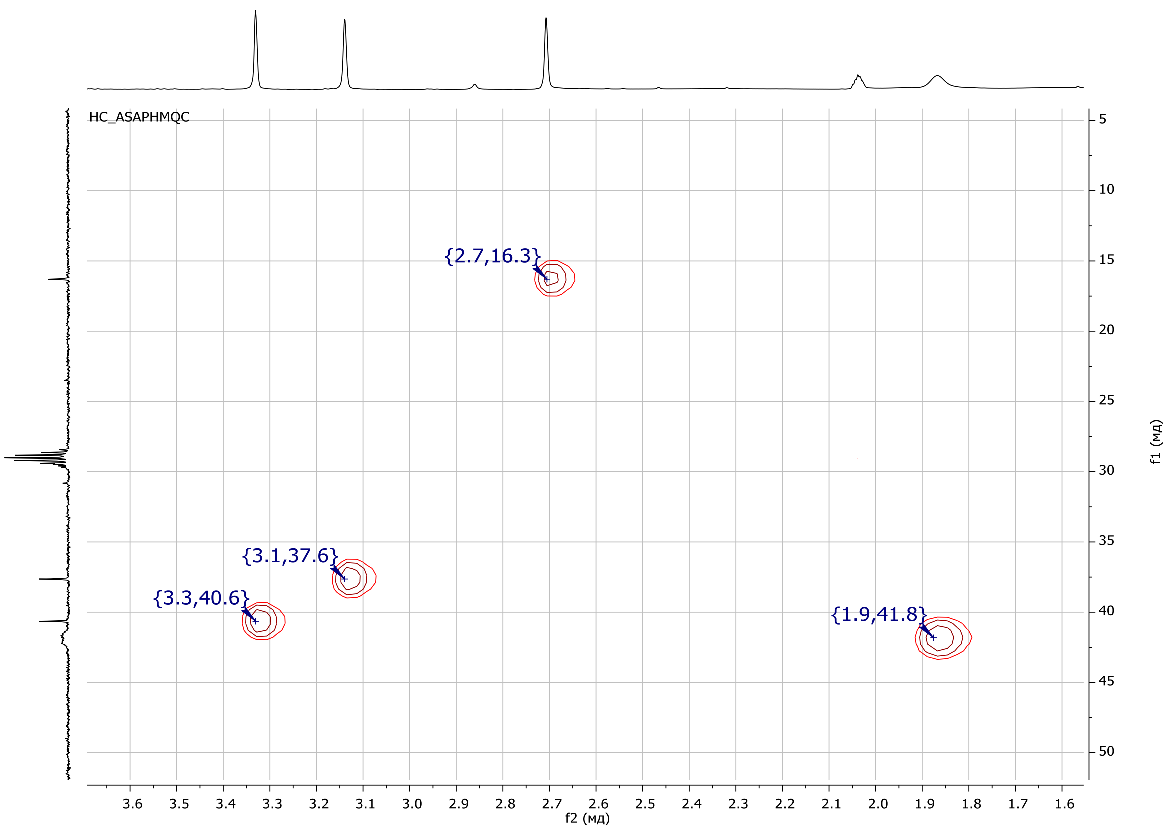

Fig. S46. ^{1}H-^{13}C NMR HMQC-spectrum of compound **8**.

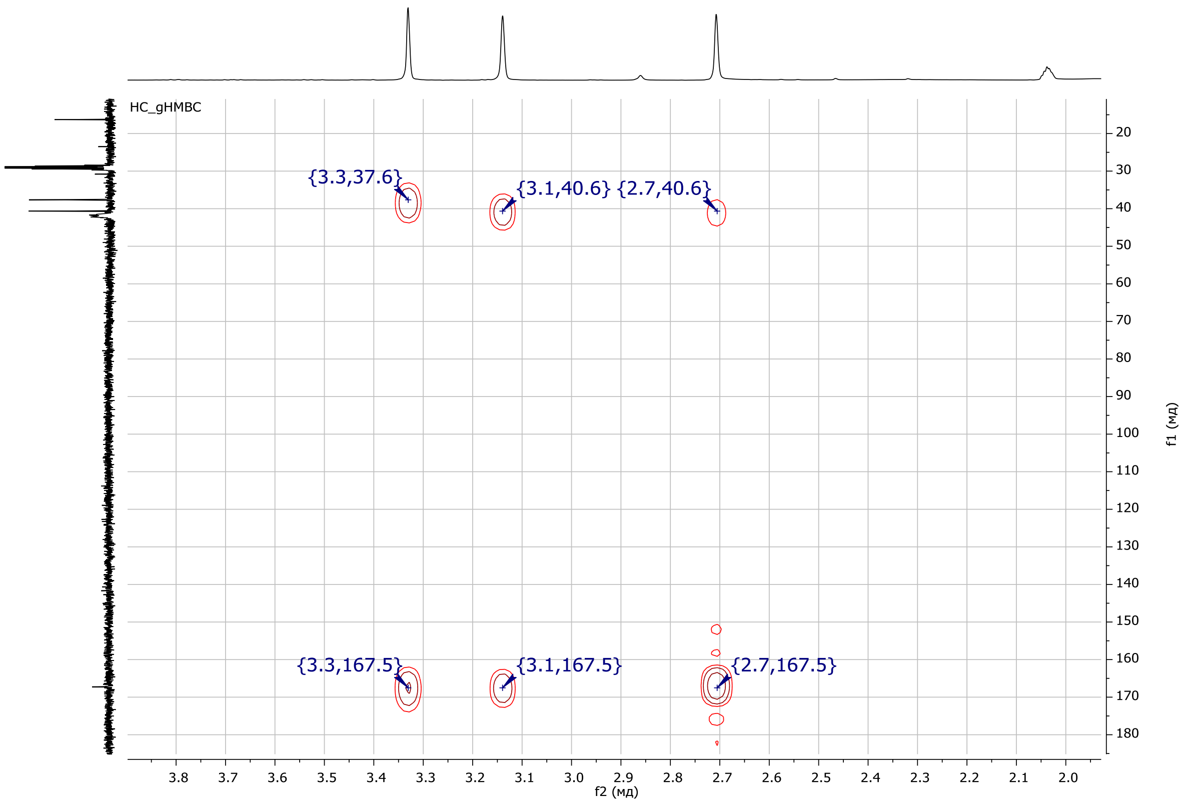

Fig. S47. ^{1}H-^{13}C HMBC-spectrum of compound **8**.

$10\text{-}MeC(NEt_2){=}HN\text{-}7,8\text{-}C_2B_9H_{11}$ (9)

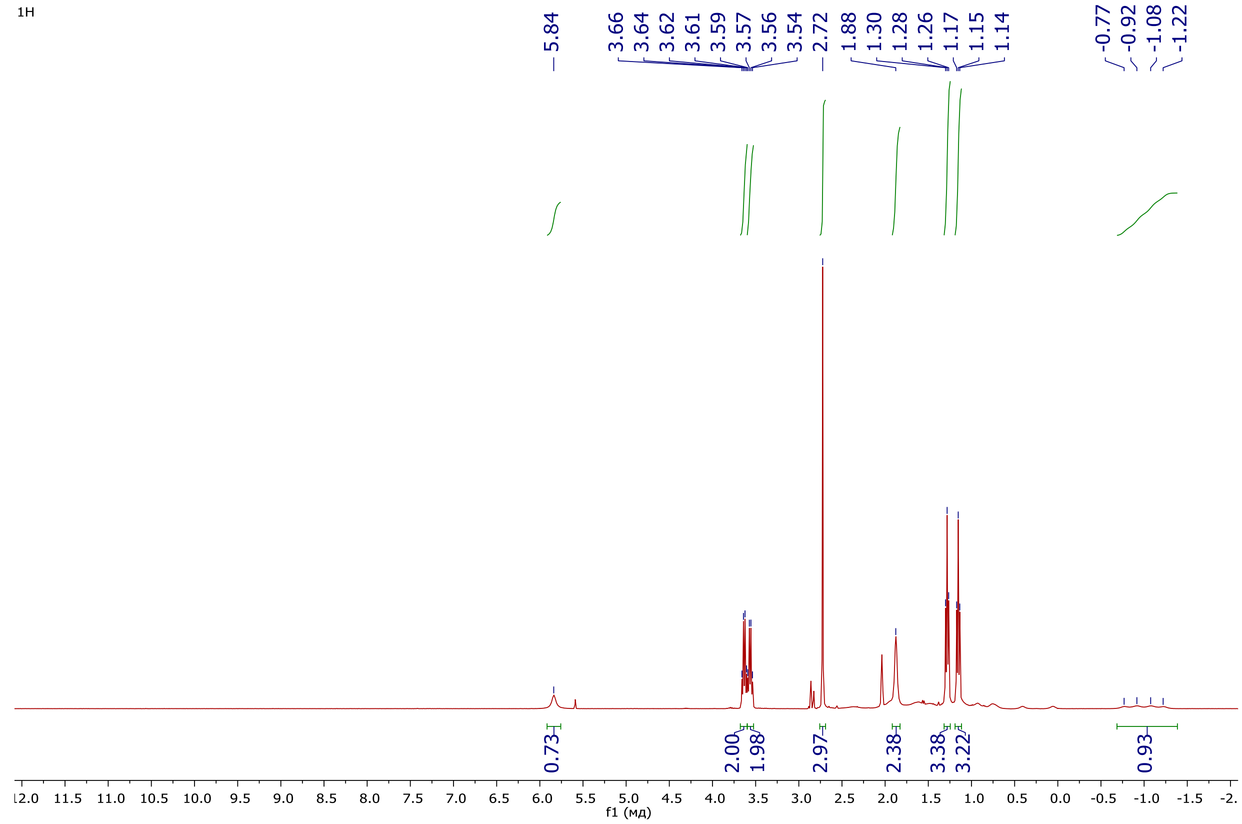

Fig. S48. ^{1}H NMR spectrum of compound **9**.

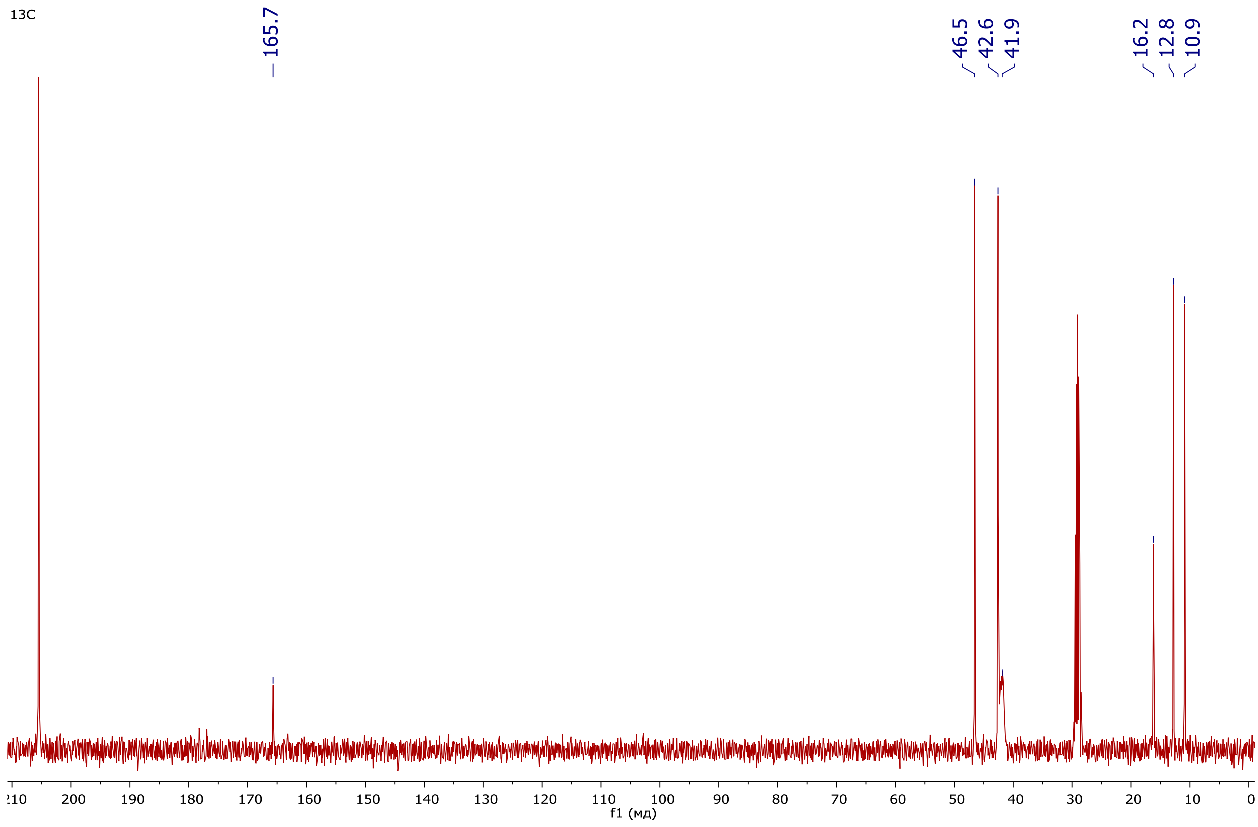

Fig. S49. ^{13}C NMR spectrum of compound **9**.

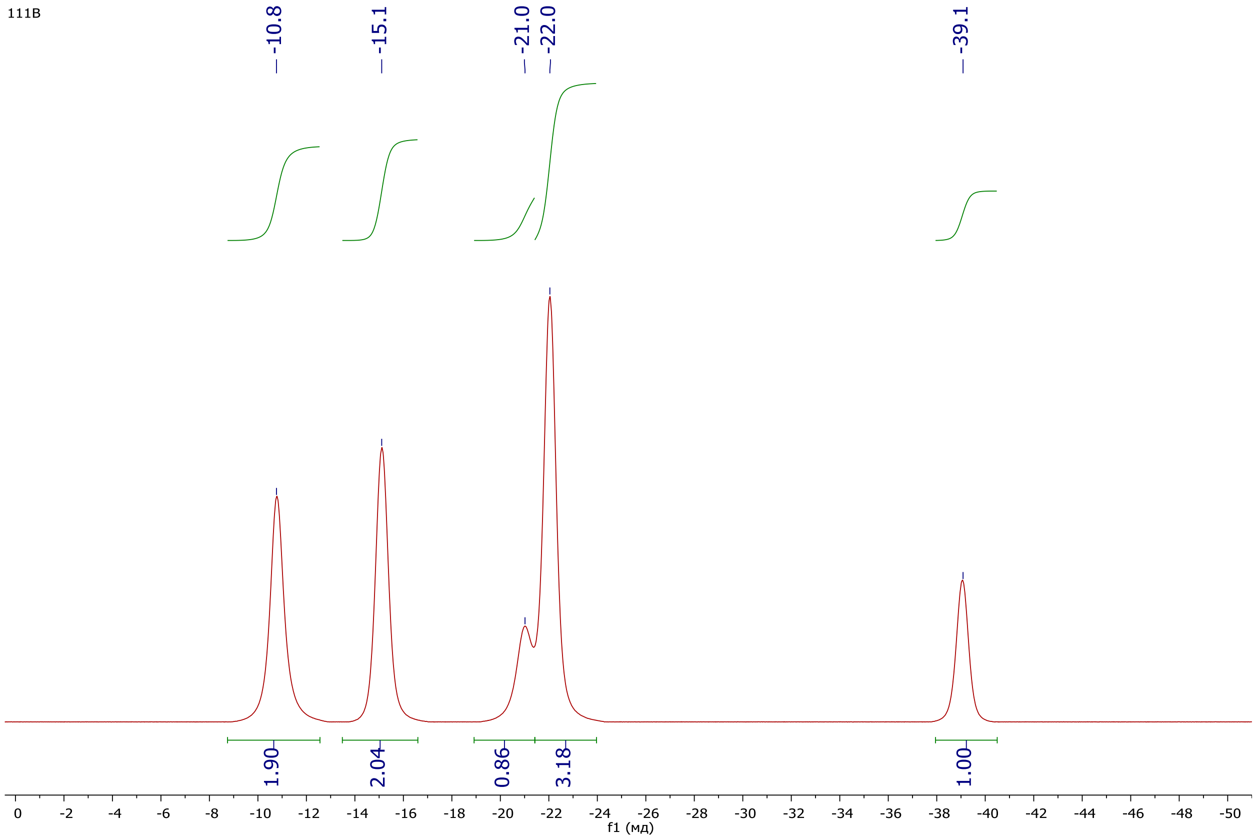

Fig. S50. $^{11}B\{^{1}H\}$ NMR spectrum of compound **9**.

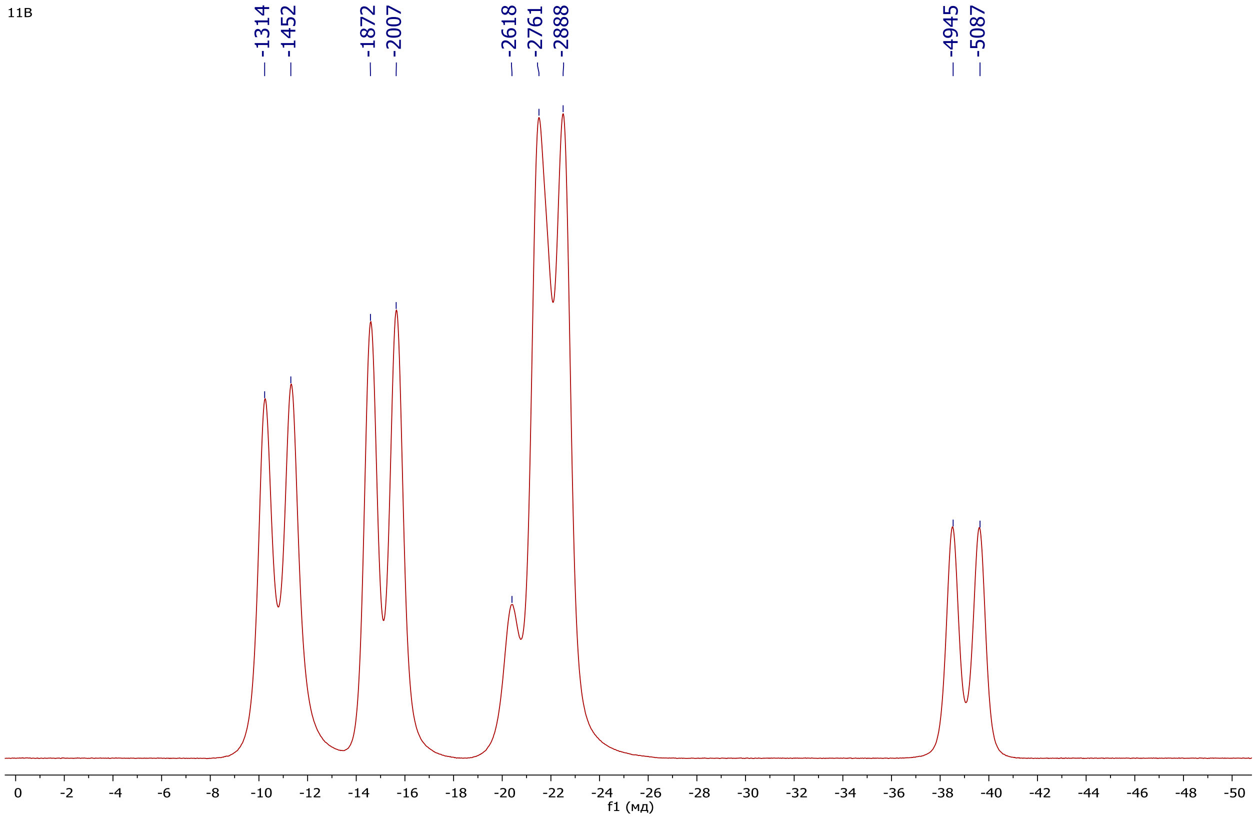

Fig. S51. ^{11}B NMR spectrum of compound **9**.

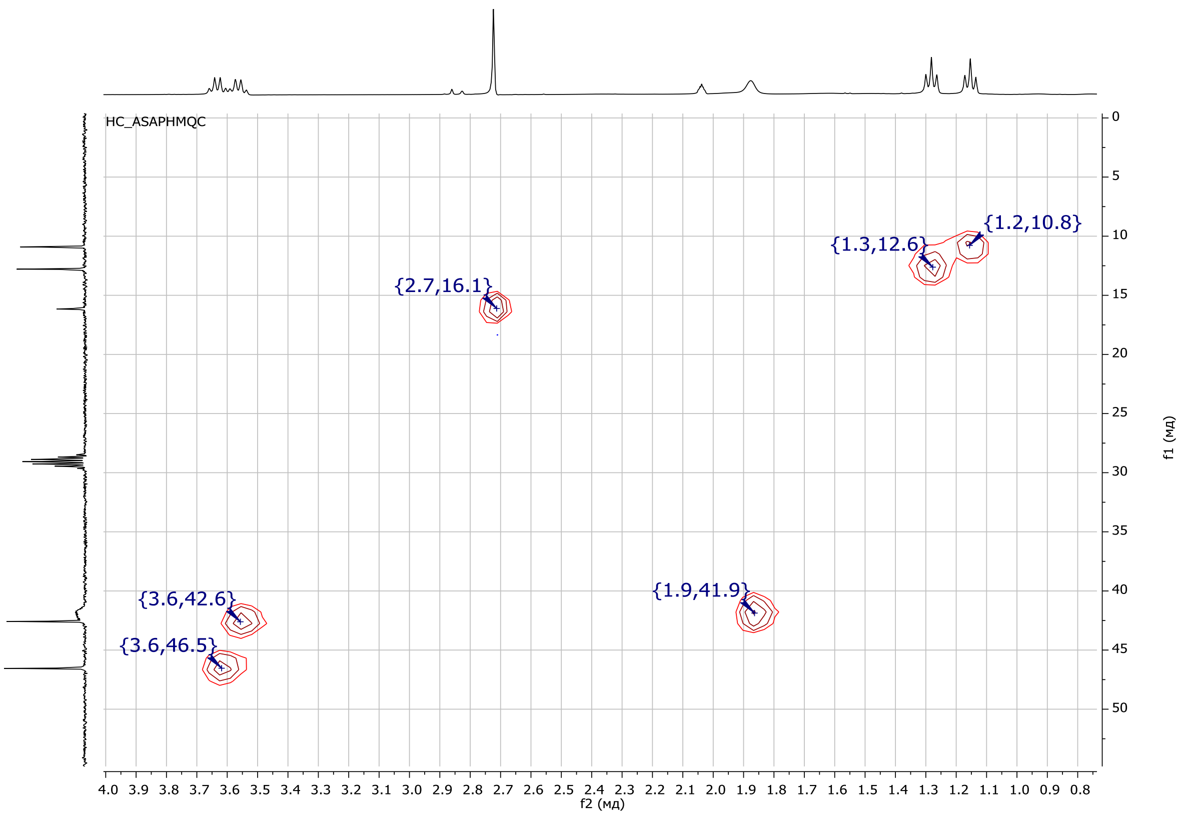

Fig. S52. ^{1}H-^{13}C NMR HMQC-spectrum of compound **9**.

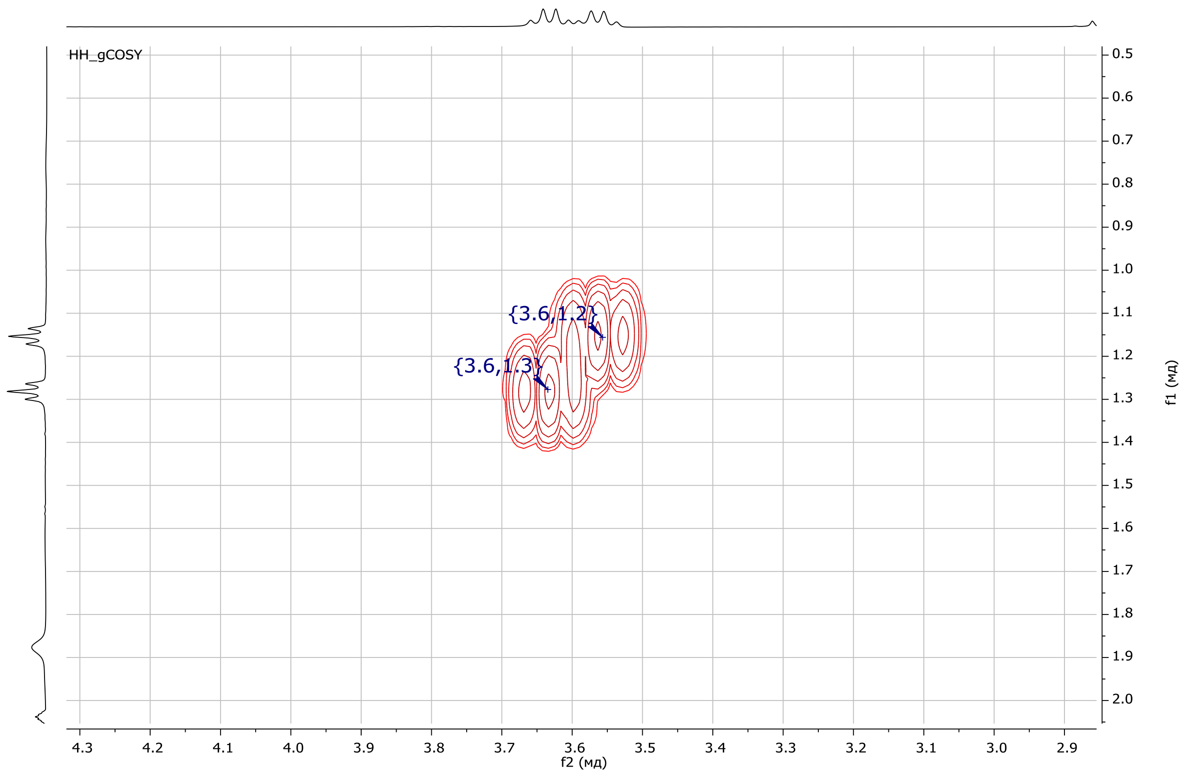

Fig. S53. ^{1}H-^{1}H NMR COSY-spectrum of compound **9**.

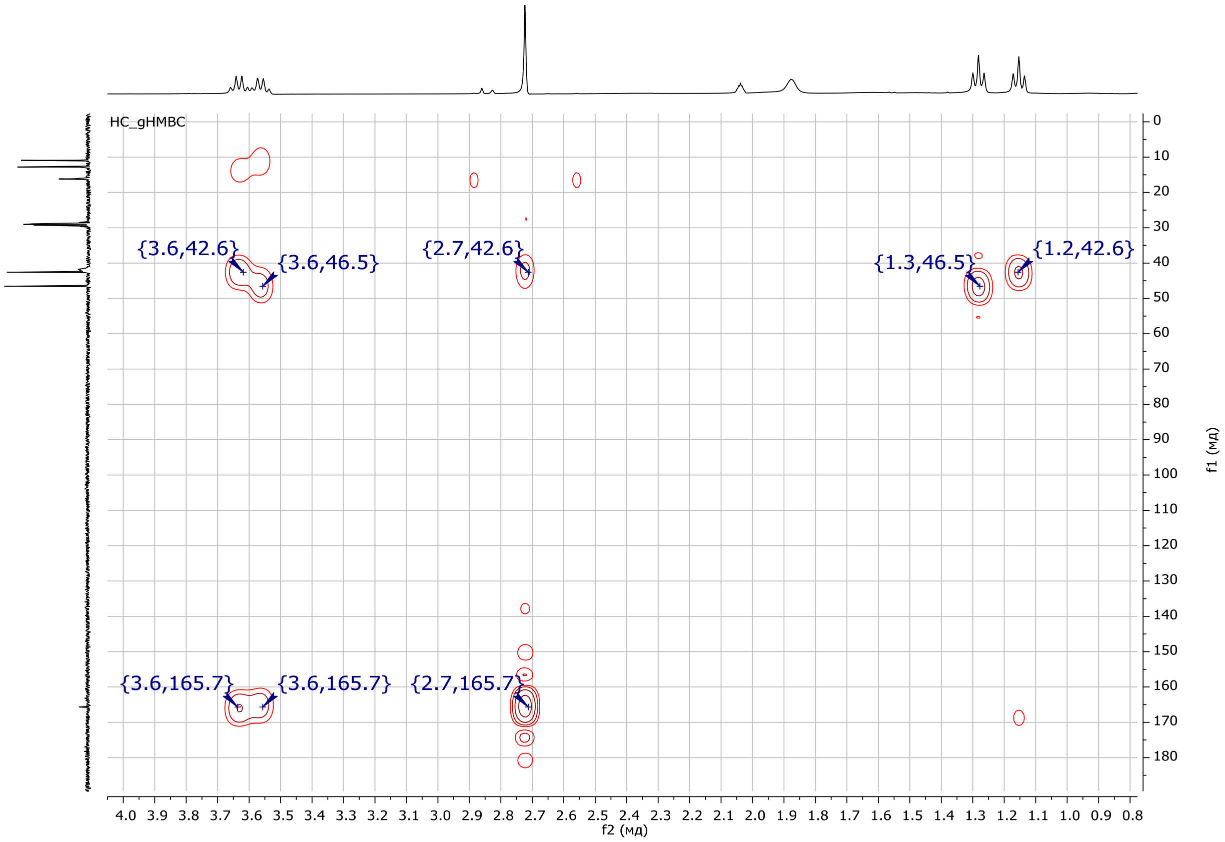

Fig. S54. ^{1}H-^{13}C HMBC-spectrum of compound **9**.

10-MeC(N(CH2)5)=HN-7,8-C2B9H11 (10)

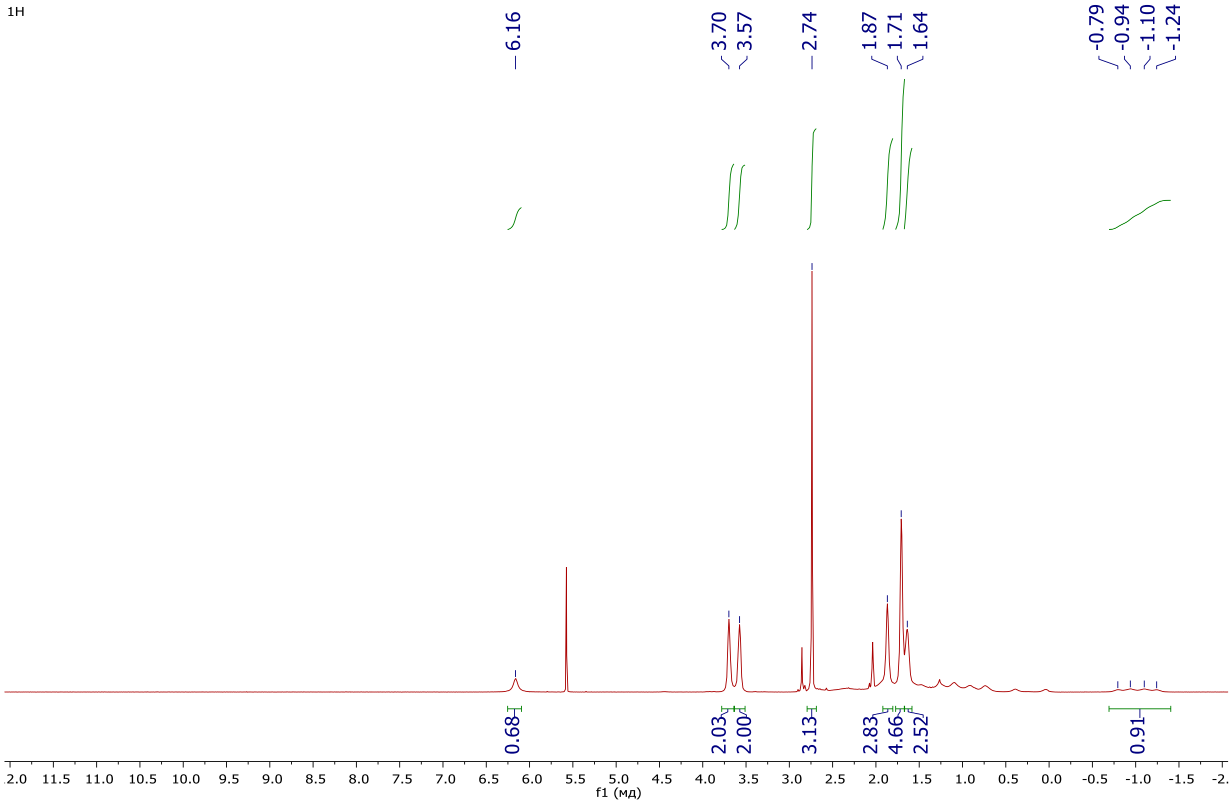

Fig. S55. ^{1}H NMR spectrum of compound **10**.

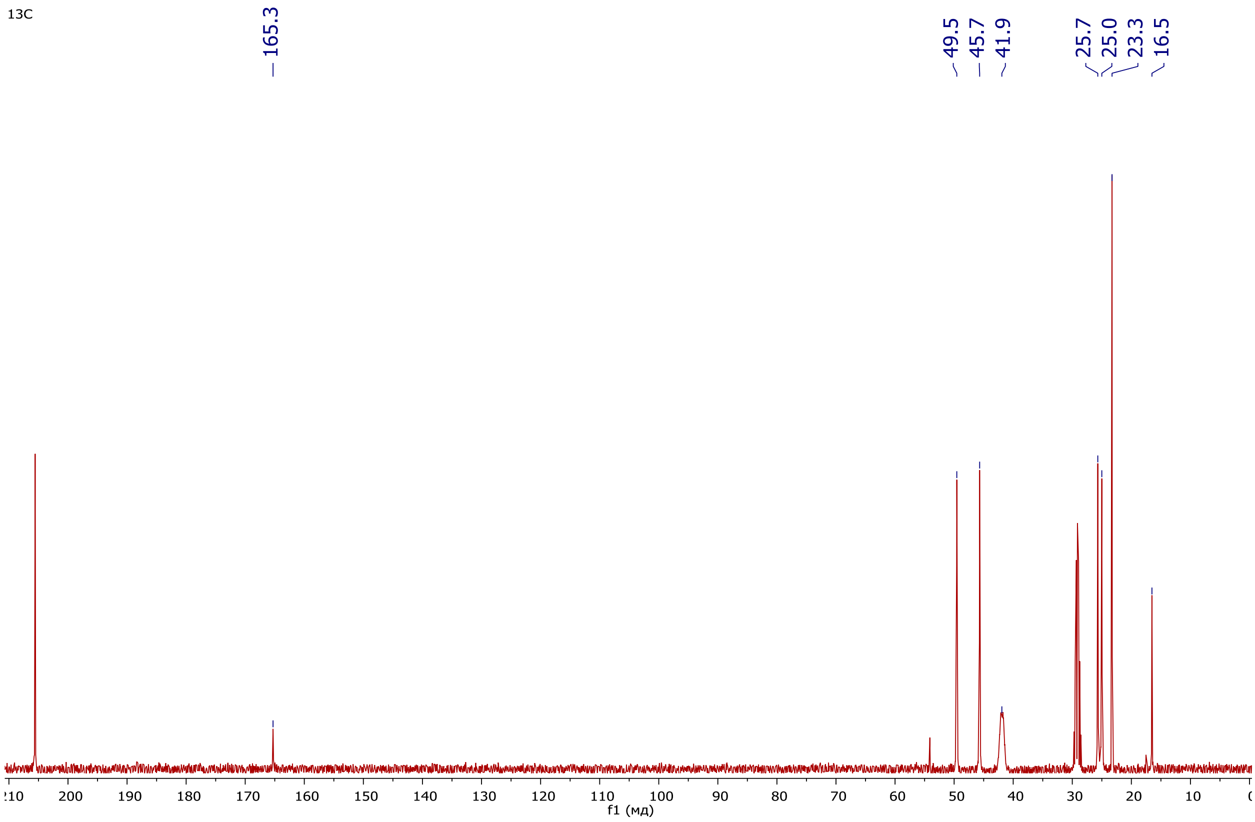

Fig. S56. ^{13}C NMR spectrum of compound **10**.

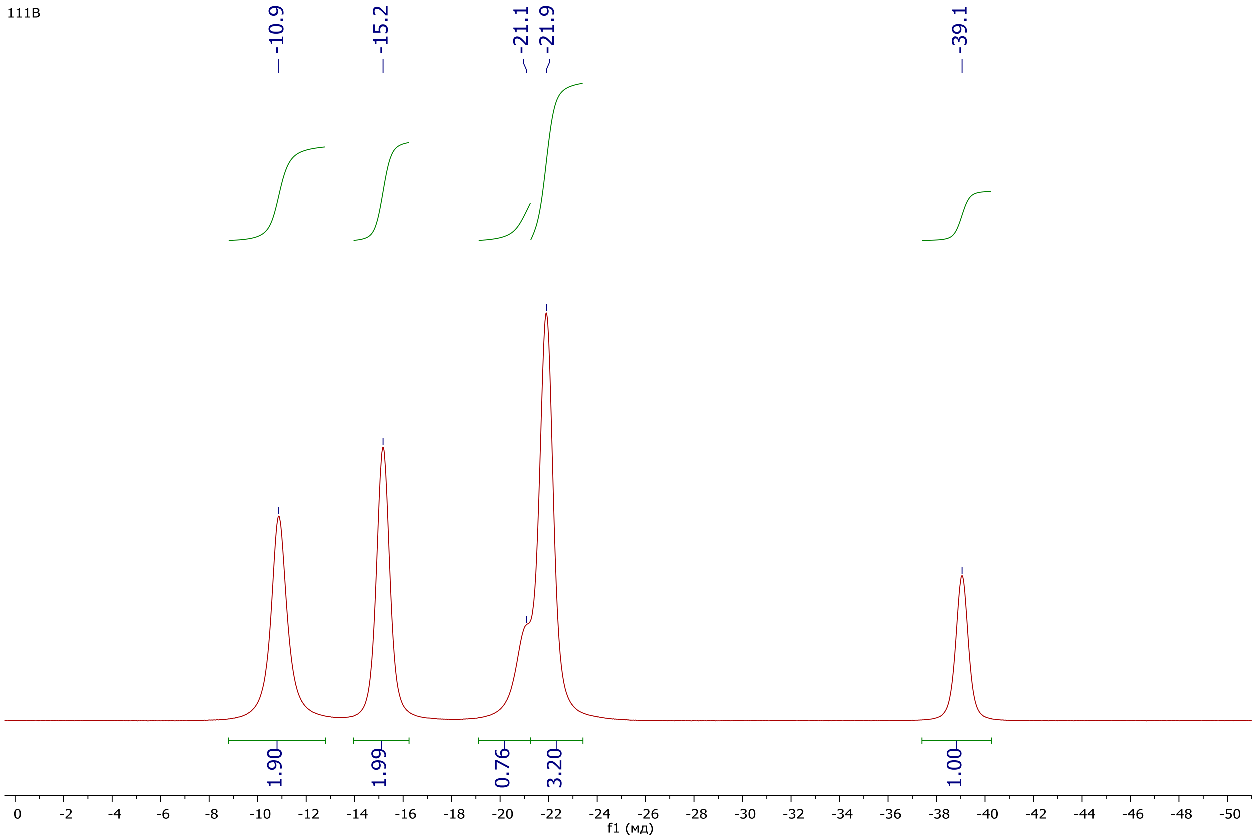

Fig. S57. $^{11}B\{^{1}H\}$ NMR spectrum of compound **10**.

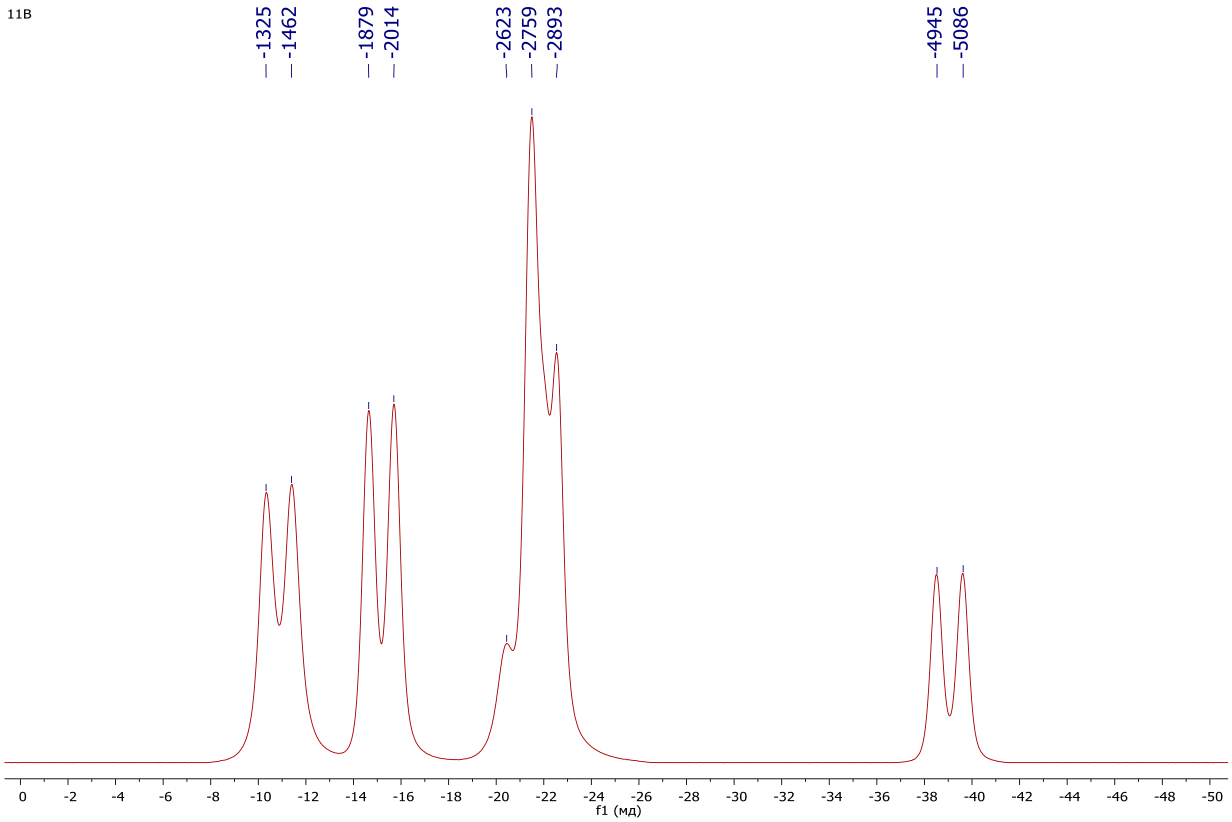

Fig. S58. ^{11}B NMR spectrum of compound **10**.

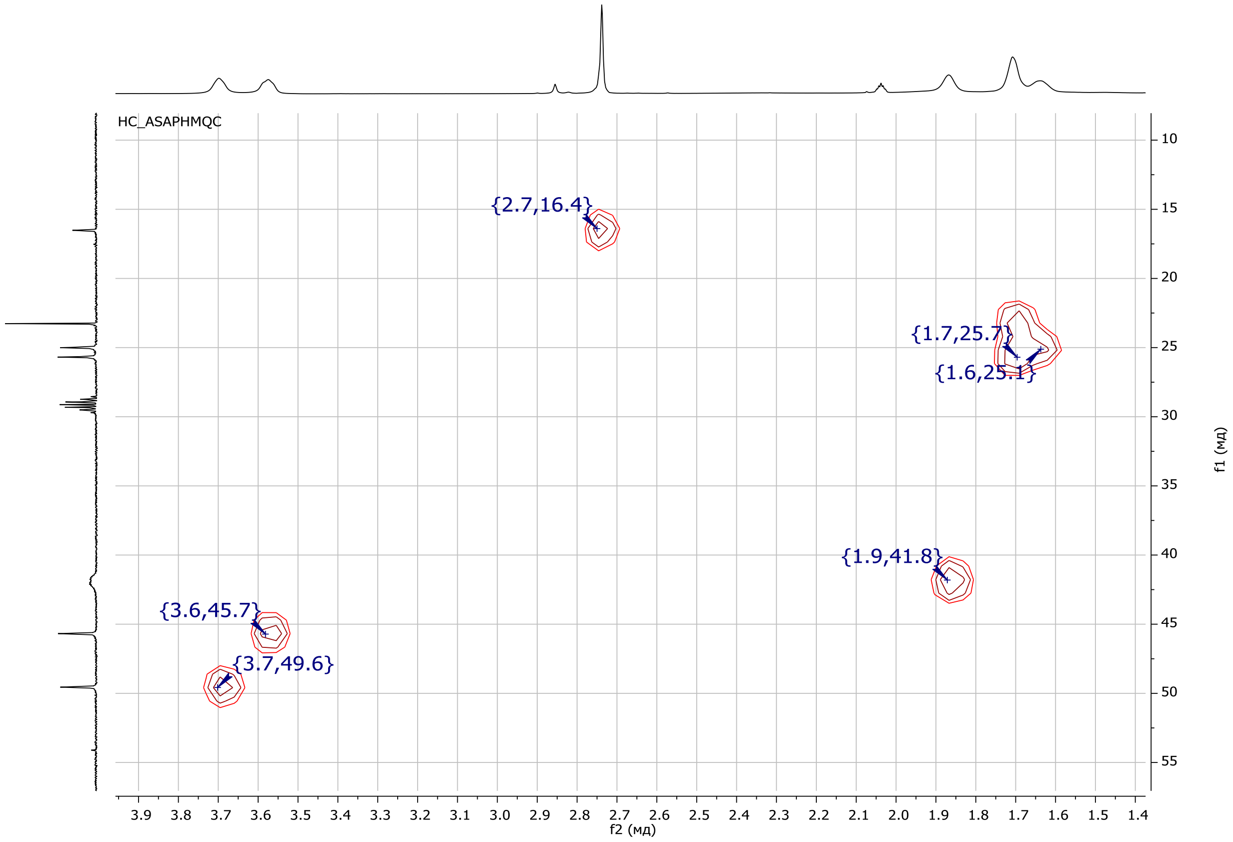

Fig. S59. ^{1}H-^{13}C NMR HMQC-spectrum of compound **10**.

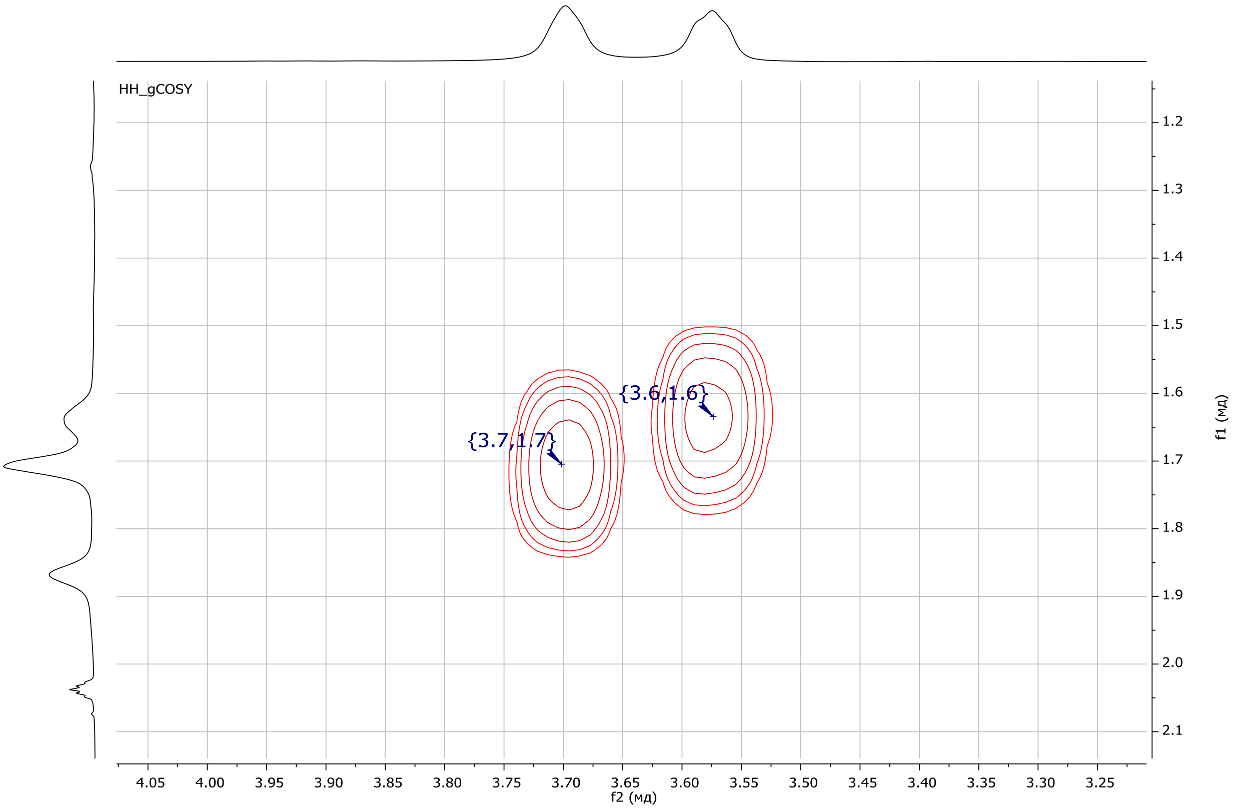

Fig. S60. ^{1}H-^{1}H NMR COSY-spectrum of compound **10**.

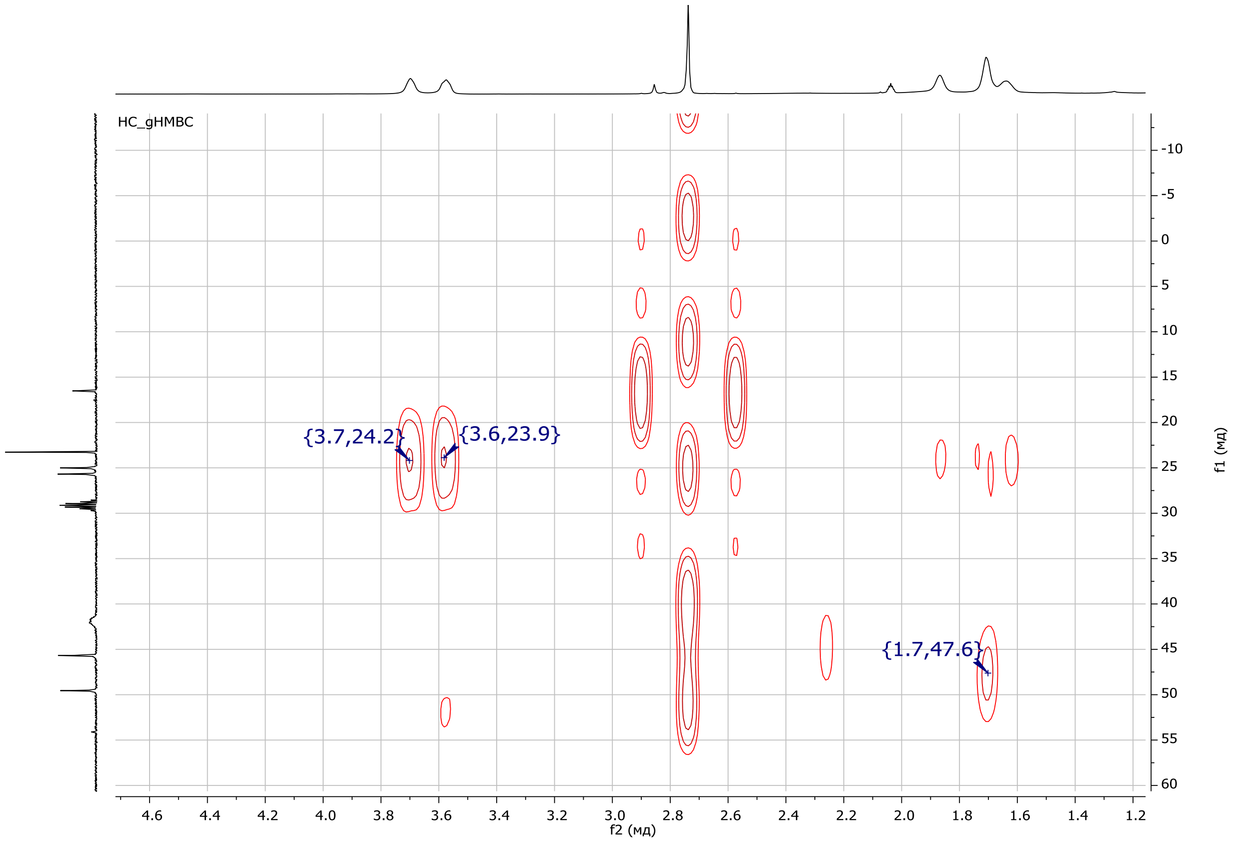

Fig. S61. ^{1}H-^{13}C HMBC-spectrum of compound **10**.

10-MeC(N(CH2)2O(CH2)2)=HN-7,8-C2B9H11 (11)

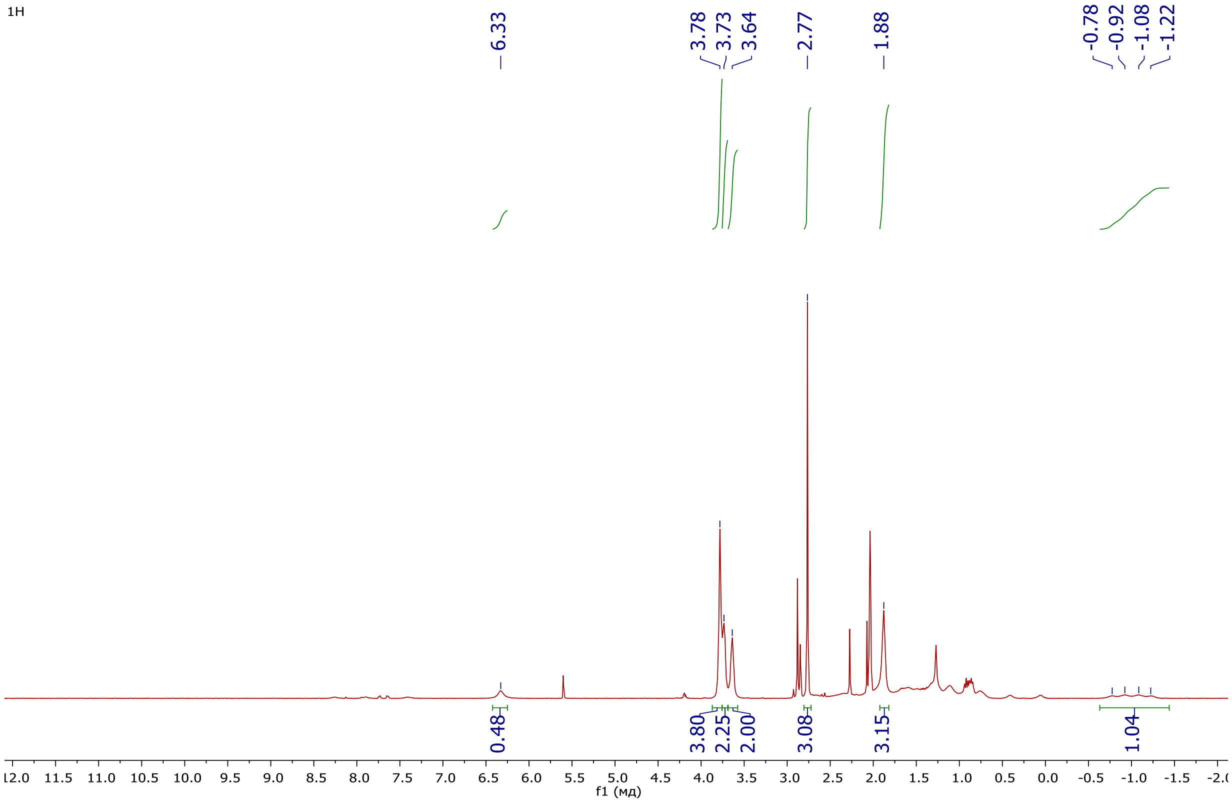

Fig. S62. ^{1}H NMR spectrum of compound **11**.

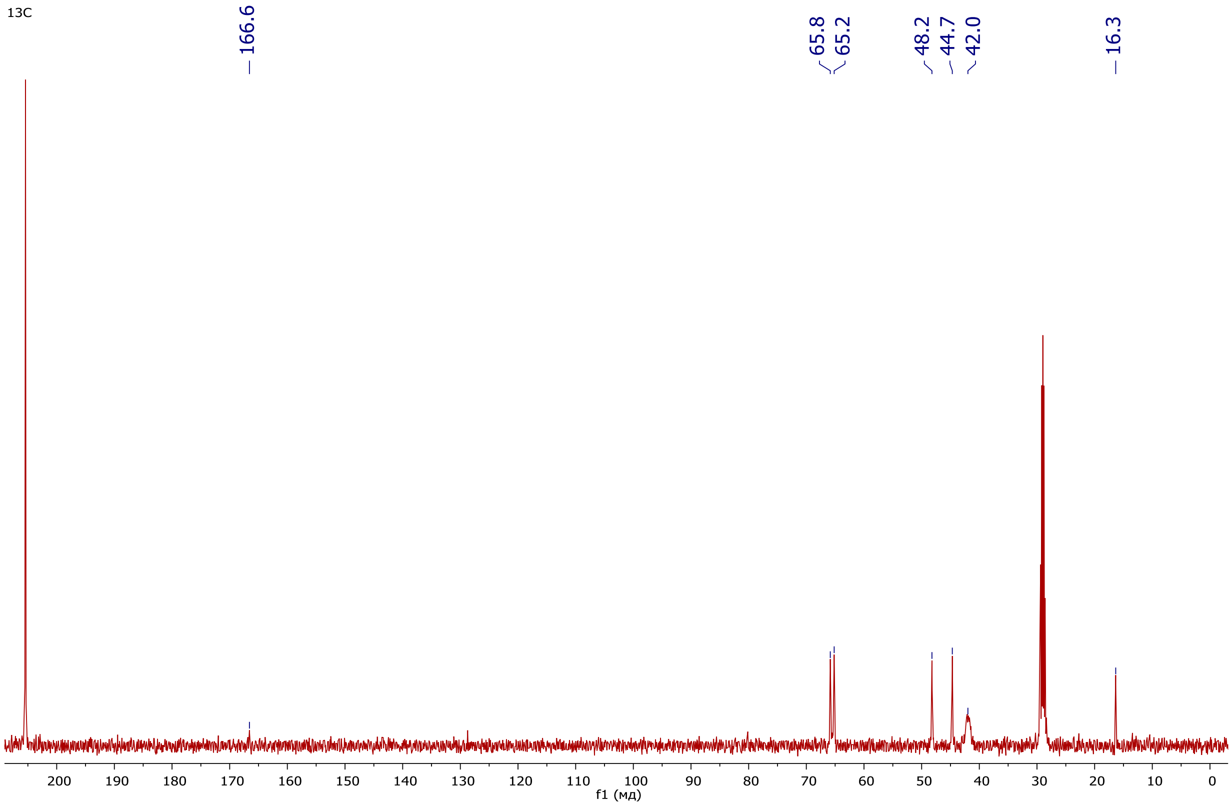

Fig. S63. ^{13}C NMR spectrum of compound **11**.

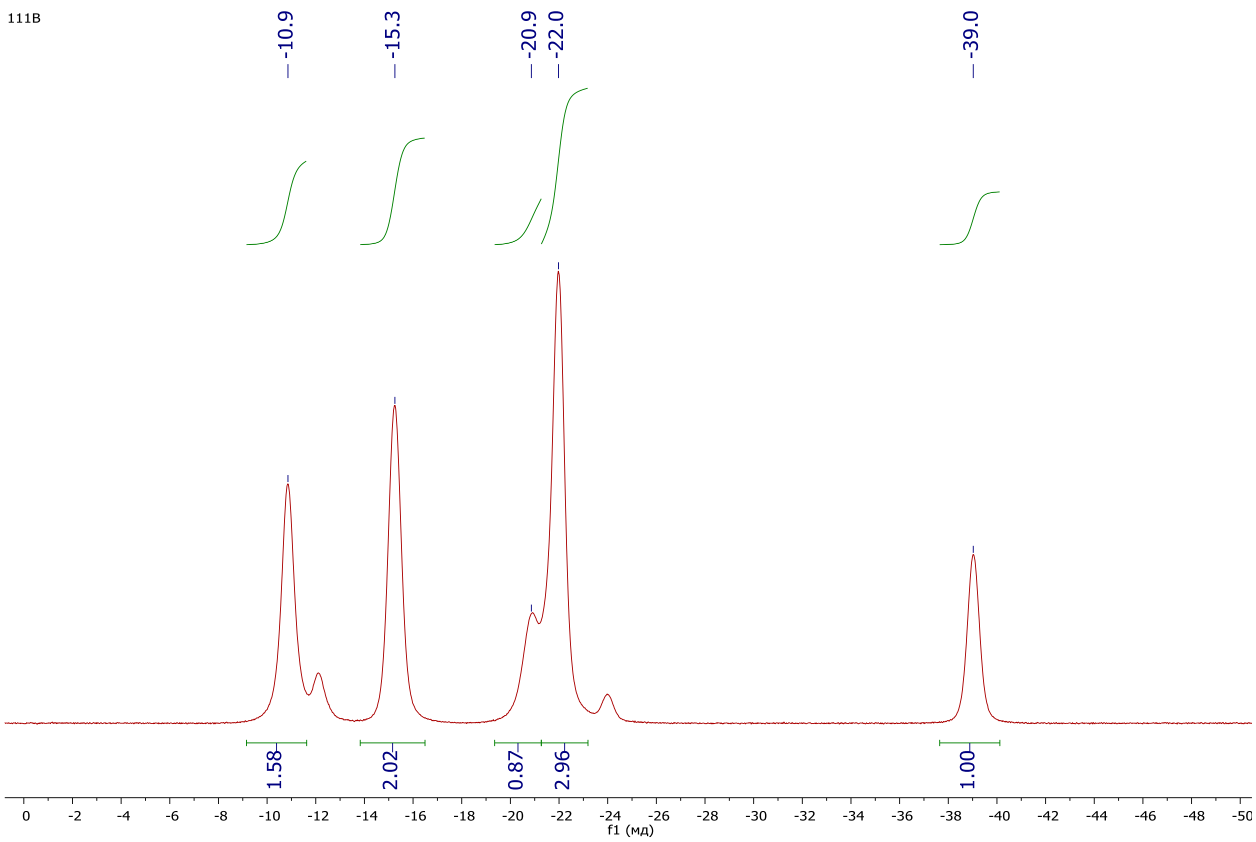

Fig. S64. $^{11}B\{^{1}H\}$ NMR spectrum of compound **11**.

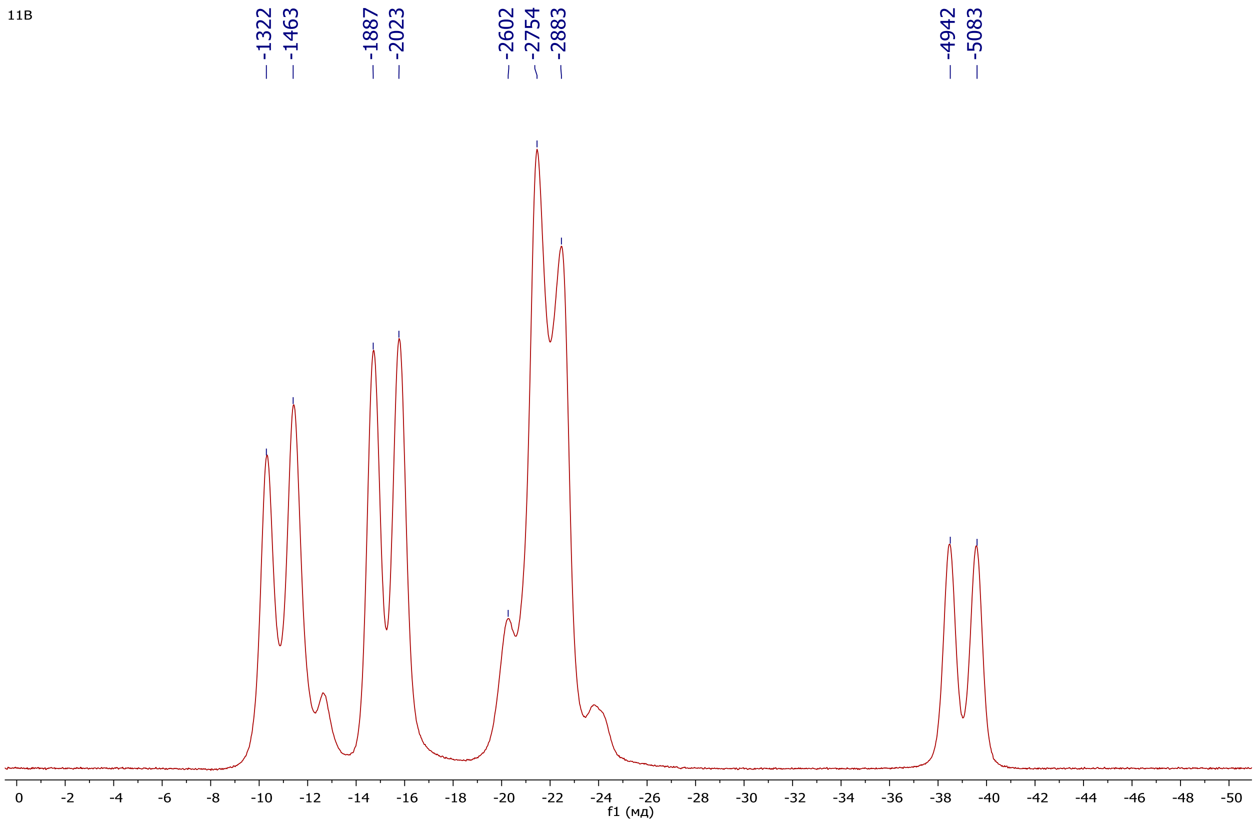

Fig. S65. ^{11}B NMR spectrum of compound **11**.

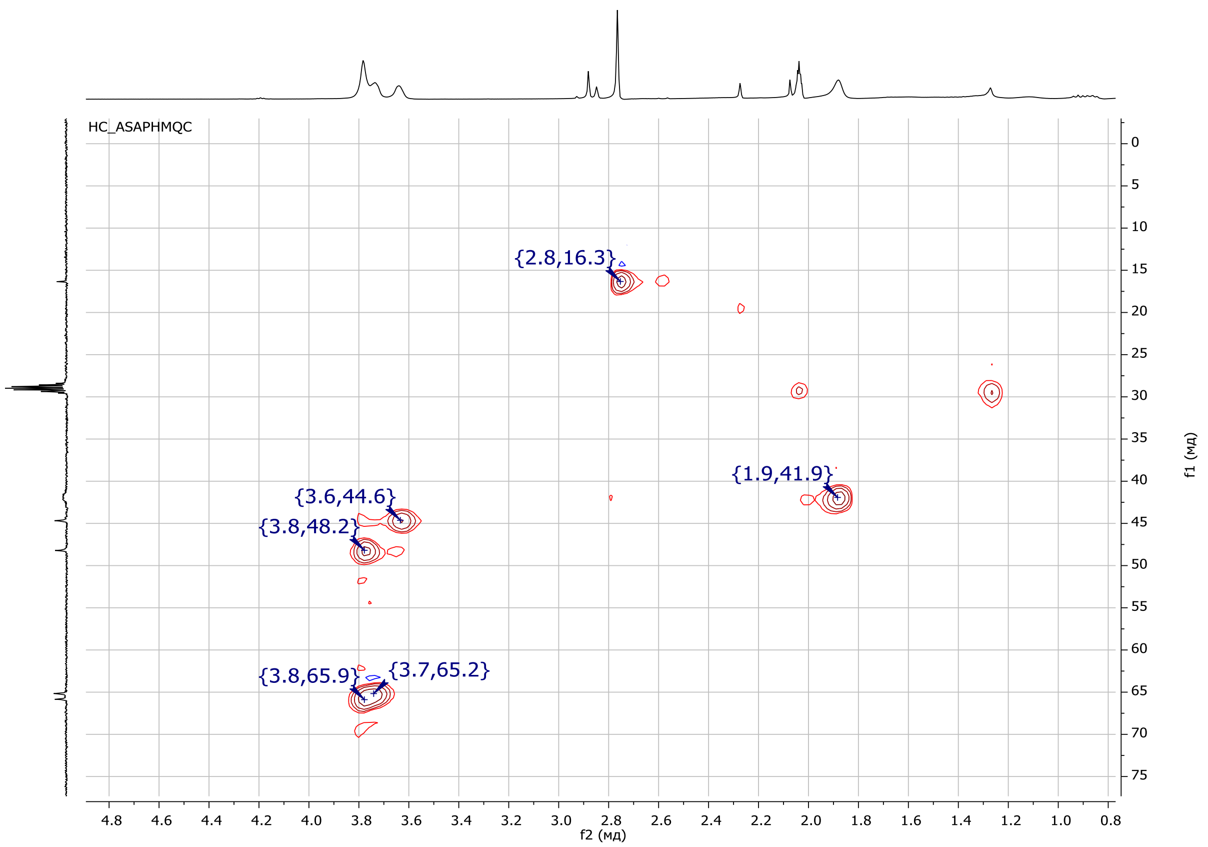

Fig. S66. ^{1}H-^{13}C NMR HMQC-spectrum of compound **11**.

Table S1. Crystallographic data for compounds **4**, **6**, **7**.

	Compound **4**	Compound **6**	Compound **7**
formula	$C_5H_{18}B_9NO$	$C_5H_{19}B_9N_2$	$C_8H_{25}B_9N_2$
fw	205.49	204.51	218.54
crystal system	orthorhombic	orthorhombic	monoclinic
space group	$Pna2_1$	$P2_12_12_1$	$P2_1/c$
a, Å	10.5157(3)	7.2332(2)	8.4506(6)
b, Å	8.3588(2)	10.6054(3)	7.3489(5)
c, Å	13.2738(3)	15.6957(4)	10.7658(7)
α, deg.	90	90	90
β, deg.	90	90	100.531(2)
γ, deg.	90	90	90
V, Å^3	1166.75(5)	1204.03(6)	657.32(8)
Z	4	4	2
ρ_{cryst}, g·cm^{-3}	1.170	1.128	1.104
F(000)	432	432	232
μ, mm^{-1}	0.062	0.056	0.055
θ range, deg.	2.88 – 26.97	2.32 – 26.15	1.92 – 27.12
rflns collected	13890	14852	10892
indep rflns / R_{int}	2539/0.0256	2399/0.0431	2902/0.0278
Completeness to theta θ, %	100	100	99.9
ref.parameters	195	199	208
GOF (F^2)	1.101	1.092	1.144
rflns with I>2σ(I)	2483	2332	2825
$R_1(F)$ (I>2σ(I))^a	0.0266	0.0501	0.0342
$wR_2(F^2)$ (all data)^b	0.0723	0.1301	0.0845
Largest diff. peak/hole, e·Å^{-3}	0.162/-0.150	0.478/-0.183	0.166/-0.159
CCDC Number	2413512	2422305	2413510

[a] $R_1 = \sum|F_o - |F_c||/\sum(F_o)$; [b] $wR_2 = (\sum[w(F_o^2 - F_c^2)^2]/\sum[w(F_o^2)^2]^{½}$

Table S2. Crystallographic data for compounds **8-11**.

	**8**	**9**	**10**	**11**
formula	$C_6H_{21}B_9N_2$	$C_8H_{25}B_9N_2$	$C_9H_{25}B_9N_2$	$C_8H_{23}B_9N_2O$
fw	218.54	246.59	258.60	260.57
crystal system	orthorhombic	monoclinic	triclinic	monoclinic
space group	$P2_12_12_1$	$P2_1/c$	P-1	$P2_1/n$
a, Å	9.5589(7)	13.5346(4)	10.3110(3)	11.0904(7)
b, Å	10.2643(8)	13.7950(4)	11.4811(4)	9.7726(6)
c, Å	13.1586(10)	16.1527(5)	13.7651(4)	14.2298(9)
α, deg.	90	90	69.5895(11)	90
β, deg.	90	95.5517(12)	89.8459(12)	103.511(2)
γ, deg.	90	90	86.1097(13)	90
V, $Å^3$	1291.06(17)	3001.72(16)	1523.33(8)	1499.6(2)
Z	4	8	4	4
ρ_{cryst}, g·cm^{-3}	1.124	1.091	1.128	1.154
F(000)	464	1056	552	552
μ, mm^{-1}	0.056	0.055	0.057	0.063
θ range, deg.	2.52 – 28.03	1.95 – 27.06	1.90 – 26.16	2.11 – 27.23
rflns collected	29117	39371	25326	15720
indep rflns / R_{int}	3125/0.0493	6575/0.0621	6086/0.0512	3328/0.0431
Completeness to theta θ, %	99.9	99.7	99.9	99.4
ref.parameters	205	445	459	230
GOF (F^2)	1.104	1.045	1.025	1.037
rflns with I>2σ(I)	2943	5418	4460	2724
$R_1(F)$ (I>2σ(I))[a]	0.0339	0.0430	0.0466	0.0399
$wR_2(F^2)$ (all data)[b]	0.0938	0.1155	0.1143	0.1056
Largest diff. peak/hole, e·$Å^{-3}$	0.250/-0.174	0.240/-0.230	0.194/-0.236	0.200/-0.213
CCDC Number	2413511	2413509	2422304	2413508

[a] $R_1 = \sum|F_o - |F_c||/\sum(F_o)$; [b] $wR_2 = (\sum[w(F_o^2 - F_c^2)^2]/\sum[w(F_o^2)^2]^{1/2}$
